# Artificial intelligence models Traditional Chinese Medicine principles to integrate with biomedicine

**DOI:** 10.1002/imt2.70185

**Published:** 2026-10-20

**Authors:** Haoran Li, Xingye Cheng, Ziyang Huang, Jingyuan Luo, Qianqian Xu, Qiguang Zhao, Tianchen Guo, Yumeng Zhang, Linda L. D. Zhong, Zhaoxiang Bian, Leihan Tang, Aiping Lyu, Liang Tian

**Affiliations:** ^1^ Department of Physics Hong Kong Baptist University Hong Kong SAR China; ^2^ School of Chinese Medicine, Hong Kong Baptist University Hong Kong SAR China; ^3^ Vincent V.C. Woo Chinese Medicine Clinical Research Institute, Hong Kong Baptist University Hong Kong SAR China; ^4^ Centre for Chinese Herbal Medicine Drug Development, Hong Kong Baptist University Hong Kong SAR China; ^5^ School of Biological Sciences, Nanyang Technological University Singapore Singapore; ^6^ The Chinese Medicine Hospital of Hong Kong Hong Kong SAR China; ^7^ Center for Interdisciplinary Studies and School of Science, Westlake University Hangzhou China; ^8^ State Key Laboratory for the Modernization of Classical and Famous Prescriptions of Chinese Medicine, Jiangxi University of Chinese Medicine Nanchang China; ^9^ Institute of Systems Medicine and Health Sciences, Hong Kong Baptist University Hong Kong SAR China; ^10^ Institute of Computational and Theoretical Studies, Hong Kong Baptist University Hong Kong SAR China

## Abstract

Using 26,339 model‐eligible Traditional Chinese Medicine (TCM) formula records, we trained an autoencoder following TCM's compression–expansion logic to learn the TCM embedding space (TCM‐ES), quantifying symptom‐pattern–formula mappings. TCM‐ES aligns with conventional TCM principles not used in training. Biomedical entities were mapped post hoc into the fixed space, supporting formula prediction, retrospective clinical analyses, and exploratory therapeutic prioritization.

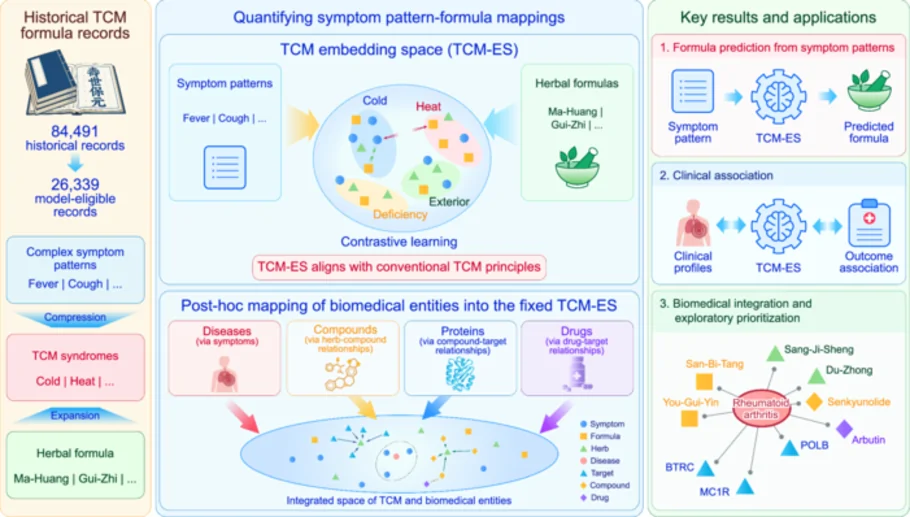

To the Editor,

Modern biomedicine has advanced our understanding of human diseases and accelerated therapeutic development. Conventional reductionist or bottom‐up approaches seek to connect clinical manifestations and symptoms to genetic or molecular causes, informing target selection and therapy development. While effective in many contexts, these approaches face challenges in complex and chronic diseases, including system complexity, dimensionality, and inter‐individual differences in therapeutic responses [1]. Traditional Chinese Medicine (TCM) diagnostic and therapeutic principles provide a complementary holistic and top‐down perspective, in which complex patient symptom patterns are compressed into syndromes, and subsequently expanded into personalized multi‐herb therapies [2]. However, the qualitative nature of these principles has hindered their systematic quantitative investigation and integration with modern biomedicine.

Previous computational approaches have employed network‐based models built largely on pairwise relationships [3], whereas deep‐learning models can address high dimensionality and nonlinearity but often lack interpretability [4]. Motivated by TCM's compression–expansion logic, we conceptualized its diagnostic–therapeutic process as an encoding–decoding mechanism in artificial intelligence (AI) models [5], with syndromes acting as a low‐dimensional bottleneck linking symptom‐pattern and herb‐combination spaces. We therefore trained a Transformer‐based autoencoder on ancient and classical TCM formula records using only matched symptom patterns and prescribed formulas [6], excluding syndrome labels and explicit TCM‐principle annotations from training. The resulting shared low‐dimensional representation, termed the TCM embedding space (TCM‐ES), provides a data‐driven quantitative representation of traditionally qualitative mappings between symptom patterns and herbal formulas, and a basis for integration with modern biomedicine.

The TCM‐ES showed organization aligned with conventional TCM principles and supported downstream formula prediction and retrospective clinical analyses. After biomedical entities were mapped into the fixed TCM‐ES through established relationships with TCM entities, its principal directions showed associations with key biological functions, while its embedding relationships were concordant with the human protein–protein interaction (PPI) network. The integrated TCM‐ES provided a symptom‐pattern‐based perspective on disease relationships and supported exploratory therapeutic candidate prioritization. We also developed a web‐based toolbox integrating data browsing, customized TCM representation, candidate‐formula generation and retrieval, model‐attention visualization, and knowledge‐graph‐based exploration of TCM and mapped biomedical entities (Figure 1A, Figure S1).

**FIGURE 1:**
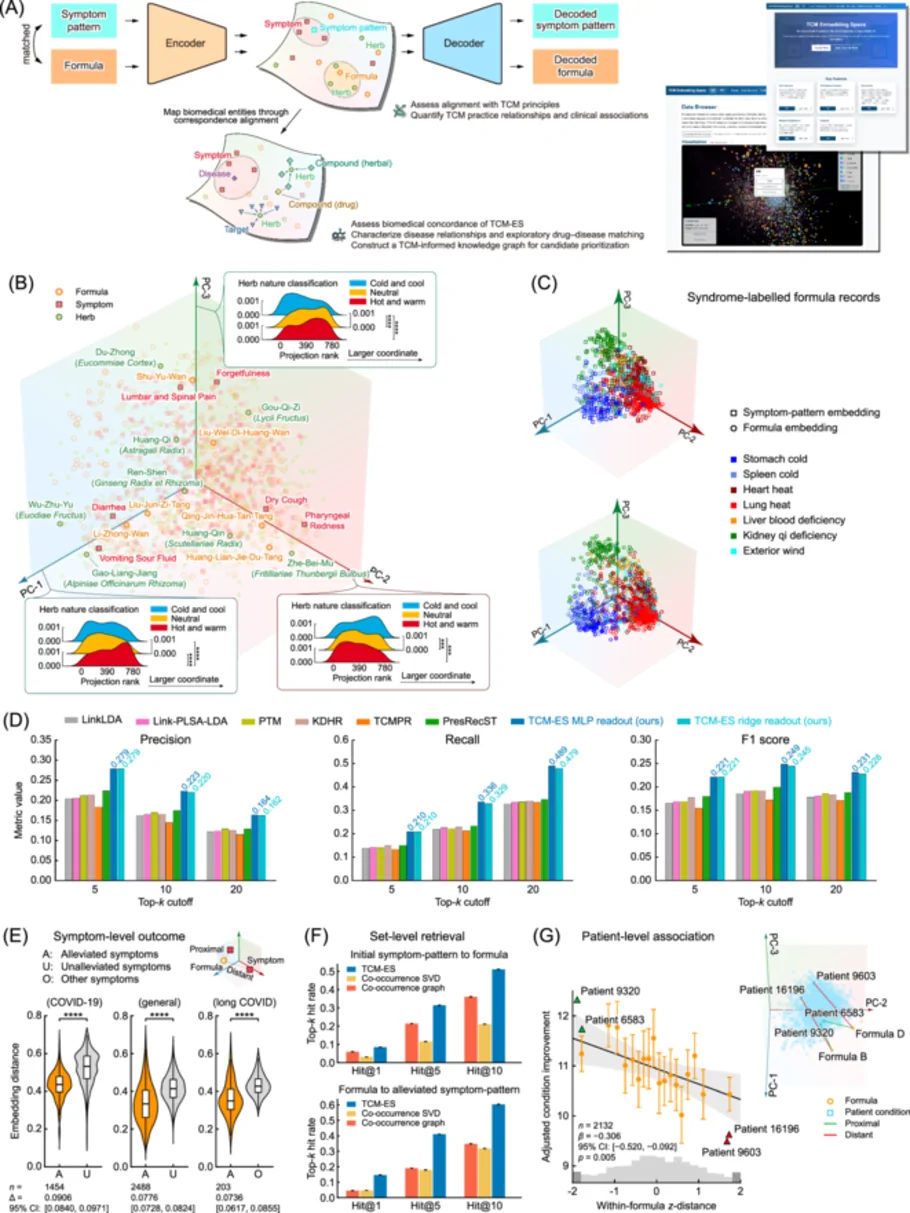
The Traditional Chinese Medicine embedding space (TCM‐ES) shows alignment with conventional TCM principles and supports quantitative analysis of TCM practice. (A) Overview of the framework. Matched symptom patterns and herbal formulas are embedded into a shared low‐dimensional space and reconstructed through the corresponding decoders. The resulting bottleneck space is defined as the TCM‐ES (Note S2). Biomedical entities are mapped post hoc into the fixed TCM‐ES through their established relationships with TCM entities (Note S6). A web‐based toolbox enables the generation and exploration of TCM‐ES embeddings (Note S8). (B) TCM‐ES projected onto the first three principal components (PCs), showing representative symptoms, herbs, and formula embeddings. Insets show the projection‐rank distributions of herbs classified by TCM‐defined natures (cold/cool, neutral, and warm/hot) along the corresponding PCs. Herb natures were not used for model training. (C) Syndrome‐associated organization of TCM formula records in TCM‐ES. Each point represents the embedding of a symptom‐pattern (top) or a formula (bottom) from a record annotated with one of seven representative syndromes. Points are colored according to the differentiated syndrome. Syndrome labels were not used for model training. Across all syndrome‐annotated records, syndrome‐associated neighborhood consistency (the proportion of the *k* = 20 nearest neighbors sharing at least one syndrome label) exceeded the syndrome‐label permutation expectation for both symptom‐pattern embeddings (0.170 vs. 0.044) and formula embeddings (0.173 vs. 0.044; 1000 permutations; Holm‐adjusted *p*_perm_ = 0.002 for both). Held‐out linear readouts from the frozen embeddings yielded macro‐AUROCs of 0.844 and 0.851, respectively (Figure S14D). (D) External formula‐prediction performance on the public TCM‐PD benchmark. Frozen TCM‐ES symptom‐pattern embeddings were used to fit either an MLP or linear ridge readout, and Precision@*k*, Recall@*k*, and F1@*k* are shown at *k* = 5, 10, and 20. Performance of six herbal‐formula recommendation models on TCM‐PD is shown for comparison. (E) Formula‐to‐symptom relationships in three clinical datasets. Embedding distances from prescribed formulas to symptoms subsequently documented as alleviated were compared with unalleviated symptoms in the COVID‐19 and general TCM cases, and with other symptoms in the long‐COVID cases. Analyses required at least one alleviated and one comparison symptom. Two cases were excluded for lacking an unalleviated symptom (*n* = 2488). Prescribed formula herbs also had to be fully covered by the TCM‐ES herb vocabulary. The filtering resulted in 1454 COVID‐19 cases, 2488 general TCM cases, and 203 long‐COVID consecutive visit pairs. Paired mean differences (comparison minus alleviated) are shown with 95% confidence intervals. ****p < 0.0001, paired two‐sided *t*‐test. (F) Bidirectional set‐to‐set retrieval in the general TCM clinical cases. First, initial symptom patterns were used to retrieve the formulas prescribed in the corresponding cases (top). Then, the prescribed formulas were used to retrieve the alleviated‐symptom patterns documented at follow‐up (bottom). TCM‐ES uses encoder‐derived complete‐set embeddings, whereas the SVD and graph co‐occurrence baselines use pooled embeddings of individual symptoms or herbs. Bars show Hit@1, Hit@5, and Hit@10. Error bars indicate the standard error across repeated candidate samplings. (G) Adjusted retrospective association between patient‐condition–formula embedding proximity and subsequent condition improvement in 2132 COVID‐19 cases. Patient‐condition–formula distance was calculated between the initial symptom‐pattern and prescribed‐formula embeddings and standardized within prescribed‐formula groups. Condition improvement was quantified as the reduction in total symptom score from the initial to follow‐up visit and adjusted for initial total symptom score, initial symptom count, follow‐up interval, age, sex, and prescribed‐formula fixed effects. Orange points show the mean adjusted improvement across equal‐frequency distance bins with standard errors. The blue line shows the association estimated from the continuous multivariable model, and the shaded region indicates its 95% confidence interval.

## A DATA‐DRIVEN ENCODING–DECODING FRAMEWORK FOR QUANTIFYING TCM PRACTICE

To quantify the symptom‐pattern–formula mappings, we used a corpus of 84,491 ancient and classical TCM formula records, of which 26,339 model‐eligible records were used to develop a Transformer‐based autoencoder (Note S1; Figure S2 and Tables S1, S2). We only used matched symptom patterns and corresponding prescribed formulas, without syndrome differentiation labels or explicit TCM‐principle annotations as training inputs or targets. The model combined bottleneck encoding to compress symptom patterns and formulas into low‐dimensional representations [5], contrastive learning to align matched symptom‐pattern–formula pairs within a shared space [7], and Transformer‐based attention to model context‐dependent, combinatorial, and higher‐order relationships within complete symptom patterns and formulas (Figure 1A; Note S2; Figures S3, S4) [6].

Using the trained encoders, individual symptoms, herbs, complete symptom patterns, and formulas could be represented as vectors within the same low‐dimensional coordinate system. We define this as the TCM embedding space (TCM‐ES, Figure 1B; Note S2). Controlled ablations showed complementary contributions of major model components to reconstruction and TCM‐ES organization (Note S3; Figures S5–S11 and Table S3). The web‐based toolbox provides access to curated data and TCM‐ES embeddings and supports downstream analyses (Figure 1A).

## TCM‐ES SHOWS ORGANIZATION ALIGNED WITH CONVENTIONAL TCM PRINCIPLES

Principal component analysis (PCA) revealed a compact but multiaxial organization of TCM‐ES, with most variation concentrated in a few leading principal components (PCs) (Figure S9). TCM‐ES geometry and leading PCs were reproducible across ten independently trained models after PC alignment (Note S3; Figures S12, S13). The first three PCs were associated with distinct representative symptoms and herb distributions (Figure 1B and Table S4). PC1 was characterized mainly by gastrointestinal symptoms and hot/warm herbs, PC2 by respiratory and heat‐related symptoms and cold/cool herbs, and PC3 by weakness‐ and deficiency‐related symptoms and predominantly neutral or warm/hot herbs. These symptom–herb distributions broadly corresponded to the conventional Cold‐, Heat‐, and Deficiency‐associated organization of TCM syndromes, respectively [8].

Syndrome‐associated organization was also apparent in whole symptom patterns and formulas. Diagnostically, symptom‐pattern embeddings from formula records differentiated as typical TCM syndromes occupied distinguishable but continuous regions in the TCM‐ES, aligning with Cold, Heat, and Deficiency syndrome categories (Figure 1C, top). Therapeutically, formula embeddings showed broadly similar syndrome‐associated organization (Figure 1C, bottom). Syndrome–latent‐dimension block‐wise correspondence and additional TCM‐principle‐associated organization were also observed (Figures S14, S15). This organization was disrupted by shuffling symptom‐pattern–formula matching, and was not fully reproduced by co‐occurrence‐based embeddings (Note S3; Figures S16–S18), indicating higher‐order combinatorial structure beyond pairwise co‐occurrence in TCM‐ES. Although syndrome labels and explicit principle annotations were excluded from training, this alignment provides a quantitative basis for characterizing the traditionally qualitative symptom‐pattern–formula mappings underlying TCM principles.

## TCM‐ES FACILITATES DOWNSTREAM FORMULA PREDICTION

On the held‐out test set of TCM formula records, TCM‐ES embeddings improved formula prediction over raw symptom inputs and shuffled embeddings, suggesting formula‐relevant organization in symptom‐pattern embeddings (Note S4; Figures S19, S20).

We evaluated external transfer on the public TCM‐PD benchmark [4, 9] (Note S4), where frozen TCM‐ES embeddings achieved numerically higher performance across evaluated metrics than multiple published models on the formula‐prediction task. We mapped TCM‐PD symptoms and herbs to standardized TCM‐ES vocabularies and used the benchmark training and test sets. Benchmark test records highly similar to TCM‐ES training records were excluded to minimize data leakage. With the TCM‐ES encoder frozen, we fitted linear ridge or nonlinear MLP readouts on benchmark training records.

Both readouts showed strong performance. The linear ridge readout achieved P@5 = 0.279 [0.271–0.286], R@20 = 0.479 [0.470–0.487], and F1@20 = 0.228 [0.223–0.232] (95% CIs), compared with the highest displayed published values of 0.224, 0.347, and 0.188, respectively. Across all nine Precision@*k*, Recall@*k*, and F1@*k* metrics at *k* = 5, 10, and 20, both TCM‐ES readouts yielded numerically higher values than the six published TCM‐PD models (Figure 1D), although the evaluation settings were not fully matched. The nonlinear MLP only modestly improved over the linear ridge readout, indicating that substantial formula‐prediction information was linearly accessible within the frozen TCM‐ES (Figure 1D).

## TCM‐ES LINKS SPATIAL RELATIONSHIPS TO CLINICAL OUTCOMES

Across three clinical datasets with treatment and follow‐up information, TCM‐ES spatial relationships corresponded to subsequent clinical outcomes. The datasets included: (1) 5095 general TCM clinical cases with initial and follow‐up visits; (2) 144 long‐COVID patients contributing 598 follow‐up visits [10]; and (3) 2132 COVID‐19 clinical cases with standardized symptom assessments at initial and follow‐up visits [11]. These datasets recorded initial symptom patterns, prescribed formulas, and follow‐up symptom changes, enabling analyses of how TCM‐ES relationships corresponded to subsequent clinical outcomes at progressively broader levels. The datasets covered diverse patient characteristics, symptom profiles, and prescribed herbs and formulas (Note S5; Figures S21–S25 and Tables S5–S9).

At the individual‐symptom level, prescribed formulas were consistently closer in TCM‐ES to symptoms subsequently documented as alleviated than to unalleviated or other symptoms across all three datasets (Figure 1E). Analyses using co‐occurrence‐based embeddings showed that such formula–symptom proximity reflected subsequent symptom outcomes beyond individual symptom frequency and pairwise co‐occurrence (Figure S26).

At symptom pattern or formula level, holistic TCM‐ES embeddings enabled more accurate set‐to‐set retrieval than pooled symptom or herb embeddings from co‐occurrence‐based models. Using the general clinical dataset, we compared TCM‐ES with two co‐occurrence‐based embeddings in complementary retrieval tasks. When the initial symptom pattern was used to retrieve the corresponding prescribed formula, TCM‐ES achieved higher Hit@1, Hit@5, and Hit@10 than both co‐occurrence baselines (Figure 1F, top). The complementary retrieval from the prescribed formula to the alleviated‐symptom pattern showed the same result (Figure 1F, bottom). These results show the advantage of TCM‐ES in capturing higher‐order, holistic mappings between complete symptom patterns and formulas.

At the whole‐patient level, closer patient‐condition–formula relationships were associated with greater subsequent condition improvement in 2132 COVID‐19 cases after accounting for differences among prescribed formulas and patient‐level factors. The embedding distance between each initial patient condition (symptom pattern) and the prescribed formula was standardized within formula groups and evaluated in a multivariable model adjusting for initial total symptom score, initial symptom count, follow‐up interval, age, sex, and prescribed‐formula fixed effects (Note S5). Greater distance was associated with less subsequent condition improvement, quantified by total symptom‐score reduction (*β* = −0.306 per 1‐SD increase in within‐formula distance, 95% CI −0.520 to −0.092, *p* = 0.005; Figure 1G). This negative association was broadly similar across rolling initial‐to‐follow‐up intervals, patient‐characteristic subgroups, and formula groups, despite variation in magnitude and precision (Figure S27).

## MAPPING BIOMEDICAL ENTITIES INTO TCM‐ES REVEALS BIOMEDICAL CONCORDANCE

Keeping the core TCM‐ES fixed, we incorporated modern biomedical entities through established relationships with TCM entities (Figure 1A; Note S6), yielding an integrated TCM‐ES (Figure 2A). Human diseases were represented through associated symptom patterns, whereas herbal compounds and target proteins were mapped through corresponding herbs. FDA‐approved drugs were mapped through structural similarity to embedded herbal compounds, with analyses restricted to high‐confidence mappings within the supported herbal‐compound training domain (Table S10). Notably, biomedical information was not used to train the core TCM‐ES, but was introduced only after the core space was fixed.

**FIGURE 2:**
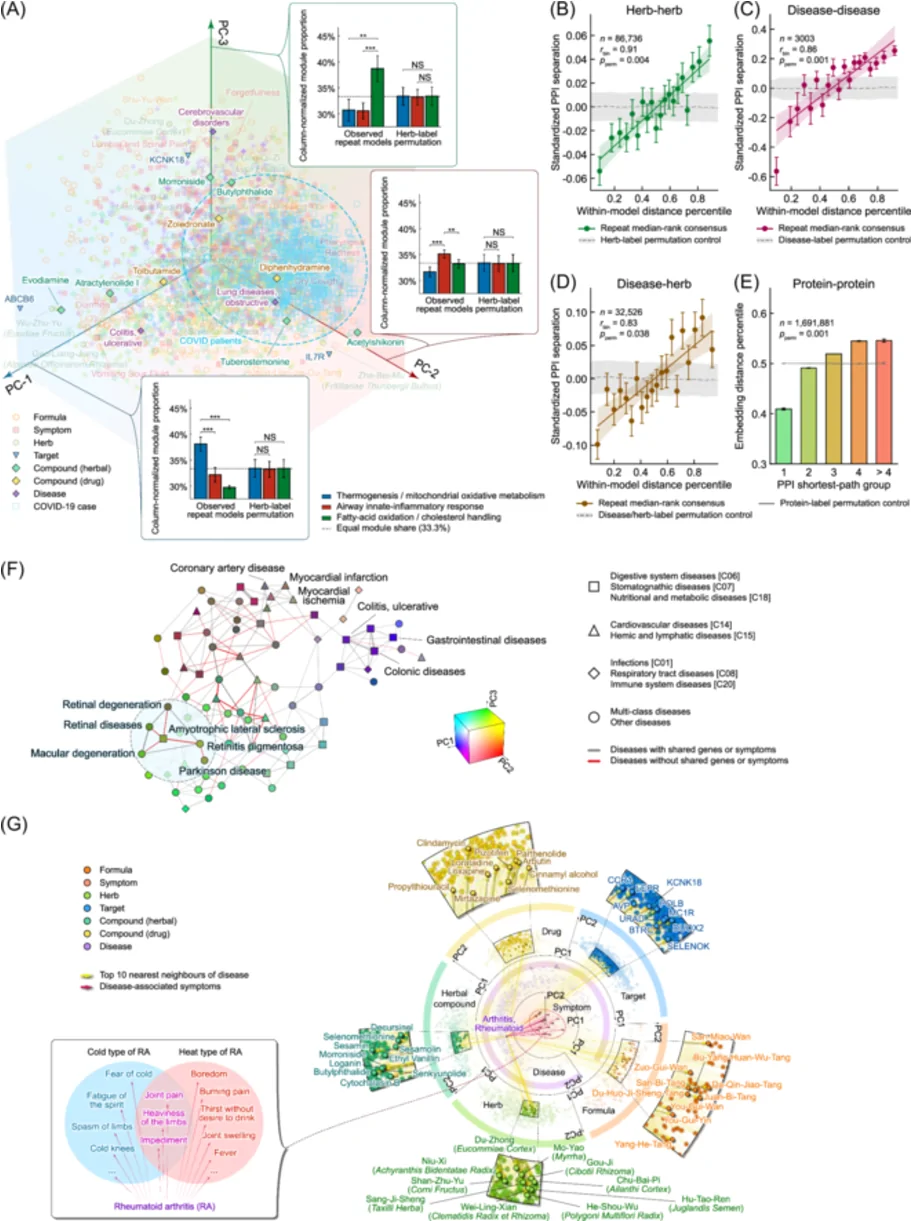
The integrated Traditional Chinese Medicine embedding space (TCM‐ES) shows biomedical concordance and supports exploratory analyses of disease relationships and therapeutic candidates. (A) Biomedical entities mapped into the fixed TCM‐ES through their established relationships with TCM entities. The resulting integrated TCM‐ES is visualized on PC1–PC3. Insets show the relative exposure of the top‐ranked herbs along the corresponding PCs to three literature‐supported Gene Ontology (GO) Biological Process modules: thermogenesis and mitochondrial oxidative metabolism, airway innate‐inflammatory responses, and fatty‐acid oxidation and cholesterol handling. Module exposure was calculated from GO‐annotated target‐protein profiles associated with the herbs (Note S7). Bars show the mean ± standard deviation across 10 independently trained and PC‐aligned models for the observed exposures and herb‐label permutation controls. The corresponding PC‐associated module was compared with the two alternative modules using paired two‐sided *t*‐tests across the 10 independently trained models, with Holm correction across the six prespecified within‐PC comparisons (two comparisons for each of PC1–PC3). Global concordance between pairwise distances in TCM‐ES and relationships in the human protein–protein interaction (PPI) network for herb–herb (B), disease–disease (C), and disease–herb (D) pairs. For each entity pair, embedding‐distance percentiles were summarized by the median across ten independently trained models. PPI relationships were quantified by standardized module separation between the corresponding herb‐target and/or disease‐associated gene sets. Points and error bars show mean ± standard error within equal‐frequency bins of embedding‐distance percentile. Lines and shaded regions show linear fits and 95% confidence intervals. *r*_bin_ denotes the Pearson correlation across the displayed bin means. *p*_perm_ values were obtained from 1000 two‐sided permutation tests using the same statistic, and *n* denotes the number of underlying entity pairs. Pairs sharing an entity are not statistically independent. Gray lines show the corresponding permutation controls. (E) Protein–protein concordance between TCM‐ES proximity and PPI shortest‐path distance. Bars show the mean embedding‐distance percentile for protein pairs grouped by PPI shortest‐path length. *p*_perm_ was obtained from 1000 two‐sided PPI‐label permutations, and *n* denotes the number of connected protein pairs. Pairs sharing a protein are not statistically independent. The gray line shows the corresponding permutation control. For panels (B–E), permutation controls are based on identity shuffling with degree‐preserving PPI permutation. Node‐level leave‐one‐out analyses confirmed robustness to individual entities (Note S7). (F) *k*‐nearest‐neighbor network (*k* = 7) of disease relationships based on disease embedding proximity in TCM‐ES. Nodes represent diseases, node shapes indicate MeSH categories, and node colors reflect their positions along the leading TCM‐ES PCs. Edge width increases with embedding proximity. Gray edges connect disease pairs sharing annotated genes or symptoms, whereas red edges indicate neighboring disease pairs sharing neither. (G) Integrated TCM‐ES knowledge graph for exploratory therapeutic‐candidate prioritization. Diseases are connected to neighboring molecular and therapeutic entities in TCM‐ES, including target proteins, herbal compounds, herbs, formulas, and supported modern drugs. Rheumatoid arthritis (RA) is shown as an illustrative example, with nearby entities displayed across the corresponding data types. The inset shows distinct symptom profiles of clinically defined Cold‐ and Heat‐type RA. Supporting evidence for selected RA‐associated targets and therapeutic candidates is provided in Table S12.

The reproducible PCs of the integrated TCM‐ES showed distinct associations with biological processes. Across 10 independently trained models, PC1 showed the highest relative exposure to a literature‐supported Gene Ontology [12] (GO) Biological Process module related to thermogenesis and mitochondrial oxidative metabolism [13], PC2 to an airway innate‐inflammatory response module including TLR4/interferon and acute‐inflammatory processes [14, 15], and PC3 to a fatty‐acid oxidation and cholesterol‐handling GO module [16, 17] (Figure 2A; Note S7; Table S11). These PC‐specific patterns were robust to herb‐selection thresholds (Figure S28), but were substantially weakened after herb‐label permutation (Figure 2A), indicating that they depended on herb embeddings in the TCM‐ES rather than on the herb–target annotation structure alone.

TCM‐ES spatial relationships among biomedical entities were concordant with their molecular relationships in the human PPI network [18] (Note S7). PPI proximity among herbs and diseases was quantified by module separation between herb‐target and/or disease‐associated gene sets [19] (Figure S29). Across 10 independently trained models, herbs embedded closer in TCM‐ES tended to have more proximal target modules, diseases embedded closer tended to have more proximal disease‐gene modules, and similar correspondence held for disease–herb relationships (Figure 2B–D). Consistently, proteins embedded closer in TCM‐ES tended to be connected by shorter PPI paths (Figure 2E). These relationships also held among local embedding neighbors and across training seeds (Note S7; Figures S30, S31), but were substantially weakened by identity shuffling and degree‐preserving PPI permutation (Figure 2B–E, gray lines; Note S7), indicating that the concordance required the correct correspondence among embedding positions, biomedical entity identities, and PPI‐network positions.

Although trained without biomedical information, TCM‐ES shows systematic concordance with established biological processes and molecular relationships, providing a quantitative basis for TCM‐informed biomedical characterization and hypothesis generation.

## TCM‐INFORMED DISEASE CHARACTERIZATION AND EXPLORATORY THERAPEUTIC CANDIDATE PRIORITIZATION

The integrated TCM‐ES provided a symptom‐pattern‐based perspective on disease organization. We constructed a *k*‐nearest‐neighbor network based on disease embedding proximity (Figure 2F; Note S8). The network grouped many diseases with related MeSH categories (Figure S32), while also identifying neighboring pairs sharing neither annotated genes nor symptoms (Figure 2F, red edges; Figure S33). For example, one local cluster contained retinal degenerative and neurodegenerative diseases, consistent with reported connections between these disease groups (Figure 2F, blue area) [20].

Symptom‐based disease embeddings were incorporated into a TCM‐informed knowledge graph linking them to neighboring target proteins, herbal compounds, herbs, formulas, and supported modern drugs (Figure 2G; Notes S6, S8). These proximity‐based relationships supported exploratory target and therapeutic‐candidate prioritization, while also providing an alternative embedding‐based perspective on drug–disease matching (Figures S34, S35; Note S9). Using rheumatoid arthritis (RA) as an example, the knowledge graph prioritized multiple nearby molecular and therapeutic candidates in TCM‐ES (Figure 2G). Although not directly demonstrating efficacy, the analysis prioritized several targets and therapeutic candidates supported by previous studies (Table S12). More detailed TCM symptom profiles from a previous study involving 21 female patients further refined the RA embedding, yielding distinct representations and neighboring candidate profiles for the reported Cold‐ and Heat‐pattern groups (Figure S36). This illustrates how TCM‐informed symptom characterization can provide a complementary view of disease heterogeneity and generate testable hypotheses for experimental and clinical investigation.

In conclusion, TCM‐ES provides a quantitative basis for representing empirical symptom‐pattern–formula relationships in TCM practice and relating them to biomedical information. Incorporating biomedical prior knowledge into subsequent model training could extend TCM‐ES into a jointly informed framework linking symptom‐defined patient conditions with molecular and therapeutic information. Future experimental and prospective studies can evaluate these relationships and support TCM‐informed therapeutic discovery, personalized treatment, and closer integration with modern biomedicine.

## AI STATEMENT

OpenAI ChatGPT (GPT‐5.6 Sol) was used to assist with English‐language editing, wording refinement, and manuscript formatting. All scientific analyses and interpretations were performed by the authors, and the final manuscript was reviewed and verified for accuracy by the authors.

## AUTHOR CONTRIBUTIONS

**Haoran Li**: Investigation; validation; formal analysis; visualization; writing—original draft; writing—review and editing; methodology; data curation; software. **Xingye Cheng**: Methodology; data curation; investigation; validation; formal analysis; visualization; software. **Ziyang Huang**: Data curation. **Jingyuan Luo**: Data curation. **Qianqian Xu**: Data curation. **Qiguang Zhao**: Data curation. **Tianchen Guo**: Data curation. **Yumeng Zhang**: Data curation. **Linda L. D. Zhong**: Data curation. **Zhaoxiang Bian**: Data curation; resources. **Leihan Tang**: Conceptualization; project administration; supervision; writing—review and editing. **Aiping Lyu**: Conceptualization; supervision; project administration; writing—review and editing; resources; funding acquisition. **Liang Tian**: Conceptualization; supervision; project administration; writing—review and editing; writing—original draft; resources; funding acquisition.

## CONFLICT OF INTEREST STATEMENT

The authors declare no conflicts of interest.

## ETHICS STATEMENT

For long COVID cases, the dataset was collected under the approval of corresponding Research Ethics Committee(s) in HK and followed the guidelines in the Declaration of Helsinki and ICH Good Clinical Practice. The study was reviewed and approved by the following research ethics committees: (i) Clinical Research Ethics Panel, Research Ethics Committee, Hong Kong Baptist University (Approval no. REC/19–20/0468); (ii) Joint Chinese University of Hong Kong‐New Territories East Cluster Clinical Research Ethics Committee, Hospital Authority New Territories East Cluster, Hospital Authority (CREC Ref. no. 2020.288); and (iii) Research Ethics Committee (Kowloon Central/Kowloon East), Hospital Authority (Ref.: KC/KE‐20–0240/ER‐1). The protocol was registered at the ClinicalTrials.gov (NCT04544605) on 3 September 2020 and published with open access. Informed consent was obtained from all participants. For general TCM cases and COVID‐19 cases, all patients provided informed consent, and all personal data were de‐identified. As this retrospective study used anonymized data, no ethical approval was sought.

## Supporting information


**Figure S1:** Conceptual framework and empirical diagnostic–therapeutic relationships in historical TCM records.
**Figure S2:** Frequency distributions and corpus coverage of TCM herbs, symptoms and syndromes.
**Figure S3:** The model for the TCM‐ES construction and biomedical entities integration.
**Figure S4:** Loss curves during main model training.
**Figure S5:** Design of the TCM‐ES ablation models.
**Figure S6:** Distinct contributions of attention, pretrained symptom semantics, and contrastive learning to TCM‐ES representation structure.
**Figure S7:** Attention patterns for TCM formula records and disease‐associated symptom patterns.
**Figure S8:** Attention‐guided perturbation of symptom‐pattern–formula reconstruction in held‐out formula records.
**Figure S9:** Dimensions of the embeddings.
**Figure S10:** Normalization of the embedding distance.
**Figure S11:** Principal TCM‐ES results are retained after retraining with the non‐leakage split.
**Figure S12:** Global and local stability of TCM‐ES geometry across repeated training.
**Figure S13:** Alignment of principal components across repeated TCM‐ES models.
**Figure S14:** Quantitative characterization and robustness of syndrome‐associated organization in TCM‐ES.
**Figure S15:** Additional conventional TCM annotations are associated with the organization of TCM‐ES.
**Figure S16:** Hierarchical explanatory factors of embedding geometry.
**Figure S17:** Principal‐component analysis of the original TCM‐ES, null models, and co‐occurrence‐based baseline embeddings.
**Figure S18:** Syndrome‐associated organization of formula record embeddings across semantic‐only, null, and co‐occurrence‐based baseline embeddings.
**Figure S19:** Examples of formula decoding based on symptom patterns using the trained model.
**Figure S20:** Held‐out formula prediction from symptom patterns.
**Figure S21:** Patient characteristics and symptom distributions in the clinical datasets.
**Figure S22:** Prescription frequencies of herbs and standard formulas in the clinical datasets.
**Figure S23:** Semantic coverage of symptom terms used for biomedical and clinical mapping.
**Figure S24:** Consistency of TCM‐ES geometry with observed clinical and TCM practice relationships.
**Figure S25:** Consistency of TCM‐ES geometry with observed co‐occurrence patterns using NPMI.
**Figure S26:** Formula–symptom ranking in clinical cases and sensitivity to symptom frequency.
**Figure S27:** Sensitivity analyses of the adjusted patient‐condition–formula association in COVID‐19 cases.
**Figure S28:** Sensitivity of PC‐associated GO‐module composition to the number of selected herbs.
**Figure S29:** Connectivity of disease‐associated gene and herb‐target modules in the human PPI network.
**Figure S30:** Local concordance between TCM‐ES neighbourhoods and PPI‐network proximity.
**Figure S31:** Training‐seed stability of embedding–PPI concordance.
**Figure S32:** Diseases with similar MeSH classifications are positioned closer in TCM‐ES.
**Figure S33:** Robustness of the TCM‐ES disease network to the number of nearest neighbours.
**Figure S34:** Exploratory drug–disease relationships in TCM‐ES and their association with a measure derived from the FDA Adverse Event Reporting System (FAERS).
**Figure S35:** Sensitivity analyses of exploratory drug–disease relationships in TCM‐ES.
**Figure S36:** TCM‐informed characterization of Cold‐ and Heat‐pattern RA groups reported in a previous study of 21 female patients.

**Table S1:** The TCM concepts.
**Table S2:** An example of TCM formula records.
**Table S3:** Cross‐split record similarity under alternative similarity criteria and thresholds.
**Table S4:** Representative TCM and biomedical entities along leading TCM‐ES PCs.
**Table S5:** Summary of TCM, biomedical and clinical datasets.
**Table S6:** An example of general TCM clinical cases.
**Table S7:** An example of long COVID clinical cases.
**Table S8:** An example of COVID‐19 clinical cases.
**Table S9:** Compositions of standard formulas used in the COVID‐19 cases.
**Table S10:** Mapping‐confidence assessment of approved‐drug embeddings generated from the herbal‐compound training domain.
**Table S11:** GO modules used to evaluate biological concordance of TCM‐ES PC1‐PC3.
**Table S12:** Prioritized molecular and therapeutic candidates for rheumatoid arthritis and supporting evidence.

## Data Availability

The full summary statistics to support the findings of this study are included within the supporting information files. The data used for the main figures generated in this study and example data have been deposited on GitHub: https://github.com/kulasimu/TCM-embedding-space. Due to ethical and legal restrictions, deidentified individual participant clinical data cannot be made publicly available. All clinical data are available upon request to the corresponding author and subject to local rules and regulations. The scripts used in this study, together with the source code, trained model checkpoints, standardized symptom and herb dictionaries, train/validation/test split files, and processed TCM‐ES embeddings, can also be found on GitHub: https://github.com/kulasimu/TCM-embedding-space. Third‐party resources, including the public TCM formula databases, HIT 2.0, DrugBank, MeSH/Mayo Clinic, openFDA, ClinCalc, OMIM/GWAS, and the PPI network from previous studies, together with their versions and content details, are documented in the supporting information. An online version of the toolbox is available at https://www.tcmes.net/. Supplementary materials (figures, notes, tables, graphical abstract, slides, videos, Chinese translated version, and updated materials) may be found in the online DOI or iMeta Science (http://www.imeta.science).
