# Supplementary material for: Artificial intelligence models Traditional Chinese Medicine principles to integrate with biomedicine
Source: Imeta. 2026 Oct 20:e70185. Online ahead of print. doi: 10.1002/imt2.70185 (PMC13683242; doi:10.1002/imt2.70185)
Supplement: Supplementary file 1 [file IMT2-9999-e70185-s001.docx]

**Supporting Information to**

# Artificial intelligence models Traditional Chinese Medicine principles to integrate with biomedicine

**Running title:** AI framework for quantifying TCM principles

Haoran Li^1#^, Xingye Cheng^1#^, Ziyang Huang^1^, Jingyuan Luo^2,3,4^, Qianqian Xu^2,3^, Qiguang Zhao^2^, Tianchen Guo^2^, Yumeng Zhang^2^, Linda LD Zhong^5^, Zhaoxiang Bian^3,4,6^, Leihan Tang^7*^, Aiping Lyu^2,3,8,9*^, Liang Tian^1,9,10*^

^1^Department of Physics, Hong Kong Baptist University, Hong Kong SAR, China

^2^School of Chinese Medicine, Hong Kong Baptist University, Hong Kong SAR, China

^3^Vincent V.C. Woo Chinese Medicine Clinical Research Institute, Hong Kong Baptist University, Hong Kong SAR, China

^4^Centre for Chinese Herbal Medicine Drug Development, Hong Kong Baptist University, Hong Kong SAR, China

^5^School of Biological Sciences, Nanyang Technological University, Singapore 637551, Singapore

^6^The Chinese Medicine Hospital of Hong Kong, Hong Kong SAR, China

^7^Center for Interdisciplinary Studies and School of Science, Westlake University, Hangzhou, China

^8^State Key Laboratory for the Modernization of Classical and Famous Prescriptions of Chinese Medicine, Jiangxi University of Chinese Medicine, Nanchang 330004, China

^9^Institute of Systems Medicine and Health Sciences, Hong Kong Baptist University, Hong Kong SAR, China

^10^Institute of Computational and Theoretical Studies, Hong Kong Baptist University, Hong Kong SAR, China

^#^These authors contributed equally: Haoran Li, Xingye Cheng.

*Correspondence: [tangleihan@westlake.edu.cn](mailto:tangleihan@westlake.edu.cn) (Leihan Tang), [aipinglu@hkbu.edu.hk](mailto:aipinglu@hkbu.edu.hk) (Aiping Lyu), [liangtian@hkbu.edu.hk](mailto:liangtian@hkbu.edu.hk) (Liang Tian).

# SUPPORTING NOTES

## Note S1 TCM concepts and historical formula-record data

### Traditional Chinese Medicine (TCM) concepts and annotations

Syndrome and syndrome differentiation

The diagnostic process in TCM seeks to identify a patient’s pathological state to guide treatment. In the absence of laboratory testing in ancient times, TCM relied heavily on symptom observation, with diagnosis based on observed patients’ symptom patterns. This diagnosis process is known as syndrome differentiation, resulting in what is referred to as a syndrome. Syndrome differentiation views the human body as an integrated and organically coordinated system. Consequently, while attending to localized symptoms, syndrome differentiation also emphasizes the patient’s overall condition and aims to gather comprehensive and relevant clinical information (Figure S1A). In summary, syndrome differentiation refers to the process of analysing and synthesizing various types of clinical information under the guidance of TCM principles, leading to a characterization of the patient’s current condition (e.g., affected organ and underlying pathogenesis) and resulting in a comprehensive characterization of the patient’s TCM syndrome.

Throughout its historical development, TCM has established multiple systems of syndrome differentiation. Among these, organ-based syndrome differentiation is the most widely applied. Accordingly, our syndrome data are structured within this system. In this system, each syndrome consists of two parts: (ⅰ) affected organs; and (ⅱ) the underlying pathogenesis.

Organs

The TCM organs considered in this study include the Heart, Heart Spirit (Pericardium), Lung, Spleen, Liver, Kidney, Stomach, Gallbladder, Small Intestine, Large Intestine, Bladder, and Uterus. As some syndromes manifest externally on the limbs or skin, we also include “Exterior” as a location for completeness. Unless otherwise stated, these 13 organs and locations are collectively referred to as “organs” for simplicity (see TCM formula record data and preprocessing for details).

It should be noted that the definitions of organs in TCM differ from those of their anatomical counterparts in modern medicine. This is because TCM constructs its model of the human body through inductive reasoning. For example, the digestion and absorption of food in TCM are attributed to the Spleen’s function of transportation, which from the modern medicine perspective involves functions of both the pancreas and small intestine. Similarly, the Heart in TCM does not solely refer to the anatomical heart but also governs mental activities and consciousness.

Pathogenesis

TCM syndrome pathogenesis refers to the nature or underlying causative factor of a pathological condition, such as Wind, Cold, or Qi Deficiency [1]. It serves as a classification criterion for syndromes based on their intrinsic characteristics. In this study, we focus on 42 distinct pathogenesis types collected from official documents (see TCM formula record data and preprocessing for details). To elucidate the concept, we introduce three common classifications of syndromes according to pathogenesis:

Cold Syndrome: Refers to syndromes caused by external Cold invasion or internal excess of Yin, typically characterized by symptoms indicating reduced physiological activity, such as aversion to cold, chilliness, and pale complexion.

Heat Syndrome: Refers to syndromes resulting from external Heat invasion or internal excess of Yang, manifested by signs of hyperactive physiological functions, including fever, thirst, restlessness, and a red complexion.

Deficiency Syndrome: Refers to syndromes marked by the depletion of essential bodily substances (e.g., Yin, Yang, Qi, Blood, Body Fluids, or Essence, etc.) and characterized by symptoms of insufficiency, weakness, fatigue, or functional decline.

Herb natures

Corresponding to the concept of pathogenesis, TCM herbs are also categorized according to their TCM-defined properties, which guide their clinical application in treating specific syndromes. The most fundamental classification is based on “herb nature”, referring to the inherent thermal property of an herb [2,3]. If an herb exhibits no distinct hot or cold tendency, it is classified as neutral. Accordingly, there are five basic herb natures: Cold, Cool, Neutral, Warm, and Hot. Each herb is assigned to one, and only one of these five categories.

Cold and Cool: Herbs with Cold or Cool nature are used to alleviate or eliminate Heat Syndromes. These two natures share similar properties, and only differ in intensity, with Cold being stronger than Cool.

Warm and Hot: Herbs with Warm or Hot nature are applied to counteract Cold Syndromes. Warm and Hot natures differ primarily in degree, with Hot being more intense.

Neutral: Refers to herbs whose thermal properties are not distinctly Cold or Hot. These herbs are generally mild in action and tend to exert a balanced, harmonizing effect.

In summary, Cold and Cool herbs are primarily used to treat Heat Syndromes, whereas Warm and Hot herbs are mainly employed to address Cold Syndromes. Syndrome organs, pathogenesis types, and herb natures are summarized in Table S1.

Herb channels

In addition to herb natures, TCM herbs are also characterized by their channel tropism, commonly referred to as “herb channels” or “channel entry”. In TCM theory, herb channels describe the tendency of an herb to act preferentially on specific organs or meridian systems in the body, thereby guiding its therapeutic application toward particular pathological locations or functional systems. This property reflects the empirical observation in TCM that different herbs may be associated with different organs or meridian systems beyond their thermal nature.

The channel attribution of an herb is typically described corresponding to the organ framework of TCM. In this study, we include 12 herb channels: Lung (肺), Kidney (肾), Liver (肝), Heart (心), Spleen (脾), Stomach (胃), Large Intestine (大肠), Small Intestine (小肠), Bladder (膀胱), Gallbladder (胆), Heart Spirit (心包), and San Jiao/Triple Burner (三焦). A single herb may have one or multiple channels, depending on its TCM indications and observed clinical actions. For example, herbs with the Lung channel are often used to treat cough, dyspnoea, or other disorders related to the respiratory system, whereas herbs having the Liver channel may be used for conditions associated with emotional dysregulation, tendons, or the circulation of blood.

It should be noted that herb channels do not imply a direct anatomical correspondence in the modern biomedical sense. Rather, they represent a functional classification system in TCM, summarizing the organs, body regions, or physiological processes with which an herb is most closely associated in clinical practice. Therefore, herb channels complement herb natures: while herb nature indicates the general thermal tendency of an herb, herb channel indicates the relative organ or system toward which its action is directed.

In summary, herb channels provide an additional dimension for understanding herb properties and for guiding formula design in TCM. Together with herb natures and other herb characteristics, channel attribution helps determine how herbs are selected and combined to match specific syndromes and affected organs.

### TCM formula record data and preprocessing

TCM formula record corpus and standardized TCM entities

TCM formula record corpus. We curated 84,491 TCM formula records from the National Database for Traditional Chinese Medicine (NDTCM) [4]. These formula records originate primarily from classical medical canons compiled by medical authorities and eminent physicians, spanning from the Han dynasty (around the 2nd century BCE) to the modern era. The dataset also includes classical formula records from Korean and Japanese sources, thereby ensuring broad temporal, spatial, and authoritative coverage. To avoid duplication bias caused by later citations, we only included the formula records from the earliest extant source of that record. No additional filters were applied for historical era, region, doctrinal school, or contemporary popularity, thus preserving the full diversity of medical practice.

Each formula record is traceable to a classical source text. Notably, significant peaks in the appearance of new formulas and new herbs occurred during the Tang and Song dynasties (periods of great economic and cultural flourishing in ancient China) [5], which likely reflect a high level of clinical practice and medical development during these periods. In subsequent centuries, the number of herbs in the formulas stabilized [5], suggesting that the selection and use of these herbs had been progressively refined through sustained clinical practice. Additionally, TCM formulas and natural-product-derived therapeutic systems continue to be investigated using contemporary pharmacological and mechanistic approaches, providing additional support for their continued clinical and research relevance [6–8].

Standardized TCM entities. Each collected formula record comprises an indication text, which describes the patient’s TCM symptom pattern and differentiated syndromes, and a composition text of the prescribed TCM herbal formula, listing the specific herbs. Three standardized components were extracted from these texts: (1) standardized TCM symptom terms aligned with Clinic Terminology of Traditional Chinese Medical Diagnosis and Treatment [9] and Terminology Specification of Common Clinical Symptoms in TCM (Revised) [10] such as reduced appetite; (2) standardized TCM syndrome terms based on the International Classification of Diseases, 11th Revision (ICD-11) [11] and the Clinic Terminology of Traditional Chinese Medical Diagnosis and Treatment—Part 2: Syndromes/Patterns [12], such as the Syndrome of spleen-Qi deficiency; and (3) standardized TCM herbal compositions based on the NDTCM, such as Ren-Shen (Ginseng Radix et Rhizoma). The detailed procedures for herb, symptom, and syndrome extraction and standardization are described below. An example of an original and processed TCM formula record is shown in Table S2.

Corpus statistics. We identified 1436 unique standardized symptom terms, 149 standardized TCM syndromes, and 2026 unique herbs from the TCM formula records (Figure S2A-C). To minimize the influence of extremely sparse herb entries on learned herb representations, we retained 780 herbs that appeared in at least 25 formula records. These herbs accounted for 98.9% of all herb occurrences and allowed 75,976 of the 84,491 formula records (89.9%) to be represented completely without excluded herbs (Figure S2D, E). A summary of the historical TCM formula corpus and other datasets used in this study is provided in Table S5.

Raw TCM herb extraction and standardization

To extract and standardize the herb names in the TCM formula records’ composition text (Table S2), we implemented a comprehensive pipeline to clean the raw data and establish standardized dictionaries of TCM herbs through the following steps. Notably, the term “herb” initially referred to plant-based Chinese medicines. However, to ensure consistency, the term “herb” is used in a broader sense to encompass animal-based and mineral-based Chinese medicines as well.

Data source. The original herb data were collected from the National Database for Traditional Chinese Medicine (NDTCM) [4]. The dataset includes 8173 herb entries and 84,491 formula records. Each herb entry contains an ID, a standard herb name, aliases (text), a Latin name, and other relevant details. Additionally, 33 herbs (27 contemporary herbs and 6 historical herbs no longer in modern use) were integrated to adapt to formulas from various professional sources. These new herb entries are assigned new IDs in order.

Removing herb entries with incorrect standard names. We identified seven herb entries with misleading standard names. For instance, “白蔻” is commonly known as Amomi Fructus Rotundus but is listed under the entry of Fructus Alpiniae Tonkinensis in the dataset. These entries were removed to ensure consistency among all TCM formula records and clinical data.

Handling herb entries with duplicate standard names. Herb entries’ standard names were reviewed to identify duplicates. If two entries shared the identical standard name, we further compared the Latin names of herb entries, and removed the herb with narrower source species (i.e., the source of the herb is a subset of another). If the Latin names of two herbs did not exhibit a taxonomic containment relationship, both entries were retained with numerical suffixes appended to the end of standard names to distinguish them, while the original standard names were added into aliases.

Constructing alias lists. For each herb entry, we constructed an alias list based on the alias text in original data, by segmenting the original alias text into individual terms based on the designated punctuations. Non-herb terms (e.g., place name “江西” [Jiangxi province], animal name without specific medicinal part “牛” [Ox], and redundant book titles or geographic references “《台湾植物志》”) were removed from the segmented term list.

Adjusting the alias lists. Based on professional knowledge, we manually examined and adjusted the alias list of each herb entry. For example, the alias “益智” was removed from the alias list of the herb entry “龙眼肉” (Longan Arillus) and added to the alias list of herb entry “益智仁” (Alpiniae Oxyphyllae Fructus). Moreover, we also manually identified additional aliases from the frequent terms in the TCM formula records and added them into the alias list.

Establishing the Herb Name-ID Dictionary. All herb terms (including aliases and standard names) were compiled with the IDs of corresponding herb entries to create a Herb Name-ID Dictionary, sorted by the length of herb terms. If a herb term appeared as both standard name and aliases (i.e., the term corresponds to multiple IDs), only the ID of the standard name was reserved.

Establishing the Special Herb Name Dictionary. To deal with the parentheses (e.g., “菴 （艹闾）草” [Herba Artemisiae keiskeanae]) included in herb terms, if the standard name or aliases of a herb entry include these terms, an herb term without parentheses (e.g. “庵蒿” for “菴 （艹闾）草”) was recorded in the Special Herb Name Dictionary, with the ID of the corresponding herb entry recorded accordingly.

Establishing the Descriptive Phrase List. To prevent the interference of the descriptive phrase following the herb term, we segmented the composition text in the formula records into short sentences. We established a Descriptive Phrase List to record the short sentences that simultaneously contain the word “如” (as) and an herb term (e.g., “如枳壳大” [as big as an Aurantii Fructus]) as descriptive phrases.

Establishing the Processing Method Phrase List. Similarly, to prevent the interference introduced by herb processing method phrases, we collected the words representing processing methods of herbs (e.g., “炒” [stir-frying]) from composition texts of formula records and the textbooks, and compiled them into the Processing Method Phrase List. For example, “盐炒” (stir-frying with salt) means that salt (a herb term) was used during processing, instead of serving as a herbal composition in the formula.

Establishing the Herb Pair Dictionary. Since some herb-pairs (including two herbs) were abbreviated as single terms (e.g., “二术” [two kinds of Zhu]), to help identify the individual herbs they contain, we collected common herb-pair terms from the composition texts of formula records, and established a Herb Pair Dictionary to record the two corresponding herb terms (e.g., “苍术” [Atractylodis Rhizoma] and “白术” [Atractylodis Macrocephalae Rhizoma] for “二术”) included in the herb-pairs.

Composition text preprocessing. To facilitate the herb term searching in the composition text, we first replaced herb terms with parentheses by aliases (without parentheses) according to the Special Herb Name Dictionary. Then, we replaced herb-pair terms with corresponding individual herb terms from the Herb Pair Dictionary. Additionally, descriptive phrases were removed according to the Descriptive Phrase List, and common punctuation was replaced with delimiters. Composition texts with missing information were removed.

Searching for herb terms and recording the IDs. We searched for the herb terms (standard names and aliases) in the composition texts of formula records according to the Herb Name-ID Dictionary. Specifically, herb terms with at least two characters were identified and replaced with placeholders. Herb terms with single character were identified if it is not adjacent to placeholders and not followed by processing methods (according to the Processing Method Phrase List). The IDs of identified herb terms were recorded for the composition text of each formula record. Since some herb terms may correspond to multiple IDs (i.e., the herb term appeared as both standard name and alias, or aliases of different herb entries), we established two types of lists to record these IDs for each formula record: (ⅰ) a Determined ID List, which records the IDs of herb terms in the composition text that can be clearly identified with a single ID; and (ⅱ) a set of Candidate ID Lists, where each list corresponds to a herb term with multiple IDs for further determination.

Determining the Candidate IDs. For each candidate ID in a Candidate ID List, we calculated the conditional probabilities of co-occurrence with each determined ID in the same formula record. The confidence score was defined as the sum of these conditional probabilities. Within each Candidate ID List, we selected the ID with the highest confidence score. If multiple IDs shared the highest score, we chose the one with the highest appearance frequency in the Determined ID Lists across all formula records. Finally, the IDs from the Determined ID List were merged with the selected IDs from each Candidate ID List to collectively represent the herbal composition of the formula record.

Frequency-based herb vocabulary filtering. After herb extraction and standardization, 2026 unique herbs were identified across the TCM formula corpus (Figure S2C). For subsequent TCM-ES construction, we retained 780 herbs that occurred in at least 25 formula records. This threshold retained 98.9% of all herb occurrences and allowed 75,976 of the 84,491 formula records (89.9%) to be represented completely using the retained herb vocabulary (Figure S2D, E). Formula records were subsequently subject to the additional model-eligibility criteria described in Note S2.

Raw TCM symptom data preprocessing

The indication texts of TCM formula records (Table S2) are expressed in ancient natural language without fixed aliases or standard symptom terms. To accurately identify symptoms in the indication texts, we designed a method incorporating keyword search and semantic combinations.

Establishing the Standard Symptom Dictionary. Since the indication texts of formula records also include some TCM disease terms (e.g., “梅核气” [plum-stone qi] denotes a sensation of a foreign object in the throat, essentially representing a specific symptom), we referred to the symptom and TCM disease terms from two official terminology documents: the Terminology Specification of Common Clinical Symptoms in TCM (Revised) (TSCCTCM-R) [10] and the Clinic Terminology of Traditional Chinese Medical Diagnosis and Treatment - Part 1: Diseases (CTTCMDT-P1) [9]. Each symptom entry in these two databases contains an original standard symptom name and multiple aliases.

To unify the entries from the two official documents, we first adjusted the standard names of symptom entries from CTTCMDT-P1 according to TSCCTCM-R. Specifically, for each entry in CTTCMDT-P1, if any of its original standard name or aliases appeared in TSCCTCM-R, we adopted that term as the new standard name for the entry. Otherwise, we retained the original standard name in CTTCMDT-P1. By combining the entries from the two documents, we established a Standard Symptom Dictionary. The keys of the Standard Symptom Dictionary are the symptom names in the official documents, and the values are their corresponding standard names. The standard name of each entry was also used as its semantic meaning, which is recorded when the corresponding key is searched in the steps below.

To further merge symptom entries in two official documents with different terms but same semantics, we manually evaluated which symptom entries from CTTCMDT-P1 have semantically corresponding entries in TSCCTCM-R, based on the explanatory content in both documents and domain expertise. Each matched entry was then merged with its corresponding entry from TSCCTCM-R. For example, the symptom “久泻” (chronic diarrhoea) from CTTCMDT-P1 could be mapped to the symptom “久泄不止” (chronic diarrhoea) from TSCCTCM-R.

Performing NLP word segmentation to identify frequent symptom terms in the text. We segmented the indication texts of TCM formula records into words using the Accurate Mode of the Jieba package [13] on the indication texts. Based on the word segmentation, we calculated word frequency and manually identified symptom-related terms from high-frequency words.

Creating the Symptom-related Keyword Dictionary and matching semantic meanings. Due to the diversity of expression in ancient Chinese, only a small part of the symptoms in the text can be directly found by standard symptom names. To deal with the different expressions, we established the Symptom-related Keyword Dictionary by aggregating high-frequency symptom-related words (symptoms, body parts, feelings, etc.). The keys of the dictionary are the symptom related terms, and the values are their corresponding semantic meanings. For instance, “心下” (below the heart, a key in the dictionary) was mapped to the semantic meaning “epigastrium” (a value in the dictionary). After processing all indication texts, the Symptom-related Keyword Dictionary was arranged in descending order of the length of keys.

Establishing semantic meaning combination rules to identify additional symptom terms. We further identified symptom terms formed by the combination of two terms, by combining the semantic meaning of two terms in the Symptom-related Keyword Dictionary. For example, “头” [head] as a body part could be combined with “痛” [pain] to form “headache”.

Segmenting indication text into short sentences to facilitate symptom searching. For each indication text in the formula records, we replaced common punctuations with the delimiter, except for parentheses, quotation marks, slight-pause marks and other punctuations that do not indicate semantic breaks. Each text was then divided into multiple short sentences according to the delimiter.

Conducting a search without replacement. We first searched for the standard symptoms in the Standard Symptom Dictionary. For each short sentence, we searched the symptom terms from the Standard Symptom Dictionary and recorded their corresponding semantic meanings, along with their indices (i.e., the positions) within the short sentence. We searched the symptom terms in descending order of the term while preserving the original context.

Conducting the second search with replacement. We then searched for those frequent symptom terms in the Symptom-related Keyword Dictionary. Keys were sequentially searched in descending order of the word length in each short sentence, with their corresponding semantic meanings recorded along with their indices. Once a key was identified, it was replaced in the sentence with an equally long meaningless placeholder to maintain index consistency.

Identifying symptoms based on semantics or combinations. For each short sentence, all possible combinations of identified semantic meanings were attempted, followed by manual examination, to identify symptoms according to the established rules for combining semantics (e.g., the combination of “口” [mouth] and “生疮” [to develop sores] forms the term “口内生疮” [oral ulcer]).

Integrating symptoms across short sentences. Symptoms found in each short sentence via semantic combinations were standardized using the Standard Symptom Dictionary and the Merged Symptom Dictionary. The found symptoms were first mapped to standard names by searching their semantic meanings in the Standard Symptom Dictionary. The union of symptoms from all short sentences represented the standardized symptoms of the formula text.

Ultimately, these steps collectively processed more than 84% of characters of all indication texts, ensuring consistent symptom representation. This pipeline yielded 1436 unique standardized symptom terms across the TCM formula corpus (Figure S2A). The subsequent semantic encoding of these symptom terms for model input is described in Note S2.

Raw TCM syndrome data preprocessing

TCM encompasses multiple systems of syndrome differentiation. In this study, we focused on the organ-based differentiation system described above, in which each syndrome is characterized by an affected organ and an underlying pathogenesis. Based on the Syndrome Element Differentiation (SED) [14], we included 13 standard syndrome organs or locations, comprising 12 organs and “Exterior”. The corresponding organ and pathogenesis categories are summarized in Table S1.

It is important to note that the concept of “organ” in TCM does not directly correspond to anatomical organs in modern medicine. For instance, the TCM “Heart” (心) often refers not only to the physical heart but also to the brain and nervous system in many contexts. According to the SED, we collected 12 syndrome organs. Additionally, we also included the term “Exterior” (表) from SED. Although “Exterior” does not strictly refer to a specific organ, it indicates that the disorder is caused by external pathogenic factors. Collectively, we included 13 standard syndrome organs in this study.

We collected the syndromes associated with the organ-based syndrome differentiation system from the International Classification of Diseases 11th Revision (ICD-11) [11] and the Clinic Terminology of Traditional Chinese Medical Diagnosis and Treatment - Part 2: Syndromes/Patterns (CTTCMDT-P2) [12]. The syndrome information in the TCM formula data were processed as follows:

Collecting the organ-based syndromes from ICD-11. The ICD-11 contains 104 syndromes. We excluded the syndromes without clear pathogenesis (e.g., “Heart and spleen systems deficiency pattern”), those incorporating additional information such as symptoms (e.g., “Spleen deficiency with food retention pattern”), and those involving multiple organs or pathogenesis (e.g., “Spleen and kidney yang deficiency pattern”). After filtering, we collected 76 syndromes, associated with the 13 organs collected from SED and 30 pathogenesis terms identified from ICD-11.

Collecting the organ-based syndromes from CTTCMDT-P2. From the CTTCMDT-P2, we collected syndromes containing a single organ term and a single pathogenesis term (e.g., “Syndrome/pattern of qi stagnation in the heart meridian”), resulting in 81 syndromes. Combining these with the syndromes from ICD-11, we obtained a total of 157 syndromes involving 13 organs and 42 distinct pathogenesis terms.

Establishing Syndrome-related Keyword Dictionary and syndrome combination rules. We established a Syndrome-related Keyword Dictionary for searching the syndrome terms in the TCM formula record indication text. The Syndrome-related Keyword Dictionary includes three types of keys:

Syndrome keywords. In addition to the 157 syndromes identified (i.e., those associated with single organs and pathogenesis), we also included syndromes from ICD-11 that involved multiple organs or pathogenesis (e.g., “心肝血虚证” [Heart and liver blood deficiency pattern]) as keywords. These syndromes were considered as combinations of multiple syndromes from the 157 syndromes. Additionally, we incorporated 110 unofficial syndrome terms that indicate organs and pathogenesis and frequently appear in formula texts as keywords. For instance, “木火刑金” is an unofficial term indicating liver fire affecting the lung, which was mapped to “syndrome/pattern of liver heat” and “syndrome/pattern of lung qi counterflow”. In this dictionary, each syndrome keyword corresponds to either a single standard syndrome or a combination of standard syndromes (dictionary value), which is regarded as its semantic meaning.

Organ keywords. This type of keywords included the 13 standard organ terms, as well as their non-standard expressions that frequently appear in the indication texts of formula records (e.g., “君火,” referring to the Heart in TCM). Each organ keyword corresponds to a standard organ term (dictionary value), which is regarded as its semantic meaning.

Pathogenesis keywords. This type of keywords included the 42 standard pathogenesis (from ICD-11 and CTTCMDT-P2) terms and their non-standard expressions that frequently appear in the indication texts of TCM formula records (e.g., “血不散” [the blood is not dispersed] is a non-standard expression of blood stasis). Each pathogenesis keyword corresponds to a standard pathogenesis term (dictionary value), which is regarded as its semantic meaning.

Based on the Syndrome-related Keyword Dictionary, we established combination rules similar to the method described above, to identify syndromes by combining the semantic meanings of organ keywords and pathogenesis keywords.

Excluding symptom terms. Before searching for syndrome terms in the text, symptom terms in the TSCCTCM-R were excluded to avoid interference. The symptom terms were removed from the indication texts in descending order of term length and replaced with delimiters to maintain structural clarity.

Searching for syndrome keywords. Based on the indication texts with symptom terms removed, we searched for the syndrome keywords in the Syndrome-related Keyword Dictionary in descending order of term length. The identified syndrome keywords and their semantic meanings were recorded. The identified syndrome keywords were then replaced with delimiters to maintain structural clarity of the text.

Searching for organ and pathogenesis keywords. After syndrome keywords searching, the remaining text was segmented into short sentences according to punctuations and delimiters added in the last step, except for parentheses, quotation marks, slight-pause marks and other punctuations that do not indicate semantic breaks. Within each short sentence, organ and pathogenesis keywords were searched in descending order of length. The semantic meanings of the identified keywords were recorded.

Combining organ keywords and pathogenesis keywords to identify syndromes. The organ and pathogenesis keywords identified within the same short sentence were combined to generate syndrome terms based on the predefined combination rules. For example, in the short sentence “痰火蕴结胃肠” (phlegm and fire obstructing the stomach and intestines), the pathogenesis keyword “痰火” (phlegm and fire) and the organ keyword “胃肠” (stomach and intestines) were identified. These keywords correspond to the standard pathogenesis terms “phlegm” and “heat,” and the standard organ terms “stomach” and “large intestine,” respectively. The two terms were combined to identify three syndromes “Stomach heat pattern” (from ICD-11), “Syndrome/pattern of phlegm and dampness aggregating in the stomach” (from CTTCMDT-P2), and “Large intestine excess heat pattern” (from ICD-11) from this short sentence.

Aggregating the searching results. We integrated the syndromes identified directly through searching syndrome keywords, as well as those derived from the combination of organ and pathogenesis keywords across the indication texts across all formula records. The final results comprised 149 of the 157 syndromes, with 75 sourced from ICD-11 and 71 from CTTCMDT-P2.

Importantly, these syndrome annotations were retained for post-training characterization and evaluation of syndrome-associated organization and were not used as inputs or training targets for the core TCM-ES model.

## Note S2 Construction and characterization of TCM-ES

### Construction of the TCM-ES model and co-occurrence baselines

The core TCM-ES model was trained on matched symptom patterns and prescribed formulas from the standardized historical TCM formula records described in Note S1. The model comprised separate symptom-pattern and formula encoders together with corresponding decoders, incorporating Transformer-based self-attention and cross-attention to model within- and cross-modality relationships (Figure S3). The encoders generated 256-dimensional bottleneck embeddings of symptom patterns and formulas, which were aligned through a combination of reconstruction/prediction and contrastive-learning objectives. Only symptom patterns and formulas were used for core model training. Differentiated syndrome labels, herb-property annotations, clinical outcomes, and biomedical information were not provided as training inputs or targets. After training, the encoder-derived bottleneck embeddings defined the TCM embedding space (TCM-ES), into which individual TCM symptoms, herbs, symptom patterns, and formulas could subsequently be represented. Detailed model architecture, input representations, embedding generation, and training procedures are described as follows.

Model architecture

The proposed model is built upon an autoencoder architecture with multi-head attention blocks (basic block of Transformer [15]). The model integrates two types of input: symptom patterns and TCM formulas, and is trained to capture their interrelationships through self-attention and cross-attention mechanisms [15] (Figure S3, Step 1). The attention mechanisms make the model particularly effective in capturing dependencies in sequential data (e.g., the symptom pattern of a patient or the herbal compositions of a formula) and the correspondence between two inputs. The model consists of the following components implemented using PyTorch.

Input encoding layers: The input encoding layers project inputs into a uniform size suitable for the attention layer processing. The model uses two separate input encoding layers for the two encoders (Figure S3, Encoder 1 and 2): one for symptom pattern input and one for formula input. The symptom pattern encoding layer transforms the input symptom sequence (i.e., symptom pattern) into a sequence of 256-dimensional vectors using a fully connected linear layer. Similarly, the formula encoding layer converts the input herb sequence (i.e., formula) into a sequence of 256-dimensional vectors using a fully connected layer.

Self-attention encoders: The model contains two self-attention encoders: the symptom pattern self-attention encoder (Encoder 1) and the formula self-attention encoder (Encoder 2). Encoder 1 processes the symptom pattern input sequence (converted to 256-dimensional vectors through input encoding layer) through four self-attention blocks, with each block utilizing an 8-head attention mechanism, followed by a feed-forward linear layer, a ReLU activation function, a layer normalization, and a dropout for regularization. Encoder 2 similarly processes the formula input sequence using four 8-head self-attention blocks, with the same architecture as Encoder 1. This design allows the model to learn internal dependencies within symptom patterns and formulas, capturing relationships among symptoms and among herbs, respectively.

Cross-attention decoders: The model includes two cross-attention decoders: the symptom pattern decoder (Decoder 1) and the formula decoder (Decoder 2). Decoder 1 integrates the outputs from both the symptom pattern and formula encoders via cross-attention mechanisms and is trained to reconstruct the input symptom patterns or predict the symptom pattern conditioned on the input formula (if the symptom pattern input is masked). Decoder 2 similarly integrated the encoder outputs to reconstruct the input formula or predict the formula conditioned on the input symptom pattern (if formula input is masked). Both decoders consist of four cross-attention blocks, each with eight attention heads. These cross-attention blocks model context-dependent cross-modal relationships between symptom and herb representations. Notably, cross-attention weights were interpreted as context-dependent model weights rather than as direct measures of herb–symptom therapeutic strength or causal importance. Because the two reconstruction directions are conditioned on different input contexts, the corresponding cross-attention patterns are not expected to be symmetric. Their predictive relevance to cross-modal reconstruction was evaluated separately by token-removal perturbation analyses (Note S3; Figure S8).

Output layers: The model utilizes two output layers for the two decoders, respectively. The symptom pattern output layer (output of Decoder 1) processes the output vectors of the last attention block of Decoder 1 through a single fully-connected linear layer with a SoftMax activation function, mapping the representation to a sequence of 1439-dimensional vectors (each represents the predicted probability of one of the 1436 standardized symptoms, one padding token, one start token, and one end token). Similarly, the formula output layer (output of Decoder 2) processes the output vectors of the last attention block of Decoder 2 to map the outputs to a sequence of 783-dimensional (780 standardized herbs, one padding token, one start token, and one end token) vectors.

In summary, in the forward pass, the input symptom pattern and formula sequences are reshaped using their respective encoding layers, then processed separately through the two encoders. The outputs of two encoders are aggregated and passed through the two decoders. The outputs of decoders are then transformed through the output layers to generate the sequence of reconstructions or predictions for either symptom pattern or formula.

Input and output representations

Symptom pattern input: As described in the main text, each symptom in the symptom pattern is encoded into a 512-dimensional semantic vector using the pre-trained Google Universal Sentence Encoder (GUSE) model [16]. The maximum number of input symptoms is limited to 40 tokens (i.e., up to 40 symptoms for a symptom pattern or patient condition). If the number of input symptoms is fewer than 40, the remaining positions of the sequence are padded with the semantic vector of the padding token (“<P>”). The symptom input sequence begins with the semantic vector of the start token (“<S>”), which indicates the start of the sequence, followed by the symptoms in the symptom pattern, and ends with paddings. Therefore, the total length of the symptom pattern input sequence is 41 tokens.

Formula (herb) input: Unlike symptom pattern inputs, the herbs in the formula are not encoded into semantic vectors. The formula input sequence is represented as a sequence of 783-dimensional one-hot vectors, which correspond to 780 distinct herbs, one start token (“<S>”), one end token (“<E>”), and one padding token (“<P>”). The maximum number of input herbs was constrained to 30 tokens (i.e., up to 30 herbs in a formula). If the input formula contains fewer than 30 herbs, the remaining positions are padded with the one-hot encoding of the padding token. Similarly, the one-hot vector of the start token is appended prior to the formula input sequence, followed by paddings. Thus, the total length of the formula input sequence is 31 tokens.

Symptom output: The output of the symptom pattern decoder (Decoder 1) is a sequence of 1439-dimensional (1436 distinct symptom terms, one start token, one end token, and one padding token) one-hot vectors indicating the reconstructed/predicted symptom pattern. During training, the target output sequence (the ground truth) begins with the real symptoms in the symptom pattern, followed by a vector representing the end token, which marks the end of the non-padding outputs, and ends with paddings. The total length of the symptom pattern target output is 41 tokens.

Herb output: Similarly, the output of formula decoder (Decoder 2) is a sequence of 783-dimensional one-hot vectors. During training, the target output sequence begins with the real herbs in the formula, followed by the vector representing the end token, and ends with paddings. The total length of the herb target output sequence is 31 tokens.

Input masks: During the training processes, for each matched symptom-pattern–formula pair, one of the two inputs is typically masked using an upper-triangular Look-Ahead Mask [15] to prevent the model from "seeing" future prediction steps in sequence generation tasks. For example, when a symptom pattern is input to the model to predict the formula during training, the formula input is masked. This mask ensures that each position in the sequence attends only to preceding positions (i.e., previously predicted symptoms or herbs). Additionally, when calculating the self-attention and cross-attention, padding tokens are masked using a key padding mask. This mask prevents the model from assigning attention to meaningless tokens during training.

TCM-ES embedding generation

Since the encoder (Encoder 1 or 2) processes the input sequence through multiple self-attention blocks, the final output of the encoder is a sequence of contextualized vectors of each input token enriched with information from other tokens in the sequence. By applying mean-pooling to this sequence of vectors, the model generates a single vector aggregating the information of the entire sequence. This 256-dimensional embedding vector serves as a holistic representation of the input sequence (Symptom pattern embedding and Formula embedding in Figure S3, Step 1).

All downstream TCM-ES analyses used embeddings generated from the corresponding unmasked input modality. For example, when the formula input was masked for formula prediction, only the embedding generated from the complete, unmasked symptom-pattern input was used as the symptom-pattern embedding.

Training procedure and loss function

To ensure data reliability and encourage the model to learn the interrelationships within symptom patterns and formulas comprising multiple entities, we curated 84,491 historical TCM formula records. Of these, 75,976 were fully covered by the retained 780-herb vocabulary. Among these, 64,945 had complete symptom and herbal composition information. We then selected 26,339 model-eligible records containing at least three symptoms and three herbs. These records were randomly split into training (60%), validation (20%), and test (20%) sets.

Notably, the primary model used this record-level random split. Potential cross-split similarity among formula records was subsequently assessed explicitly, and a more conservative non-leakage split was constructed by assigning formula-similar records to the same data set (i.e., training, validation, or test set). TCM-ES was retrained from random initialization under this stricter split, as described in Note S3.

For the training set, given that the orders of symptoms or herbs within the sequences are meaningless, we implemented order-shuffling augmentation to eliminate order biases during model training. Specifically, we randomly shuffled the sequence order of each symptom pattern and formula *n* times, where *n* is the minimum number of the symptom sequence length and the herb sequence length. All shuffled versions were incorporated into the training data. This approach expands the training set and suppresses the model's reliance on token order. The length-dependent augmentation was used to provide broader positional coverage for longer sequences, which have more possible token orderings.

Importantly, core TCM-ES training used only matched symptom patterns and prescribed formulas. Differentiated syndrome labels, herb-property annotations, clinical outcomes, and biomedical information were not used as model inputs or training targets.

During each training epoch, the symptom patterns and formulas were fed into the model's two encoders, respectively. Each matched symptom-pattern–formula pair (the symptom pattern and formula from the same formula record) was utilized twice as follows:

First Pass: The herb input was masked using a Look-Ahead Mask, while the symptom input remained unmasked. The model was trained to reconstruct the symptom pattern and predict the formula based on the input symptom pattern. During this pass, we extracted the embedding of the symptom pattern between the Encoder 1 and Decoder 1.

Second Pass: The herb input was unmasked, while the symptom input was masked. The model was trained to reconstruct the formula and predict the symptom pattern. We extracted the embedding of the formula between the Encoder 2 and Decoder 2.

The model was optimized to minimize the distance between matched symptom patterns and formulas while enforcing spatial separation of unmatched pairs through contrastive learning [17]. The loss function comprised two components: reconstruction loss and contrastive loss:

$\text{Loss}_{\text{total}}\text{=}\text{Loss}_{\text{r}}\text{+}\text{β}\text{×}\text{Loss}_{\text{c}}$,

where *β* was the trade-off parameter between two components of the loss. The reconstruction loss *Loss*_r_ was calculated as the average cross-entropy loss of all tokens in the output sequences of two decoders. The contrastive loss adopted from previous work [18] was defined as:

$\text{Loss}_{\text{c}}\text{=}\frac{\text{1}}{\text{N}}\sum_{\text{i}\text{,}\text{j}} \text{[}\left( \text{1-}\text{y}_{\text{ij}} \right)\text{∙}{\text{d}\left( \text{e}_{\text{S}}^{\text{i}}\text{,}\text{e}_{\text{H}}^{\text{j}} \right)}^{\text{2}}\text{+}\text{y}_{\text{ij}}\text{​⋅}{\text{max}\left( \text{0,margin-}\text{d}\left( \text{e}_{\text{S}}^{\text{i}}\text{,}\text{e}_{\text{H}}^{\text{j}} \right) \right)}^{\text{2}}\text{]}$.

*Loss*_c_ was optimized to embed matched symptom-pattern–formula pairs closer together in the embedding space, while pushing unmatched pairs farther apart. Specifically, given two embedding vectors $\text{e}_{\text{S}}^{\text{i}}$ and $\text{e}_{\text{H}}^{\text{j}}\text{ }$(the embeddings of a symptom pattern and a formula, respectively), the contrastive loss was computed based on the Euclidean distance between the embeddings, where *y_ij_* ∈ {0,1} represents the label of symptom-pattern–formula pair, with *y_ij_* = 0 indicating a matched pair and *y_ij_* = 1 for an unmatched pair. The first term minimizes the Euclidean distances for matched pairs, while the second term encourages unmatched pairs to remain separated by the specified margin. The hyperparameter margin controls the tolerance for unmatched pairs. The contrastive loss was averaged over all pairs in the dataset.

Throughout the training process, we tracked the total loss, reconstruction loss and contrastive loss, to monitor the model's performance and convergence. Validation was conducted in parallel to mitigate the risk of overfitting (Figure S4). The checkpoint with the minimum observed total validation loss during training was selected for subsequent analyses.

Embedding dimensionality: We evaluated models with bottleneck dimensions ranging from 8 to 1024 and used 256 dimensions for the primary TCM-ES model. The corresponding dimensionality analysis and sensitivity results are reported in Model training diagnostics and basic properties of TCM-ES.

Mapping TCM entities into the TCM-ES

After core model training, individual symptoms and herbs, as well as complete symptom patterns and formulas, could be represented in the fixed TCM-ES using the trained encoders (Figure S3, Step 2). Individual standardized symptom terms were represented using their GUSE semantic vectors and passed through Encoder 1 to generate symptom embeddings. Complete symptom patterns, including those from external or clinical datasets after standardization, were represented using the same encoder. Individual retained herbs and complete formulas composed of the 780-herb model vocabulary were represented using Encoder 2. Given that the 780 herbs covered the vast majority of clinical formulas, most clinical formulas could be embedded into the TCM-ES directly using Encoder 2. These procedures map TCM entities using the already trained core model and do not modify the learned TCM-ES. Mapping of biomedical entities into the fixed TCM-ES is described separately in Note S6.

Co-occurrence baseline models

To construct the co-occurrence baselines, we first represented each formula record as a binary vector over the complete standardized symptom and herb vocabulary. Each row of the resulting record-by-item matrix corresponded to one formula record, and each column corresponded to one symptom or herb; an entry was set to 1 when that symptom or herb occurred in the record and to 0 otherwise. For example, a record containing symptoms *s*_1_ and *s*_2_, and herbs *h*_1_ and *h*_2_ would contain 1s in the four corresponding columns. We then counted how often every pair of items occurred together across the training records. Because symptoms and herbs were included in the same matrix, this produced symptom–symptom, herb–herb, and symptom–herb co-occurrence counts within a single joint co-occurrence matrix. The co-occurrence counts were subsequently transformed to positive pointwise mutual information (PPMI), which gives greater weight to item pairs that co-occur more often than expected from their individual frequencies while setting negative PMI values to zero.

For the co-occurrence SVD baseline, truncated singular-value decomposition (SVD) was applied to the joint PPMI matrix to generate shared low-dimensional embeddings for symptoms and herbs.

For the co-occurrence graph baseline, symptoms and herbs were represented as nodes in a weighted graph with PPMI values as edge weights, and node embeddings were learned using a random-walk-based graph-embedding method.

For both baseline models, a complete symptom-pattern embedding was obtained by pooling the embeddings of its constituent symptoms, whereas a formula embedding was obtained by pooling the embeddings of its constituent herbs. Neither baseline model used syndrome annotations, clinical outcomes, or other downstream labels.

### Performing PCA on the TCM-ES

For each of the 26,339 model-eligible formula records, the trained model generated one 256-dimensional symptom-pattern embedding and one 256-dimensional formula embedding using the procedures described in Construction of the TCM-ES model and co-occurrence baselines. The two embeddings from each record were averaged to obtain a single 256-dimensional record-level embedding. PCA was fitted to the resulting 26,339 × 256 record-embedding matrix to define the principal directions of the TCM-ES.

For comparison, PCA was also performed on the corresponding raw record representations. Each symptom pattern was represented as a 1436-dimensional multi-hot vector and each formula as a 780-dimensional multi-hot vector; these vectors were concatenated to form a 2216-dimensional raw representation for each record. PCA was fitted separately to these raw representations. The corresponding dimensionality comparisons are reported in Figure S9.

### Model training diagnostics and basic properties of TCM-ES

The core TCM-ES was trained using the matched symptom-pattern–formula records and training procedure described in Construction of the TCM-ES model and co-occurrence baselines. Reconstruction, contrastive, and total losses were monitored on the training and validation sets throughout model training (Figure S4). Reconstruction loss decreased and subsequently stabilized, while the contrastive and total validation losses were used together to monitor training progress and select the model checkpoint for subsequent embedding generation. Because dropout and order-shuffling augmentation were applied only during training, the training reconstruction loss remained slightly higher than the corresponding validation loss over part of the training process. For the primary model shown in Figure S4, the selected checkpoint corresponded to epoch 60.

We next examined the sensitivity of model performance to the dimensionality of the bottleneck embedding. Models with embedding dimensions ranging from 8 to 1024 were evaluated using five repeated training runs for each dimension, with the remaining architecture and training settings kept unchanged (Figure S9A). The minimum validation losses decreased with increasing embedding dimension and approached a plateau when the dimension exceeded approximately 250. Based on this analysis, a 256-dimensional bottleneck representation was used for the primary TCM-ES model. This dimensionality was subsequently used to generate the symptom-pattern and formula embeddings described in Construction of the TCM-ES model and co-occurrence baselines.

The basic variance structure of the learned representation was further examined using PCA as described in Performing PCA on the TCM-ES. For each model-eligible formula record, the 256-dimensional symptom-pattern and formula embeddings were averaged to obtain a record-level embedding. PCA of these record embeddings showed that most of the variation was concentrated in a relatively small number of leading principal components (Figure S9B). In comparison, PCA of the corresponding raw 2216-dimensional record representations, constructed by concatenating the 1436-dimensional symptom multi-hot vector and the 780-dimensional herb multi-hot vector, showed a substantially more dispersed variance spectrum (Figure S9C). Together, these results characterize the compact multiaxial organization of the TCM-ES representation. More systematic comparisons of this geometric organization with semantic, null, and co-occurrence-based embeddings are presented in Note S3 and Figures S16-S18.

For several subsequent embedding-distance analyses, Euclidean distances were additionally expressed on a normalized scale. Let *r*_95_ denote the 95th percentile of the L2 norms of the symptom-pattern and formula embeddings in the model training set. A reference diameter was defined as 2*r*_95_, and the normalized distance between two embeddings *z_i_* and *z_j_* was calculated as

$$\text{d}_{\text{norm}}\left( \text{i} , \text{j} \right)\text{=}\frac{\text{∥}\text{z}_{\text{i}}\text{-}\text{z}_{\text{j}}\text{∥}_{\text{2}}}{\text{2}\text{r}_{\text{95}}}\text{.}$$

The distributions of embedding-vector norms and normalized pairwise distances are shown in Figure S10. This normalization provides a common scale for comparing embedding proximity across the corresponding descriptive analyses, with smaller normalized distances indicating greater proximity. The normalized-distance scale is used in the empirical TCM-practice analyses (Figures S24, S25).

Representative held-out test records were also examined to illustrate the sequential formula outputs generated by Decoder 2 from symptom-pattern inputs (Figure S19). The examples showed different degrees of overlap between the generated herbs and the herbal compositions recorded in the corresponding historical formulas, illustrating the decoder behaviour across individual formula records.

Quantitative formula prediction was further evaluated on the held-out internal test set using the frozen symptom-pattern embeddings, as described in Note S4. Both the linear ridge and nonlinear MLP readouts based on TCM-ES embeddings outperformed the raw symptom multi-hot and shuffled-embedding controls across Precision@*k*, Recall@*k*, and F1@*k* (Figure S20). The MLP provided only modest improvement over the ridge readout, indicating that substantial formula-relevant information was already accessible from the frozen symptom-pattern embeddings using a linear downstream model.

### Syndrome-associated latent organization and additional conventional TCM annotations

To further characterize the relationship between the learned latent dimensions and conventional TCM syndrome organization, we analysed 7098 formula records containing complete symptom-pattern, syndrome, and formula annotations. For each record, the symptom-pattern and formula embeddings were averaged to obtain a 256-dimensional record-level representation. For each of the 40 most frequent TCM syndromes, a point-biserial correlation was calculated between the presence of that syndrome across the formula records and the activation of each of the 256 latent dimensions, resulting in a 40 × 256 syndrome–dimension association matrix (Figure S14A, left). Syndrome annotations were not used during model training and were introduced only for this post-training analysis.

The rows and columns of this matrix were ordered using two distinct sources of information. The 256 latent dimensions (columns) were hierarchically clustered according to their point-biserial correlation profiles across the 40 syndromes. In contrast, the 40 syndromes (rows) were ordered according to their similarity in conventional TCM associations with symptoms and herbs. Specifically, symptom–syndrome and syndrome–herb associations were quantified from the 7098 formula records using normalized pointwise mutual information (NPMI), and each syndrome was represented by its combined symptom-and-herb association profile for clustering (Figure S14A, right). The resulting heatmap showed a clear block-wise pattern, in which groups of syndromes with similar symptom–herb association profiles corresponded to groups of latent dimensions with similar syndrome-associated activation profiles (Figure S14A). This correspondence indicates that the learned latent organization recapitulated aspects of the syndrome relationships independently defined from the symptom–syndrome–herb structure of the historical TCM records.

We next examined whether this pattern depended on the use of point-biserial correlation to quantify syndrome–dimension associations. For each latent dimension, the mean activation among symptom patterns annotated with each syndrome was calculated and standardized relative to the mean and standard deviation of that dimension across all evaluated symptom patterns. Using the same syndrome and latent-dimension ordering as in Figure S14A, the resulting standardized mean activation scores reproduced a similar block-wise pattern (Figure S14B). As a further sensitivity analysis, both syndromes and latent dimensions were then hierarchically clustered directly from the standardized activation matrix, without using the TCM symptom–herb association-based syndrome ordering applied in Figure S14A. A similar block-wise organization was again recovered (Figure S14C). Similar patterns were therefore observed across alternative measures of latent activation and alternative procedures for organizing the syndrome–dimension matrix, supporting the robustness of the distributed syndrome-associated organization in TCM-ES.

We further quantified syndrome-associated organization across all 7273 records carrying at least one syndrome annotation. Syndrome-associated neighbourhood consistency was defined as the proportion of the *k* = 20 nearest neighbours sharing at least one syndrome label with each query record, with neighbours identified separately in the full 256-dimensional symptom-pattern and formula embedding spaces using cosine similarity. Complete record-level syndrome-label sets were randomly permuted among records 1000 times to generate null distributions while preserving syndrome prevalence and within-record label co-occurrence. Observed neighbourhood consistency was 0.170 for symptom-pattern embeddings and 0.173 for formula embeddings, compared with permutation means of 0.044 in both spaces (95% permutation intervals, 0.0427-0.0455 and 0.0428-0.0454, respectively; Holm-adjusted *p*_perm_ = 0.002 for both).

As a complementary readout analysis, one-vs-rest logistic-regression classifiers were fitted to the frozen embeddings using the predefined training records and evaluated on held-out test records. Syndrome labels with at least 10 positive and 10 negative training records were retained. 66 labels with both classes represented in the test set were included in the AUROC analysis (*n*_train_ = 2337, *n*_test_ = 837). Macro-AUROC was 0.844 (95% bootstrap CI, 0.818-0.863) for symptom-pattern embeddings and 0.851 (0.830-0.869) for formula embeddings, based on 1000 bootstrap resamples of the test records (Figure S14D).

We next examined whether the organization of formula embeddings was also associated with additional conventional TCM annotations that were not used during model training. Herb natures and channel attributions were defined as described in Note S1 and Table S1. Formula embeddings showed a continuous gradient according to cold–hot scores derived from the conventional natures of their constituent herbs (Figure S15A). Formula embeddings were also examined according to their dominant herb channel, defined as the most frequent conventional channel attribution among the constituent herbs of each formula. Formulas with different dominant channels occupied partially differentiated and continuous regions of TCM-ES (Figure S15B). Together, these analyses show that the learned organization of TCM-ES was associated with multiple conventional TCM annotations beyond the syndrome labels examined in the main analysis.

## Note S3 Structural validation, robustness, and TCM-associated organization

### Statistical structure of TCM-ES beyond simpler baselines

Null-model and hierarchical analysis of embedding geometry

To examine how different levels of statistical structure contributed to the geometry of TCM-ES, we constructed two complementary null models.

In the shuffled-pairing null model, symptom patterns were retained in their original records, whereas formulas were randomly reassigned within the training, validation, and test sets. This preserved the marginal symptom and herb distributions and the original data splits while disrupting the authentic record-level symptom-pattern–formula correspondence. The shuffled-pairing null model was trained using the same architecture and training procedure as the primary TCM-ES model.

In the independent-modality null model, the symptom-pattern and formula branches were trained without cross-modal attention or contrastive loss, retaining within-modality representation learning while removing explicit cross-modal interaction and alignment.

We then asked how much of the pairwise symptom-pattern and formula embedding geometry in held-out test records could be explained by progressively richer statistical structure derived exclusively from the training set. Specifically, for each test record *i*, let *S_i_* and *H_i_* denote its symptom pattern (symptom set) and formula (herb set), and $\text{z}_{\text{i}}^{\text{S}}$ and $\text{z}_{\text{i}}^{\text{H}}\text{ }$the corresponding frozen symptom-pattern and formula embeddings. For a sampled pair of test records *i* and *j*, embedding proximity was defined as

$$\text{y}_{\text{ij}}^{\text{S}}\text{=-}\left\| \text{z}_{\text{i}}^{\text{S}}\text{-}\text{z}_{\text{j}}^{\text{S}} \right\|_{\text{2}}$$

between two symptom-pattern embeddings and

$$\text{y}_{\text{ij}}^{\text{H}}\text{=-}\left\| \text{z}_{\text{i}}^{\text{H}}\text{-}\text{z}_{\text{j}}^{\text{H}} \right\|_{\text{2}}$$

between two formula embeddings. Larger values therefore indicated greater embedding proximity. To calculate the co-occurrence among individual symptoms and herbs in our standardized vocabulary, binary record-by-item matrices were constructed from the main-model training records, from which symptom–symptom, herb–herb, and symptom–herb positive pointwise mutual information (PPMI) matrices were calculated. For two item sets *A* and *B*, set-level co-occurrence was summarized as

$$\text{C}_{\text{PPMI}}\left( \text{A} , \text{B} \right)\text{=}\frac{\text{1}}{\text{∣}\text{A}\text{∣∣}\text{B}\text{∣}}\sum_{\text{a}\text{∈}\text{A}} \sum_{\text{b}\text{∈}\text{B}} \text{PPMI}\left( \text{a} , \text{b} \right)\text{.}$$

This definition was applied to the corresponding symptom–symptom, herb–herb, or symptom–herb PPMI matrix as appropriate.

Five nested factor sets (*M*1*-M*5) were then constructed, progressing from simple within-modality similarity to increasingly higher-order cross-modal structure.

***M*1 measures within-modality overlap and co-occurrence.** For symptom-pattern embedding proximity,

$$\text{M}\text{1}_{\text{S}}\text{=\{}\text{J}\left( \text{S}_{\text{i}}\text{,}\text{S}_{\text{j}} \right)\text{, }\text{C}_{\text{S}}\left( \text{i} , \text{j} \right)\text{\},}$$

whereas for formula embedding proximity,

$${\text{M}\text{1}}_{\text{H}}\text{=\{}\text{J}\left( \text{H}_{\text{i}}\text{,}\text{H}_{\text{j}} \right)\text{, }\text{C}_{\text{H}}\left( \text{i} , \text{j} \right)\text{\}.}$$

Here, *J* denotes Jaccard similarity, and $\text{C}_{\text{S}}\text{ }$and $\text{C}_{\text{H}}\text{ }$denote set-average symptom–symptom and herb–herb PPMI co-occurrence, respectively. *M*1 therefore captured literal overlap and historical co-occurrence within the same modality as the embedding proximity being modelled.

*M*2 additionally considered overlap and co-occurrence in the counterpart modality. M2 added the corresponding overlap and co-occurrence factors from the paired modality:

$${\text{M}\text{2}}_{\text{S}}\text{=}{\text{M}\text{1}}_{\text{S}}\text{+\{}\text{J}\text{(}\text{H}_{\text{i}}\text{,}\text{H}_{\text{j}}\text{), }\text{C}_{\text{H}}\left( \text{i} , \text{j} \right)\text{\},}\text{}$$

${\text{M}\text{2}}_{\text{H}}\text{=}{\text{M}\text{1}}_{\text{H}}\text{+\{}\text{J}\left( \text{S}_{\text{i}}\text{,}\text{S}_{\text{j}} \right)\text{, }\text{C}_{\text{S}}\left( \text{i} , \text{j} \right)\text{\}}$*.*

Thus, *M*2 tested whether proximity in one embedding modality (i.e., symptom pattern) also reflected similarity between the corresponding paired records in the other modality (i.e., formula).

*M*3 added symptom–herb co-occurrence within and across records. For a symptom pattern (symptom set) *S* and a formula (herb set) *H*, symptom–herb co-occurrence was defined as

$$\text{C}_{\text{SH}}\left( \text{S} , \text{H} \right)\text{=}\frac{\text{1}}{\text{∣}\text{S}\text{∣∣}\text{H}\text{∣}}\sum_{\text{s}\text{∈}\text{S}} \sum_{\text{h∈}\text{H}} \text{PPMI}_{\text{SH}}\left( \text{s} , \text{h} \right)\text{.}$$

For each sampled record pair (*i, j*), the matched and cross-record factors were

$$\text{C}_{\text{ij}}^{\text{matched}}\text{=}\frac{\text{C}_{\text{SH}}\left( \text{S}_{\text{i}} , \text{H}_{\text{i}} \right)\text{+}\text{C}_{\text{SH}}\left( \text{S}_{\text{j}} , \text{H}_{\text{j}} \right)}{\text{2}}\text{,}$$

and

$$\text{C}_{\text{ij}}^{\text{cross}}\text{=}\frac{\text{C}_{\text{SH}}\left( \text{S}_{\text{i}} , \text{H}_{\text{j}} \right)\text{+}\text{C}_{\text{SH}}\left( \text{S}_{\text{j}} , \text{H}_{\text{i}} \right)}{\text{2}}\text{.}$$

*M*3 was therefore defined as

$$\text{M}\text{3=}\text{M}\text{2+}\left\{ \text{C}_{\text{ij}}^{\text{matched}} , \text{C}_{\text{ij}}^{\text{cross}} \right\}\text{.}$$

These factors captured direct symptom–herb co-occurrence both within the observed records and across the two records forming each pair.

*M*4 added higher-order compatibility between symptom combinations and herb combinations. First, the 2000 symptom pairs and 2000 herb pairs with the highest training-set PPMI values were retained as high-confidence pairs. Then for each record, the symptom-pattern or formula was represented by the high-confidence pairs it contained. Let $\text{v}_{\text{S}}\left( \text{S} \right)$ and $\text{v}_{\text{H}}\left( \text{H} \right)$ denote the corresponding pair-incidence vectors. For a symptom-pattern (symptom set) *S_i_* in the test set, its symptom-pair neighbourhood profile over the training records was

$$\text{K}_{\text{S}}^{\text{pair}}\left( \text{S}_{\text{i}} \right)\text{=}\left[ \cos\text{(}\text{v}_{\text{S}}\left( \text{S}_{\text{i}} \right)\text{, }\text{v}_{\text{s}}\left( \text{S}_{\text{r}} \right)\text{)} \right]_{\text{r}\text{∈}\text{I}_{\text{train}}}\text{,}$$

and, analogously, for a formula (herb set) $\text{H}_{\text{j}}$ in the test set,

$$\text{K}_{\text{H}}^{\text{pair}}\text{(}\text{H}_{\text{j}}\text{)=}\left[ \cos\text{(}\text{v}_{\text{H}}\left( \text{H}_{\text{j}} \right)\text{, }\text{v}_{\text{H}}\left( \text{H}_{\text{r}} \right)\text{)} \right]_{\text{r}\text{∈}\text{I}_{\text{train}}}\text{.}$$

Pair-level symptom-pattern–formula compatibility was then defined as

$$\text{PairComp}\left( \text{S}_{\text{i}} , \text{H}_{\text{j}} \right)\text{=}\cos\text{[}\text{K}_{\text{S}}^{\text{pair}}\left( \text{S}_{\text{i}} \right)\text{, }\text{K}_{\text{H}}^{\text{pair}}\text{(}\text{H}_{\text{j}}\text{)]}\text{.}$$

For a sampled pair of records $\text{i}\text{ }$and $\text{j}$, the **matched pair-level compatibility** was defined as

$$\text{P}_{\text{ij}}^{\text{matched}}\text{=}\frac{\text{PairComp}\left( \text{S}_{\text{i}} , \text{H}_{\text{i}} \right)\text{+PairComp}\left( \text{S}_{\text{j}} , \text{H}_{\text{j}} \right)}{\text{2}}\text{,}$$

whereas the **cross-record pair-level compatibility** was defined as

$$\text{P}_{\text{ij}}^{\text{cross}}\text{=}\frac{\text{PairComp}\left( \text{S}_{\text{i}} , \text{H}_{\text{j}} \right)\text{+PairComp}\left( \text{S}_{\text{j}} , \text{H}_{\text{i}} \right)}{\text{2}}\text{.}$$

*M*4 was then defined as

$$\text{M}\text{4=}\text{M}\text{3+}\left\{ \text{P}_{\text{ij}}^{\text{matched}} , \text{P}_{\text{ij}}^{\text{cross}} \right\}\text{.}$$

A high pair-level compatibility value therefore indicated that the symptom-pair and herb-pair structures showed similar neighbourhood patterns across the same training records.

*M*5 added whole-record symptom-pattern–formula compatibility. *M*5 extended the same neighbourhood-profile principle from selected high-confidence pairs to the complete symptom and herb sets. For symptom pattern (symptom set) *S_i_* in test set,

$$\text{K}_{\text{S}}^{\text{record}}\left( \text{S}_{\text{i}} \right)\text{=}\left[ \text{J}\left( \text{S}_{\text{i}} , \text{S}_{\text{r}} \right) \right]_{\text{r}\text{∈}\text{I}_{\text{train}}}\text{,}$$

and for a formula (herb set) *H_j_*,

$$\text{K}_{\text{H}}^{\text{record}}\left( \text{H}_{\text{j}} \right)\text{=}\left[ \text{J}\left( \text{H}_{\text{j}} , \text{H}_{\text{r}} \right) \right]_{\text{r}\text{∈}\text{I}_{\text{train}}}\text{.}$$

Whole-record compatibility was defined as

$$\text{RecordComp}\left( \text{S}_{\text{i}} , \text{H}_{\text{j}} \right)\text{=}\cos\text{[}\text{K}_{\text{S}}^{\text{record}}\left( \text{S}_{\text{i}} \right)\text{, }\text{K}_{\text{H}}^{\text{record}}\left( \text{H}_{\text{j}} \right)\text{]}\text{.}$$

For a sampled pair of records *i* and *j*, the **matched whole-record compatibility** was defined as the average compatibility of the two observed symptom-pattern–formula pairings,

$$\text{R}_{\text{ij}}^{\text{matched}}\text{=}\frac{\text{RecordComp}\left( \text{S}_{\text{i}} , \text{H}_{\text{i}} \right)\text{+RecordComp}\left( \text{S}_{\text{j}} , \text{H}_{\text{j}} \right)}{\text{2}}\text{,}$$

whereas the **cross-record compatibility** was defined using the crossed symptom-pattern–formula pairings,

$$\text{R}_{\text{ij}}^{\text{cross}}\text{=}\frac{\text{RecordComp}\left( \text{S}_{\text{i}} , \text{H}_{\text{j}} \right)\text{+RecordComp}\left( \text{S}_{\text{j}} , \text{H}_{\text{i}} \right)}{\text{2}}\text{.}$$

*M*5 was then defined as

$$\text{M}\text{5=}\text{M}\text{4+}\left\{ \text{R}_{\text{ij}}^{\text{matched}} , \text{R}_{\text{ij}}^{\text{cross}} \right\}\text{.}$$

Thus, *M*5 tested whether whole-record symptom-pattern–formula compatibility provided additional explanatory information beyond the factors already included in *M*1*–M*4. A high value indicated that training records resembling a given test symptom pattern also tended to contain formulas resembling the corresponding test formula.

These five nested factor sets were fitted as separate regression models with pairwise embedding proximity as the response.

For the linear analysis, factors and the embedding-proximity were standardized before fitting ordinary least-squares models. Model fit was quantified by in-sample $\text{R}^{\text{2}}$.

For the nonlinear analysis, the same factors were fitted using gradient-boosting regression and evaluated by fivefold record-level cross-validation. Test pairs were formed from held-out records. Pooled predictions across the held-out folds were used to calculate out-of-sample $\text{R}^{\text{2}}$.

The same analysis was performed on the original TCM-ES and both null models. Each analysis was repeated using 20 independently sampled sets of 200,000 unique test-record pairs. Results are reported as the mean and standard deviation across the 20 pair-sampling repeats. The increase in *R*^2^ from *M_k-1_* to *M_k_* was interpreted as the additional variation in embedding proximity accounted for by the newly introduced factors (Figure S16).

*M*1, comprising overlap and within-modality co-occurrence, explained a substantial component of both symptom-pattern and formula embedding geometry in the original TCM-ES and the two null models (Figure S16). Adding information from the counterpart modality in *M*2 further increased the explained variance, indicating that similarity between the corresponding symptom patterns and formulas contributed additional information to embedding geometry.

The clearest differences among the models emerged with *M*3*–M*5. In the original TCM-ES, inclusion of symptom–herb co-occurrence in *M*3 produced a further increase in explained variance, followed by additional increases after incorporating symptom-pair–herb-pair compatibility in *M*4 and whole-record symptom-pattern–formula compatibility in *M*5 (Figure S16). These progressive increments were observed for both symptom-pattern and formula embeddings.

The corresponding *M*3*–M*5 increments were substantially weaker in the two null models. They were particularly limited in the shuffled-pairing null model, in which the authentic symptom-pattern–formula correspondence had been disrupted. The independent-modality null model retained more of this statistical structure than the shuffled-pairing model, but the later increments remained smaller than those of the original TCM-ES.

The same overall pattern was reproduced in both the linear and nonlinear analyses across the 20 independently sampled test-record pair sets (Figure S16).

Comparison of TCM-principle-associated organization with co-occurrence-based embeddings

We next compared TCM-ES with the co-occurrence SVD and graph embeddings described in Note S2, together with the two null models described above and a semantic baseline constructed by averaging the pretrained GUSE embeddings of the symptoms in each record.

PCA was used to compare how variance was distributed across the record-level embeddings of the original TCM-ES, null models, and baseline models. For the original TCM-ES, shuffled-pairing null model, co-occurrence SVD baseline, and co-occurrence graph baseline, PCA was fitted to the averaged record embedding,

$$\text{z}_{\text{i}}^{\text{record}}\text{=}\frac{\text{1}}{\text{2}}\left( \text{z}_{\text{i}}^{\text{S}}\text{+}\text{z}_{\text{i}}^{\text{H}} \right)\text{,}$$

where $\text{z}_{\text{i}}^{\text{S}}$ and $\text{z}_{\text{i}}^{\text{H}}$ denote the symptom-pattern and formula embeddings of record *i*, respectively. For the GUSE semantic baseline, PCA was fitted directly to the record-symptom embeddings obtained from the averaged symptom semantic vectors. Because the symptom-pattern and formula embeddings of the independent-modality null model were not constrained to occupy a shared aligned space, they were not averaged. PCA was fitted independently for each representation, and the proportion of variance explained by each principal component and the cumulative explained variance were calculated. The resulting variance spectra are shown in Figure S17.

The original TCM-ES formed a compact but multiaxial embedding space (Figure S17A). More than 95% of its record-level variance was captured by the first six principal components. Additionally, the variance was distributed across the first six components rather than being dominated by a single direction.

In contrast, the GUSE semantic baseline (Figure S17B) and two null models (Figure S17C, D) showed similarly diffuse spectra, requiring more than 60 components to reach 95% cumulative variance. Thus, the shuffled-pairing and independent-modality null models did not reproduce the compact leading subspace observed in the original TCM-ES.

The two co-occurrence baselines showed different forms of variance organization. In the co-occurrence SVD baseline, nearly 80% of the record-level variance was concentrated in PC1, while each subsequent component explained only a small proportion (Figure S17E). Its low-dimensional structure was therefore mainly produced by one dominant co-occurrence direction. The co-occurrence graph baseline retained a broader variance spectrum, but approximately 27 components were required to reach 95% cumulative variance (Figure S17F). These results distinguish the compact but multiaxial structure of TCM-ES from both the strongly PC1-dominated SVD representation and the more broadly distributed graph representation.

We then examined whether the different representations preserved syndrome-associated organization and whether this organization was concordant across the symptom-pattern and formula modalities. For the original TCM-ES, shuffled-pairing null model, and the two co-occurrence baselines, symptom-pattern and formula embeddings were projected into the same model-specific PCA space and coloured by the corresponding syndrome labels. PCA was fitted independently for each model, so axes were not compared directly across models.

In the original TCM-ES, syndrome-labelled records occupied continuous but partially distinguishable regions in both the symptom-pattern and formula embedding spaces, with broadly corresponding organization across the two modalities (Figure S18A, B). In contrast, the GUSE semantic-only baseline showed greater intermixing of syndrome-labelled records (Figure S18C), indicating that symptom semantics alone did not reproduce the organization observed after TCM-ES training.

The independent-modality null model retained partial syndrome-associated structure within the symptom-pattern and formula embeddings, but the organization of the two modalities showed weaker correspondence (Figure S18D, E). Disrupting the authentic symptom-pattern–formula pairing in the shuffled-pairing null model produced still greater intermixing and reduced cross-modal correspondence (Figure S18F, G).

The co-occurrence baselines also recovered part of the syndrome-associated organization, confirming that co-occurrence contributes to these patterns. However, the SVD baseline exhibited a strongly compressed geometry, consistent with its PC1-dominated variance spectrum, and its symptom-pattern and formula embeddings showed less clearly corresponding syndrome organization (Figure S18H, I). The graph baseline retained broader variation but likewise showed greater syndrome intermixing and weaker correspondence between the two modalities than TCM-ES (Figure S18J, K).

### Robustness to cross-split formula similarity

Assessment of potential cross-split similarity

Each eligible formula record $\text{i}$ was represented by its symptom pattern (symptom set) *S_i_*, formula (herb set) *H_i_*, and normalized formula title *T_i_*. Similarity between two records *i* and *j* was quantified using symptom-set and herb-set Jaccard coefficients:

$$\text{J}_{\text{S}}\left( \text{i} , \text{j} \right)\text{=}\frac{\text{∣}\text{S}_{\text{i}}\text{∩}\text{S}_{\text{j}}\text{∣}}{\text{∣}\text{S}_{\text{i}}\text{∪}\text{S}_{\text{j}}\text{∣}}\text{, }\text{J}_{\text{H}}\left( \text{i} , \text{j} \right)\text{=}\frac{\text{∣}\text{H}_{\text{i}}\text{∩}\text{H}_{\text{j}}\text{∣}}{\text{∣}\text{H}_{\text{i}}\text{∪}\text{H}_{\text{j}}\text{∣}}\text{.}$$

Because records with the same normalized formula title may represent closely related prescriptions even when their recorded compositions are not identical, separate thresholds were applied to same-title and cross-title record pairs:

$$\text{τ}_{\text{ij}}\text{=}\left\{ \begin{matrix} \text{τ}_{\text{same}}\text{,} & \text{T}_{\text{i}}\text{=}\text{T}_{\text{j}}\text{,} \\ \text{τ}_{\text{cross}}\text{,} & \text{T}_{\text{i}}\text{≠}\text{T}_{\text{j}}\text{.} \end{matrix} \right.$$

We first evaluated potential leakage using a task-specific near-duplicate definition that reflects the symptom-to-formula relationship learned by TCM-ES. Under this definition, two records were considered direct near-duplicates only when both their symptom sets and herb sets were highly similar:

$$\text{J}_{\text{S}}\left( \text{i} , \text{j} \right)\text{≥}\text{τ}_{\text{ij}}\text{ and }\text{J}_{\text{H}}\left( \text{i} , \text{j} \right)\text{≥}\text{τ}_{\text{ij}}\text{.}$$

We examined same-title/cross-title threshold pairs of 0.6/0.9, 0.5/0.8, and 0.4/0.7. At the prespecified 0.6/0.9 setting, the task-specific definition identified 277 cross-split pairs involving 525 unique records, corresponding to 1.99% of the 26,339 eligible records (Table S3). Thus, when similarity was defined according to both sides of the symptom-pattern–formula matching task, direct cross-split near-duplicates were relatively uncommon in the original random split.

Because formula records with highly similar herbal compositions can carry different symptom descriptions, we additionally applied a more conservative formula-based definition based on herb-set similarity alone, without requiring symptom-pattern similarity. Two records were considered formula-similar when

$$\text{J}_{\text{H}}\left( \text{i} , \text{j} \right)\text{≥}\text{τ}_{\text{ij}}\text{.}$$

At the same 0.6/0.9 thresholds, this conservative formula-based definition identified 1999 cross-split pairs involving 3113 unique records, representing 9.21% of the original training set, 15.40% of the validation set, and 16.06% of the test set (Table S3).

Construction of the non-leakage split

The conservative herb-only criterion at the 0.6/0.9 same-title/cross-title thresholds was used to construct a group-disjoint non-leakage split (Table S3). An undirected graph was constructed in which formula records were nodes and formula-similar record pairs were connected by edges. Directly or transitively connected records were merged into connected components using union–find, and each component was assigned entirely to the training, validation, or test set at an approximately 6:2:2 record ratio.

The 26,339 model-eligible records formed 23,465 connected components. TCM-ES was then retrained from random initialization using this group-disjoint non-leakage split.

Re-evaluation using the retrained non-leakage model

We next repeated the principal analyses using the retrained model. For the embedding analysis, PCA was fitted exclusively to the non-leakage training records using the averaged symptom-pattern and formula record embeddings. Held-out test symptom-pattern and formula embeddings were then projected into this fixed training-derived PCA space without refitting PCA.

More than 95% of the training-set variance was captured by the first six principal components (Figure S11A). Syndrome-labelled held-out test records retained the corresponding continuous organization in both symptom-pattern and formula embedding spaces (Figure S11B, C). These visual results were also consistent with the syndrome-associated neighbourhood analysis performed using the same non-leakage splits. Held-out internal formula prediction and external TCM-PD evaluation were additionally repeated using the encoder retrained under the group-disjoint non-leakage split (Figure S11D-G).

### Reproducibility across independent training runs

Ten TCM-ES models were independently trained using different training, validation, and test splits and random initializations. For each repeat, the checkpoint with the optimal validation performance was selected, and the same eligible formula records were embedded using the frozen model. For each record, the symptom-pattern and formula embeddings were averaged to obtain the record embedding.

To assess global stability, the record embeddings were L2-normalized, and cosine similarities were calculated for the same 200,000 randomly sampled non-self record pairs in each repeat. Spearman correlations between the corresponding pairwise-similarity vectors were then calculated for all 45 pairs of independently trained models. The correlations were consistently high, with values concentrated around 0.90-0.93 (Figure S12A).

Local stability was assessed from nearest-neighbour consistency. For each record, its 20 nearest neighbours were identified independently in each repeat. For record $\text{i}\text{ }$in repeat models $\text{a}$ and $\text{b}$, neighbourhood agreement was quantified by the Jaccard overlap:

$$\text{J}_{\text{i}}^{\left( \text{a} , \text{b} \right)}\text{=}\frac{\text{∣}\text{N}_{\text{i}}^{\left( \text{a} \right)}\text{∩}\text{N}_{\text{i}}^{\left( \text{b} \right)}\text{∣}}{\text{∣}\text{N}_{\text{i}}^{\left( \text{a} \right)}\text{∪}\text{N}_{\text{i}}^{\left( \text{b} \right)}\text{∣}}\text{,}$$

where $\text{N}_{\text{i}}^{\left( \text{a} \right)}$ denotes the *k*-nearest-neighbour set of record *i* in repeat *a.* Per-record local stability was defined as the mean Jaccard overlap across all 45 repeat-model pairs. For visualization, the resulting local-stability values were mapped onto the PC1-PC3 coordinates of the primary TCM-ES model (Figure S12B). Local neighbourhoods also showed substantial repeat-to-repeat consistency, although stability varied across the embedding space. Records located toward more peripheral regions of the leading PCA space generally showed higher neighbour stability, whereas records in the dense central region showed relatively lower stability (Figure S12B). This pattern indicates that more geometrically differentiated records tend to retain their local neighbourhoods more consistently, while densely overlapping regions permit greater exchange among similarly positioned neighbours across repeated training.

We then examined whether the principal directions were reproducible. PCA was performed independently on the record embedding generated by each repeated model independently. To establish correspondence between independently fitted PCA axes, Pearson correlations were calculated between the PC-score vectors of the main model and those of each repeat model across the identical records, producing a 6 × 6$\text{ }$correlation matrix for each repeat. Because PCA axes are invariant to sign reversal and may exchange order across independently fitted models, repeat-model PCs were aligned to the main-model PCs by solving a one-to-one assignment that maximized the summed absolute PC-score correlations using the Hungarian algorithm. Repeat PCs were reordered according to this assignment, and any matched PC with a negative correlation to the corresponding main-model PC was sign-reversed. These aligned PC directions were used in subsequent repeat-model analyses.

The leading principal directions showed strong one-to-one correspondence across independently trained models, with particularly consistent alignment for PC1-PC3 (Figure S13). These aligned PCs were used in the subsequent analyses of PC-associated biomedical organization (see Note S7).

### Contributions of model components and attention

Ablation study

The contributions of attention, pretrained symptom semantics, and contrastive learning were examined using three controlled ablation models (Figure S5).

The no-attention model removed both self-attention and bidirectional cross-attention while retaining the surrounding residual, normalization, feed-forward, pooling, reconstruction-head, and contrastive components. The no-contrastive model retained the full architecture but set the contrastive-loss weight to zero, leaving reconstruction as the training objective. The shuffled-semantic model retained the full architecture and training objective but randomly reassigned the pretrained symptom semantic vectors among symptom identities. All models were trained using the same non-leakage split described in Robustness to cross-split formula similarity and the same optimization procedures. We compared the full TCM-ES with these three ablation models using the same non-leakage training, validation, and test sets and the same optimization procedures.

Analysis 1: Training-loss analysis. We first compared the full, no-attention, and shuffled-semantic models under the same reconstruction-plus-contrastive objective. For each training seed, the checkpoint with the minimum validation total loss was selected, and the corresponding total, reconstruction, and contrastive losses were recorded. The no-contrastive model was excluded from this comparison because removing the contrastive term changes the definition of the training objective and therefore makes the total and contrastive losses not directly comparable.

Removing attention increased the validation total, reconstruction, and contrastive losses relative to the full model (Figure S6A-C). Shuffling the pretrained symptom semantic vectors produced a smaller change in reconstruction loss but clearer increases in the total and contrastive losses. The effect of shuffled symptom semantics was therefore more apparent in the contrastive component than in reconstruction performance.

Analysis 2: Syndrome-association analysis. We next evaluated embeddings from all four models using syndrome annotations that were not used during training. Syndrome-associated neighbourhood consistency was defined as the proportion of nearest neighbours sharing at least one retained syndrome label with the query record and was evaluated separately in the symptom-pattern, formula, and cross-modal spaces. The directly matched symptom-pattern and formula embeddings from the same record were excluded from the cross-modal analysis. The primary analysis used *k* = 20, with sensitivity analyses at *k* = 5, 10, 20, 50, and 100 (Figure S6D-I).

The three ablations produced distinct changes in syndrome-associated neighbourhood organization. Removing attention reduced neighbourhood consistency in the symptom-pattern, formula, and cross-modal spaces (Figure S6D-F). Shuffling pretrained symptom semantics produced the clearest reduction in the symptom-pattern space, with a more moderate change in cross-modal organization and comparatively preserved formula embedding organization. Removing contrastive loss reduced syndrome-associated neighbourhood consistency in both modality-specific spaces and produced the largest reduction in the cross-modal analysis. These patterns were maintained across neighbourhood sizes from *k* = 5 to *k* = 100 (Figure S6G-I).

Analysis 3: Direct cross-modal retrieval. We further evaluated direct bidirectional retrieval between matched symptom-pattern and formula embeddings without fitting an additional supervised readout. For each held-out query, the candidate pool contained the matched embedding and 49 randomly sampled unmatched embeddings from the opposite modality. The same candidate sets were used across models and training seeds, candidate sampling was repeated 100 times, and retrieval was evaluated using Hit@1, Hit@5, and Hit@10 in both directions (Figure S6J, K).

The full TCM-ES showed the highest retrieval performance in both directions, whereas the shuffled-semantic model remained relatively close to the full model. Removing attention produced a larger reduction in Hit@1, Hit@5, and Hit@10, while removing contrastive loss produced the largest reduction, with retrieval approaching the random-ranking expectation across the evaluated cut-offs (Figure S6J, K). Together with the neighbourhood analyses, these results show complementary contributions of attention, pretrained symptom semantics, and contrastive learning to the organization of TCM-ES, with contrastive learning contributing particularly strongly to cross-modal alignment.

Attention patterns in representative records

We next visualized representative self-attention and cross-attention patterns generated by the trained model. For the classical formula record Si-Jun-Zi-Tang, self-attention weights from Encoder 1 and Encoder 2 showed context-dependent weighting among symptom and herb tokens within each modality, whereas cross-attention weights from Decoder 1 and Decoder 2 showed the weighting of tokens from one modality during reconstruction of the other modality (Figure S7A-D). Attention weights were averaged across attention heads and layers, as described in Note S2.

In Si-Jun-Zi-Tang, Ren-Shen received relatively high attention weights across several symptom–herb and herb–herb relationships (Figure S7A-D). Ren-Shen was also present in Xuan-Fei-San but showed a different attention-weight pattern (Figure S7E-H), illustrating that the learned attention weights depended on the complete formula-record context.

The symptom representations of three example diseases located near one another in TCM-ES also showed different attention-weight distributions (Figure S7I-K), illustrating context-dependent weighting of constituent symptoms across disease-associated symptom patterns.

Attention-guided token-removal perturbation

To examine the relationship between cross-attention ranking and reconstruction performance, cross-attention weights were extracted from the final cross-attention layer of the frozen TCM-ES for symptom-pattern–formula records in the held-out test set. For each input token, an attention score was calculated by averaging its cross-attention weights across attention heads and across valid query tokens in the opposite modality. For formula reconstruction, a higher symptom attention score therefore indicated greater average attention assigned to that symptom by formula tokens. Conversely, for symptom-pattern reconstruction, a higher herb attention score indicated greater average attention assigned to that herb by symptom-pattern tokens (Figure S8).

Within each record, one, two, or three tokens were removed according to the highest attention scores, lowest attention scores, or random selection. The model was rerun without retraining, and reconstruction degradation was calculated as the increase in negative log-likelihood (NLL) relative to the corresponding complete record. Random removal was repeated ten times and averaged within record. Mean reconstruction degradation and bootstrap 95% confidence intervals were calculated across eligible records. High-attention removal was compared with random and low-attention removal using one-sided paired Wilcoxon signed-rank tests with Benjamini–Hochberg correction (Figure S8).

Removing high-attention symptoms produced the largest degradation of formula reconstruction, whereas removal of low-attention symptoms produced the smallest degradation and random removal showed an intermediate effect (Figure S8A). The same ordering was observed in the reverse direction, with high-attention herb removal producing greater degradation of symptom-pattern reconstruction than random or low-attention herb removal (Figure S8B). These patterns were maintained when one, two, or three tokens were removed. Higher cross-attention scores were therefore associated with greater predictive relevance to reconstruction of the opposite modality.

## Note S4 Formula prediction and external benchmark evaluation

### External TCM-PD benchmark data

We used formula records from the public TCM-PD benchmark dataset [19], which was not used for core TCM-ES training and was used only for external formula-prediction evaluation. Symptoms and herbs in TCM-PD were mapped to the standardized symptom and herb vocabularies used by TCM-ES. Records containing unmatched symptoms or herbs were excluded. This mapping retained 25,997 of 28,746 official training records and 4530 of 5019 official test records with complete symptom and herb coverage, corresponding to 30,527 of 33,765 records overall (90.41%). The original TCM-PD training and test designations were retained for subsequent external evaluation (see Formula-prediction analyses for details).

### Formula-prediction analyses

Held-out internal test set formula prediction

The trained TCM-ES symptom-pattern encoder was frozen before downstream evaluation. For each formula record *i* in the held-out test set, the symptom-pattern embedding *z_i_* was used as input to downstream multilabel readouts for prediction of the herbs in the observed formula, represented by a binary multilabel vector *Y_i_*.

For embedding-based readouts, each embedding dimension was standardized using statistics estimated from the fitting records, and an embedding-derived feature vector was constructed as

$$\text{F}_{\text{i}}\text{=}\left[ {\tilde{\text{z}}}_{\text{i}} , {\tilde{\text{z}}}_{\text{i}}^{\text{2}} \right]\text{,}$$

where ${\tilde{\text{z}}}_{\text{i}}$ denotes the standardized embedding and the squared term was applied elementwise. The same feature construction was applied to the TCM-ES readouts and the shuffled-embedding control. Two prediction heads were applied to the TCM-ES feature vector:

(1) The linear ridge readout predicted the observed-herb vector by minimizing

$$\hat{\text{W}}\text{,}\hat{\text{b}}\text{=}\arg\min_{\text{W}\text{,}\text{b}}\sum_{\text{i}\text{∈}\text{D}_{\text{fit}}} \left\| \text{Y}_{\text{i}}\text{-}\left( \text{F}_{\text{i}}\text{W}\text{+}\text{b} \right) \right\|_{\text{2}}^{\text{2}}\text{+}\text{λ}\text{∥}\text{W}\text{∥}_{\text{F}}^{\text{2}}\text{,}$$

with an unregularized intercept. The same ridge penalty (*λ* = 10) was used for all ridge-based models.

(2) The MLP readout received the same $\text{F}_{\text{i}}$ features and comprised two hidden layers of 1024 and 512 units, respectively, each followed by GELU activation and dropout (0.2), and an output layer producing one score for each candidate herb. The MLP was trained using binary cross-entropy with logits and AdamW optimization.

Both models were then evaluated on the held-out test set. Two baseline models were evaluated:

(1) Raw-symptom baseline. The symptom multi-hot ridge baseline used the raw binary symptom indicator vector directly as input to the same multilabel ridge regression, retaining symptom identities without using TCM-ES embeddings.

(2) Shuffled-embedding control. Symptom-pattern embeddings were randomly reassigned across records before the same standardization, feature construction, and ridge fitting were applied. The shuffle was repeated five times and performance was averaged across repetitions.

For each test record, candidate herbs were ranked by their output scores. Formula prediction was quantified by the precision and recall at *k* = 5, 10, and 20, together with F1 at *k* = 20 (Figure S20).

External TCM-PD formula prediction

External formula prediction was evaluated using the public TCM-PD benchmark [19] described above, which was not used for core TCM-ES training. Symptoms and herbs in TCM-PD were mapped to the standardized symptom and herb vocabularies used by TCM-ES. Records containing unmatched symptoms or herbs were excluded. This mapping retained 25,997 of 28,746 official training records and 4530 of 5019 official test records with complete symptom and herb coverage, corresponding to 30,527 of 33,765 records overall (90.41%).

The official TCM-PD train/test split was retained. 10% of the mapped training records were randomly assigned to a validation subset for downstream readout selection, with the remainder used for readout training. The official external test set was not used for readout fitting or model selection. To reduce direct overlap with the corpus used to train TCM-ES, each mapped external test record was compared with the internal TCM-ES training records using symptom-set and herb-set Jaccard similarities. The internal record with the largest mean of the two similarities was treated as the closest match, and an external test record was excluded when both symptom and herb Jaccard similarities were at least 0.8.

For each retained TCM-PD record, the frozen TCM-ES symptom-pattern encoder generated a symptom-pattern embedding *z_i_*. The observed formula herbs were represented by a binary multilabel vector *Y_i_*. Only frozen symptom-pattern embeddings were used as model-derived input features; formula embeddings were not used in this prediction task.

The same embedding-feature construction and linear ridge and MLP readouts were applied to the TCM-PD records. The readouts were fitted using the mapped TCM-PD training records, with the validation subset used for model selection, and were evaluated on the overlap-filtered external test set.

Candidate herbs were ranked by their output scores, and Precision@*k*, Recall@*k*, and F1@*k* were calculated at *k* = 5, 10, and 20. Uncertainty in the TCM-ES readout performance was estimated using 1000 record-level bootstrap resamples of the overlap-filtered external test set, with the fitted model predictions held fixed. Percentile-based 95% confidence intervals were calculated for Precision@*k*, Recall@*k*, and F1@*k*. For comparison, literature-reported TCM-PD results for LinkLDA, Link-PLSA-LDA, PTM, KDHR, TCMPR, and PresRecST [19–24] were compiled from the published benchmark comparison [20]. Because standardized entity mapping and leakage-control filtering were additionally applied to the TCM-ES evaluation subset, these literature-reported values were treated as contextual benchmark references rather than as a strictly matched head-to-head comparison of algorithms.

## Note S5 Clinical datasets and clinical analyses

### Clinical datasets, Chinese Pharmacopoeia data, and preprocessing

Clinical and pharmacopoeia data sources and inclusion criteria

General clinical cases: The general clinical case data were collected in our “National Key Technologies R&D Program during the 10th Five-Year Plan Period (2004BA721A01H10): Research on Clinical Diagnosis and Treatment Experience and Inheritance Methods of Famous TCM Doctors Based on Information Mining Technology”, which comprehensively documented and analysed the clinical expertise of 108 distinguished TCM practitioners across 140 TCM hospitals and clinics in mainland China over a 2-year period. The original dataset contains 18,892 records of TCM clinical cases. These records provide comprehensive clinical information, covering a diverse distribution of age, sex, education levels, occupations, symptoms and diagnosed diseases (Figure S21A-F), offering a broad perspective on general TCM practices. Each case documented: (1) patient’s condition (symptom pattern) in the initial visit; (2) herbal compositions of the prescribed formula; and (3) improved symptoms in the follow-up visit. We first selected cases with complete documentation of initial and follow-up symptom information and without concurrent interventions such as acupuncture, massage, or modern medications, yielding 5095 general TCM clinical cases. For embedding-based analyses, these cases were further restricted to those whose prescribed formulas were fully covered by the retained 780-herb TCM-ES vocabulary, yielding 2490 cases. An example is provided in Table S6.

Long COVID clinical cases: We incorporated the long COVID clinical cases from our previous rehabilitation study [25] encompassing COVID-19 convalescent patients across various medical centres in Hong Kong. The study tracked 150 patients with long COVID symptoms from discharge to 3-6 months post-discharge. The patients received TCM treatments with up to seven follow-up visits per patient. At each visit, patients’ symptom patterns (conditions) and prescribed formulas were documented. Similarly, we selected cases meeting two criteria: (1) formulas exclusively comprising the 780 high-frequency herbs; (2) documentation spanning ≥ 2 consecutive visits with clear symptom patterns. A total of 144 patients contributing 598 recorded visits were included. For embedding-based longitudinal visit-to-visit analyses, only strictly consecutive recorded visits were retained. Visit gaps were not bridged. Improved symptoms between eligible consecutive visits were identified. An example of long COVID cases is provided in Table S7.

COVID-19 clinical cases: The COVID-19 clinical dataset was from our study, involving 12,950 non-hospitalized patients in Hong Kong diagnosed with COVID-19 via PCR/RAT during the Omicron wave (Dec 31, 2021-May 6, 2022) [26], who received online consultation services provided by the Vincent V.C. Woo Chinese Medicine Clinical Research Institute of HKBU. The dataset systematically documented each patient’s timeline from infection onset through initial medical visit to follow-up visit, with severity of 60 typical symptoms quantified via a standardized 0-5 scoring questionnaire applied at both visits. To ensure the safety of online diagnosis, most patients were prescribed standard formulas (five-day regimen), with occasional modifications. The original dataset included nine standard formulas. For the main analyses, we retained patients assessed using the same symptom evaluation system at both the initial and follow-up visits and treated with one of the four principal formulas, each of which had at least 30 patients with complete initial and follow-up symptom assessments. This filtering resulted in 2132 eligible cases. For each case, we extracted: (1) severity scores of the 60 symptoms during both initial and follow-up visits, and (2) the prescribed formula. In addition to symptom assessments and prescribed formulas, follow-up interval, age, and sex were retained for subsequent adjusted analyses (see Clinical analyses). An example of COVID-19 cases is provided in Table S8. The herbal compositions of the standard formulas are listed in Table S9.

Because the clinical analyses required complete follow-up information and, where applicable, complete TCM-ES formula coverage or restriction to groups with sufficient sample size, the analysed cohorts may not fully represent the broader source populations. Some residual selection bias may therefore remain because complete information was unavailable for all source clinical records.

The clinical data and their respective sources included in our work are summarized in Table S5.

Chinese Pharmacopoeia herb–symptom indications: We additionally used herb indications documented in the Chinese Pharmacopoeia (ChP) as an external reference for established TCM herb–symptom relationships. After herb and symptom standardization, the dataset comprised 3366 documented herb–symptom indication pairs involving 405 herbs and 389 symptoms.

Herbs in clinical cases

Identifying candidate herb terms from the text. For general TCM clinical cases and long COVID cases, each visit of patients contains a composition text of prescribed formula (Table S6 and S7). We identified herb terms from the composition texts using the Herb Name-ID Dictionary from Note S1. We recorded the Determined ID List, the Candidate ID Lists, and the residual words for each composition text.

Dealing with uncertain herb names. Since the herb terms in clinical contexts are more standardized than those in ancient texts, we manually reviewed the Candidate IDs and assigned uncertain herb terms to corresponding herb IDs and standard names by checking the contextual information in the original composition text. For example, “山芋” was identified as an uncertain herb term requiring further manual checking. Upon review, the actual herb name in the text was determined to be “山芋肉,” a common modern misspelling of “山萸肉” (Corni Fructus).

Collecting new herb names from remaining text. After identifying herb terms based on the current Herb Name-ID Dictionary, we continued to collect new herb terms (i.e., herb terms not included in the current dictionary) from the composition text after removing all identified herb terms. We manually examined frequently occurring terms, and identified herb terms that were not included in the current dictionary among them. For each newly identified herb term, we incorporated it into the Herb Name-ID Dictionary with a new herb ID and a standard name based on professional expertise.

Using the updated Herb Name-ID Dictionary, herbs could be re-identified following the same method described in Note S1. For embedding-based clinical analyses, records with prescribed formulas containing herbs outside the retained 780-herb model vocabulary were excluded rather than represented using partially covered formulas.

Symptoms in clinical cases

For the COVID-19 cases, symptoms were recorded using a structured questionnaire rather than extracted from free text. Symptom severity was scored on a 0-5 scale at the initial and follow-up visits. For symptom-level analyses, a symptom was classified as alleviated when its score decreased at follow-up and as unalleviated when its score remained unchanged or increased. For patient-level analyses, overall condition improvement was quantified as the reduction in total symptom score between the initial and follow-up visits.

For general clinical cases and long COVID cases, each visit of patients contains a description text (natural language) of the patient’s symptom pattern (condition). For the general TCM clinical cases, the texts in eight sections of their Electronic Health Record (EHR) were regarded as symptom description texts: “主诉” (chief complaint), “望诊” (inspection), “闻诊” (listening and smelling), “问诊” (inquiry), “舌体” (tongue body), “舌苔” (tongue coating), “脉象选择” (pulse types), and “其他脉象” (other pulse characteristics) (Table S6). For long COVID cases, only one section (current symptoms) was regarded as symptom description text (Table S7).

We adopted the Symptom-related Keyword Dictionary and symptom combination rules described in Note S1 to process clinical symptom description texts and identify standard symptom terms. Importantly, to identify improved symptoms during follow-up visits, we focused on two types of words in the text: (ⅰ) the words indicating that symptoms had improved (or disappeared); and (ⅱ) those indicating that symptoms had not improved. To identify these words, we used the GUSE to compare the words in the symptom description text in the follow-up visit with four standard terms that clearly indicate symptom improvement: “减轻” (alleviated), “好转” (improved), “缓解” (relieved), and “消失” (disappeared). To identify whether symptoms followed by these words were actually improved symptoms, prefix words indicating negation were also identified. The detailed steps are outlined as follows:

Incorporating the words indicating symptom changes into the Symptom-related Keyword Dictionary. Specifically, to further identify the alleviated symptoms in the follow-up visits, we established a list of words indicating symptom changes. Following a process similar to that described in Note S1, the symptom description texts were segmented into single words, and word frequencies were calculated. In particular, for the symptom description texts in the follow-up visits, we checked each word’s semantic similarity to the four standard terms indicating symptom alleviation (e.g., “alleviated,” “improved,” “relieved,” “disappeared”), and defined the highest value as its maximum similarity. Words with ≥ 2 occurrences and ≥ 0.65 maximum similarities were manually reviewed and collected. Additionally, we identified prefix words associated with unimproved symptoms, such as “未” (not yet) and “不” (not). These two types of words were added into the Symptom-related Keyword Dictionary, and assigned the semantic meanings (dictionary values) “alleviation” and “no alleviation”, respectively.

Updating the Symptom-related Keyword Dictionary and symptom combination rules. The Symptom-related Keyword Dictionary from Note S1 was applied to search for symptom terms within the symptom description text. After searching, the remaining residual words in the text were collected. The frequency of these residual words was calculated for each section of the clinical data, and high-frequency words were manually reviewed. We incorporated new symptom-related terms into the Keyword Dictionary as new keywords, manually assigned them corresponding semantic meanings, and developed corresponding new symptom combination rules.

Identifying improved symptoms. Based on the updated Keyword Dictionary and semantic combination rules, we repeated the identification of symptoms present in all visits, and improved symptoms in the follow-up visits. Importantly, the Keyword Dictionary and semantic combination rules are iteratively optimized and updated by incorporating new symptom keywords and combination rules. Therefore, we can reidentify symptoms from previously processed data, ensuring adaptability and scalability for future analyses.

The standardized clinical symptom terms were subsequently represented using GUSE for TCM-ES mapping as described in Note S2.

Chinese Pharmacopoeia herb data

We collected 613 herbs and their indication texts from ChP. We utilized the Herb Name-ID Dictionary (see Note S1) to standardize these herb terms. Since each entry in the ChP data contains only one herb term, we directly searched these herb terms in the Herb Name-ID Dictionary, resulting in 541 herb entries mapped to unique standardized herb IDs, of which 405 were represented in the retained 780-herb vocabulary used for analysis.

Chinese Pharmacopoeia symptom data

We identified standard symptom terms in the indication texts of the 613 herbs in ChP using the updated Keyword Dictionary and semantic combination rules, employing the same method as described in Note S1. The processing resulted in 3366 herb–symptom indication pairs linking 405 herbs to 389 symptoms.

Ethical approval

For long COVID cases, the dataset was collected under the approval of corresponding Research Ethics Committee(s) in HK and followed the guidelines in the Declaration of Helsinki and ICH Good Clinical Practice. The study was reviewed and approved by the following research ethics committees: (1) Clinical Research Ethics Panel, Research Ethics Committee, Hong Kong Baptist University (Approval no. REC/19–20/0468); (2) Joint Chinese University of Hong Kong-New Territories East Cluster Clinical Research Ethics Committee, Hospital Authority New Territories East Cluster, Hospital Authority (CREC Ref. no. 2020.288); and (3) Research Ethics Committee (Kowloon Central / Kowloon East), Hospital Authority (Ref.: KC/KE-20–0240/ER-1). The protocol was registered at the ClinicalTrials.gov (NCT04544605) on 3 September 2020 and published with open access. Informed consent was obtained from all participants.

For general TCM cases and COVID-19 cases, all patients provided informed consent, and all personal data were de-identified. As this retrospective study used anonymized data, no ethical approval was sought.

### Concordance with empirical TCM relationships

We next examined whether pairwise relationships in TCM-ES were concordant with empirical relationships observed in clinical datasets and external TCM reference data. For individual symptoms, symptom–symptom embedding distances were compared with symptom co-occurrence in the general TCM clinical cases, long-COVID cases, and disease-associated symptom data. Pairwise co-occurrence was quantified using the Jaccard index. Across these datasets, symptoms with stronger observed co-occurrence tended to have smaller embedding distances (Figure S24A). Similarly, herb–herb embedding distances were compared with herb co-prescription in the general TCM and long-COVID clinical datasets, with more frequently co-prescribed herbs tending to occupy closer positions in TCM-ES (Figure S24B).

Herb–symptom relationships were further compared with indications documented in the Chinese Pharmacopoeia. The standardized dataset comprised 3366 documented herb–symptom indication pairs involving 405 herbs and 389 symptoms, as described in Clinical datasets, Chinese Pharmacopoeia data, and preprocessing. Documented herb–symptom indication pairs showed shorter embedding distances than non-indicated pairs (Figure S24C).

We additionally performed an exploratory retrospective analysis to examine whether the geometry of herb combinations in TCM-ES was associated with corresponding symptom-recovery patterns in the general TCM clinical cases. We first constructed a set of prevalent herb–herb–symptom triplets [*H_A_, H_B_, S*]. Herb pairs were required to have been co-prescribed in at least 500 historical TCM formula records. For each retained herb pair, candidate symptoms were further restricted to those showing close embedding proximity to both constituent herbs, defined as herb–symptom distances within the 5th percentile of the corresponding reference distance distribution. These criteria yielded 349 herb–herb–symptom triplets for analysis. For each triplet, symptom recovery in the general TCM clinical dataset was compared between cases in which the two herbs occurred individually and cases in which they were co-prescribed. Among cases containing symptom *S* at the initial visit, *I_A_* and *I_B_* denote the numbers of cases prescribed *H_A_* without *H_B_* and *H_B_* without *H_A_*, respectively, and *R_A_* and *R_B_* denote the corresponding numbers of cases in which symptom *S* was subsequently documented as alleviated. The pooled recovery rate for individual-herb use was therefore calculated as

$$\text{e}_{\text{individual}}\text{=}\frac{\text{R}_{\text{A}}\text{+}\text{R}_{\text{B}}}{\text{I}_{\text{A}}\text{+}\text{I}_{\text{B}}}\text{.}$$

Similarly, *I_AB_* denotes the number of cases containing symptom *S* in which *H_A_* and *H_B_* were co-prescribed, and *R_AB_* denotes the number of these cases in which symptom *S* was subsequently alleviated. The corresponding recovery rate for herb-pair use was

$$\text{e}_{\text{pair}}\text{=}\frac{\text{R}_{\text{AB}}}{\text{I}_{\text{AB}}}\text{.}$$

The clinical recovery difference was defined as

$$\text{Δ}\text{e}\text{=}\text{e}_{\text{individual}}\text{-}\text{e}_{\text{pair}}\text{,}$$

such that a negative Δ*e* indicated a higher observed recovery rate when the two herbs were co-prescribed than when they occurred individually, whereas a positive $\text{Δ}\text{e}$ indicated the opposite pattern.

We then quantified the corresponding geometric relationship in TCM-ES. For the same triplet, the distance from symptom *S* to the herb-pair embedding *H_AB_* was compared with the mean distance from *S* to the two individual herb embeddings:

$$\text{Δ}\text{d}\text{=}\frac{\text{d}\left( \text{H}_{\text{A}} , \text{S} \right)\text{+}\text{d}\left( \text{H}_{\text{B}} , \text{S} \right)}{\text{2}}\text{-}\text{d}\left( \text{H}_{\text{AB}} , \text{S} \right)\text{.}$$

A positive Δ*d* therefore indicated that the jointly represented herb pair was embedded closer to the symptom than the two individual herbs on average, whereas a negative value indicated that the herb-pair embedding was relatively farther from the symptom. Across the 349 triplets, Δ*d* was negatively associated with Δ*e* (Figure S24D): herb pairs that shifted closer to the symptom in TCM-ES tended to show a larger relative increase in the observed symptom-recovery rate when co-prescribed, whereas pairs showing the opposite geometric shift tended to show the opposite recovery pattern.

As a sensitivity analysis, the symptom and herb co-occurrence analyses were repeated using normalized pointwise mutual information (NPMI) instead of the Jaccard index. The relationships between smaller embedding distances and stronger observed symptom co-occurrence or herb co-prescription were retained across the corresponding datasets (Figure S25A, B). Together, these analyses show that pairwise relationships in TCM-ES were concordant with multiple empirical relationships observed in clinical practice and external TCM reference data (Figure S24, S25).

### Clinical analyses

Formula-to-individual-symptom relationships

We examined whether the spatial relationship between a prescribed formula and individual symptoms at the time of treatment was associated with subsequent symptom changes. For each clinical case, the prescribed formula and relevant symptoms were mapped into the fixed TCM-ES as described in Note S2, and Euclidean distances were calculated between the prescribed-formula embedding and individual symptom embeddings.

For the COVID-19 cases, symptoms whose severity scores decreased from the initial to the follow-up visit were classified as alleviated, whereas symptoms whose scores remained unchanged or increased were classified as unalleviated. Of the 2132 cases in the revised COVID-19 cohort, 1454 contained at least one symptom in both initial and follow-up visits. These 1454 cases were included in the within-case paired comparison. Formula-to-symptom distances were compared between the alleviated and unalleviated symptoms within each case.

For the general TCM clinical cases, the embedding-based analyses used the 2490 cases whose prescribed formulas were completely covered by the retained 780-herb TCM-ES vocabulary. Symptoms documented as alleviated at follow-up were compared with the remaining symptoms recorded at the initial visit. Two cases lacked a remaining comparison symptom, yielding 2488 cases for the within-case paired comparison.

For the long-COVID cases, only strictly consecutive visit pairs were considered and visit gaps were not bridged. Eligible consecutive visit pairs containing at least one symptom documented as improved and at least one other symptom for within-pair comparison were retained, yielding 203 consecutive visit pairs. Improved symptoms were compared with the other symptoms documented at the preceding visit. Group differences were evaluated using paired two-sided *t*-tests.

Bidirectional complete-set retrieval

Using the 2490 vocabulary-covered general TCM clinical cases, we performed two complementary set-to-set retrieval tasks to examine relationships between complete symptom patterns and formulas.

For initial symptom-pattern–to–formula retrieval, the complete symptom-pattern embedding at the initial visit was used to retrieve the formula prescribed in the same case. For formula-to-alleviated-symptom-pattern retrieval, the prescribed-formula embedding was used to retrieve the complete alleviated-symptom pattern documented in the same case.

TCM-ES used encoder-derived complete-set embeddings. For comparison, the co-occurrence SVD and graph baseline models described in Note S2 were evaluated using the same retrieval tasks. For these two baseline models, complete symptom patterns and formulas were represented by pooling the embeddings of their constituent individual symptoms or herbs, respectively.

For both set-level tasks, each retrieval pool contained the matched target together with randomly sampled unmatched candidates. The default analysis used 50 candidates and 100 repeated samplings, with the same candidate pools reused across models. Retrieval performance was summarized using Hit@1, Hit@5, and Hit@10. The standard error across repeated candidate samplings was used to summarize variability in retrieval performance.

Adjusted patient-condition–formula analysis

Overall multivariable-adjusted association. For patient *i*, patient-condition–formula distance was calculated as the Euclidean distance between the embedding of the initial symptom pattern, $\text{z}_{\text{i}}^{\text{s}}$, and the embedding of the prescribed formula, $\text{z}_{\text{i}}^{\text{f}}$:

$$\text{d}_{\text{i}}\text{=}\left\| \text{z}_{\text{i}}^{\text{s}}\text{-}\text{z}_{\text{i}}^{\text{f}} \right\|_{\text{2}}\text{.}$$

Because absolute embedding distances could differ systematically across prescribed formulas, distances were standardized separately within each formula group:

$${\tilde{\text{d}}}_{\text{i}}\text{=}\frac{\text{d}_{\text{i}}\text{-}\text{μ}_{\text{f}\left( \text{i} \right)}}{\text{σ}_{\text{f}\left( \text{i} \right)}}\text{,}$$

where *f*(*i*) denotes the formula prescribed to patient *i*, and *μ* *_f_*_(_*_i_*_)_ and *σ _f_*_(_*_i_*_)_ are the mean and standard deviation of patient–formula distances among patients receiving that formula. Thus, ${\tilde{\text{d}}}_{\text{i}}$ = 0 represents the average distance among patients receiving the same formula, whereas negative and positive values indicate relatively closer and more distant patient-condition–formula embedding relationships, respectively.

The clinical outcome was calculated as total symptom-score reduction from the initial to follow-up visit:

$$\text{Y}_{\text{i}}\text{=}\sum_{\text{s}\text{=1}}^{\text{128}} \left( \text{InitialScore}_{\text{is}}\text{-}\text{FollowupScore}_{\text{is}} \right)\text{,}$$

where larger values indicate greater overall symptom improvement. The association was estimated using ordinary least-squares regression:

$$\begin{aligned} \text{Y}_{\text{i}}\text{= \&}\text{β}_{\text{0}}\text{+}\text{β}_{\text{1}}{\tilde{\text{d}}}_{\text{i}}\text{+}\text{β}_{\text{2}}\text{InitialTotalScore}_{\text{i}}\text{+}\text{β}_{\text{3}}\text{InitialSymptomCount}_{\text{i}} \\ \text{\&+}\text{β}_{\text{4}}\text{FollowupInterval}_{\text{i}}\text{+}\text{β}_{\text{5}}\text{Age}_{\text{i}}\text{+}\text{β}_{\text{6}}\text{Male}_{\text{i}}\text{+}\text{γ}_{\text{f}\left( \text{i} \right)}\text{+}\text{ε}_{\text{i}}\text{,} \end{aligned}$$

where γ*_f_*_(_*_i_*_)_ represents prescribed-formula fixed effects. InitialSymptomCount_i_ was defined as the number of symptoms with positive scores at the initial visit. The coefficient of interest, *β_1_*, represents the adjusted difference in total symptom-score reduction associated with a one-standard-deviation increase in patient–formula distance within the prescribed-formula group. Negative values therefore indicate that greater patient–formula distance is associated with less subsequent symptom improvement.

To obtain a patient-level measure of symptom improvement adjusted for the included clinical, demographic, and prescribed-formula factors, we derived a covariate-adjusted improvement score from the fitted multivariable model. The covariate-adjusted improvement score was separated into the patient-condition–formula distance component,

$${\hat{\text{D}}}_{\text{i}}\text{=}{\hat{\text{β}}}_{\text{1}}{\tilde{\text{d}}}_{\text{i}}\text{,}$$

and the contribution of all remaining model terms,

$${\hat{\text{N}}}_{\text{i}}\text{=}{\hat{\text{Y}}}_{\text{i}}\text{-}{\hat{\text{D}}}_{\text{i}}\text{.}$$

The covariate-adjusted improvement score was then defined as

$$\text{Y}_{\text{i}}^{\text{adj}}\text{=}\text{Y}_{\text{i}}\text{-}{\hat{\text{N}}}_{\text{i}}\text{+}\overset{‾}{\hat{\text{N}}}\text{,}$$

where $\overset{‾}{\hat{\text{N}}}\text{ }$is the mean non-distance component across all patients. This removes the fitted patient-specific contributions attributable to initial symptom burden, follow-up interval, age, sex, and prescribed formula, while retaining the patient–formula distance component and patient-specific residual on the original symptom-score reduction scale.

Stratified and sensitivity analyses. We next decomposed the overall association to examine whether it depended on a particular follow-up period, patient group, or prescribed formula. The adjusted model was repeated across overlapping initial-to-follow-up time windows and within subgroups defined by sex, age, and initial symptom severity. Separate adjusted analyses were also performed for each of the four retained formulas.

### Diagnostic results for clinical analyses

Frequency and co-occurrence diagnostics for clinical symptom ranking

The individual-symptom analyses in the main text showed that prescribed formulas were located closer to symptoms subsequently documented as alleviated than to unalleviated or other symptoms across the three clinical datasets. We further examined, using the general TCM clinical cases, whether this relationship was retained when formula–symptom ranking was compared directly with the co-occurrence SVD and graph embeddings, and when differences in marginal symptom frequency were controlled (Figure S26).

For the 2490 eligible general clinical cases described in Clinical analyses, symptoms present at the initial visit and subsequently documented as alleviated were defined as positive symptoms, whereas the remaining initial-visit symptoms were defined as negative symptoms. Each initial symptom was ranked according to its proximity to the prescribed formula using TCM-ES or the corresponding co-occurrence-based embedding. All positive–negative symptom pairs were then compared within each case. Pairwise correctness was averaged first within each case and subsequently across cases, yielding a case-balanced pairwise AUC. TCM-ES showed the highest overall within-case ranking performance, with both co-occurrence-based embeddings showing lower pairwise AUCs (Figure S26A).

Because symptom frequency could influence both co-occurrence-based embeddings and the probability that a symptom is observed repeatedly in most clinical records, we next matched positive and negative symptoms according to their marginal frequencies in the TCM-ES training data. For each positive symptom within a clinical case, the negative symptom from the same case with the closest log(1 + *f_s_*) was selected, where *f_s_* denotes the training-set frequency of symptom *s*. The analysis was first performed using all matched pairs and was then repeated after progressively restricting the absolute log-frequency difference between matched symptoms to ≤ 1.0, ≤ 0.75, and ≤ 0.5. Ranking accuracy was averaged first within cases and then across cases, and 95% confidence intervals were obtained from 1000 bootstrap resamples of the contributing clinical cases.

TCM-ES maintained a pairwise ranking accuracy of approximately 0.65 across the frequency-matching conditions, whereas both co-occurrence baselines remained lower (Figure S26B). The separation was retained, and became more apparent under the stricter frequency-matching criteria, indicating that the observed difference in formula–symptom ranking was not explained solely by marginal symptom-frequency differences.

We further examined whether the relative performance of TCM-ES varied across individual symptoms with different training-set frequencies. This analysis was restricted to symptoms that occurred as both positive and negative symptoms in at least five clinical cases. For each such symptom, the corresponding clinical cases were ranked according to its relative proximity to the prescribed formula, and an individual-symptom AUC was calculated to quantify whether cases in which that symptom was subsequently alleviated tended to receive higher proximity scores than cases in which it was not alleviated. The difference between the TCM-ES AUC and the corresponding co-occurrence SVD AUC was then calculated for each symptom, and symptoms were grouped into eight bins according to their frequencies in the TCM-ES training data.

TCM-ES and the co-occurrence SVD embedding showed broadly comparable performance for many of the most frequent symptoms, whereas larger positive AUC differences in favour of TCM-ES were observed for many lower- and intermediate-frequency symptoms (Figure S26C). Thus, the relative difference between the two embeddings was not uniform across symptoms and was more apparent where marginal co-occurrence statistics were less abundant.

Sensitivity analyses of the adjusted patient-condition–formula association

In the primary analysis of the 2132 COVID-19 cases, patient-condition–formula distance was calculated between the initial symptom-pattern embedding and the prescribed-formula embedding and standardized within each prescribed-formula group, as described in Clinical analyses. Condition improvement was quantified as the reduction in total symptom score from the initial to follow-up visit. After adjustment for initial total symptom score, initial symptom count, follow-up interval, age, sex, and prescribed-formula fixed effects, greater within-formula patient-condition–formula distance was associated with less subsequent condition improvement (*β* = -0.306 per 1-SD increase in distance, 95% CI: -0.520 to -0.092, *p* = 0.005). We therefore examined whether this association was concentrated within particular follow-up periods, patient subgroups, or prescribed formulas (Figure S27).

The adjusted regression model was first repeated across overlapping windows of the initial-to-follow-up interval. The estimated coefficients were generally negative across the evaluated follow-up windows, although their magnitudes and precision varied with the number and composition of cases contributing to each window (Figure S27A). The direction of these estimates was broadly consistent with the association obtained from all 2132 observed cases.

We next repeated the adjusted analysis within patient subgroups defined by sex, age, and initial symptom severity. The estimated associations were again predominantly in the negative direction across the evaluated subgroups, while effect magnitude and confidence-interval width varied among groups (Figure S27B). This pattern indicates that the overall association was not restricted to a single demographic or baseline-severity subgroup.

Finally, separate adjusted analyses were performed within each of the four principal prescribed-formula groups. The formula-specific estimates showed some heterogeneity: the negative association was clearer for some formulas and weaker or less precisely estimated for others (Figure S27C). Thus, the overall relationship was not attributable solely to comparisons among different prescribed formulas, although its magnitude varied across formula-specific patient groups.

Together, the stratified analyses showed a broadly similar directional pattern across follow-up intervals and patient subgroups, with greater heterogeneity across individual prescribed formulas (Figure S27). These analyses support the robustness of the adjusted retrospective association while also indicating variation in its magnitude and precision across clinical subsets.

## Note S6 Biomedical and drug-related data, and post hoc entity mapping

### Modern biomedical data

The biomedical datasets described below were used only after construction of the core TCM-ES and were not used for its training.

Human diseases and associated symptoms

We incorporated human disease–symptom associations from a previous study [27], in which the dataset was curated from PubMed publications. It contains disease or symptom terms from the MeSH [28] database, including 147,978 associations between 4219 diseases and 322 symptoms. The strength of disease–symptom association was quantified using term frequency-inverse document frequency (TF-IDF) [29]. We further selected 9524 strong disease-symptom associations (co-occurrence ≥ 50 and TF-IDF ≥ 100). Based on these associations, we identified diseases that could be differentiated by symptoms, defined as having three or more associated symptoms or at least one specific symptom (defined as a symptom associated with fewer than 10 diseases). This filtering resulted in a final set of 479 diseases.

Because literature-derived disease–symptom associations may incompletely capture clinically established symptoms, we additionally constructed a complementary symptom dataset using Mayo Clinic information for sensitivity analyses.

Each disease was additionally annotated according to its top-level category or categories under the Diseases [C] categories in the MeSH hierarchical tree [30] (e.g., Infections [C01], Neoplasms [C04], Immune System Diseases [C20]).

The disease-associated symptom terms were subsequently used to generate disease representations in the fixed TCM-ES, as described in Mapping biomedical entities into the fixed TCM-ES.

Herbal compounds and target proteins

Herbal compounds and target proteins associated with the retained herbs were obtained from HIT 2.0 [31]. HIT 2.0 is a comprehensive database of herbal compound–target interactions, developed by extracting compound–target relationships through text mining of literature abstracts, followed by meticulous manual curation. In total, 417 of the 780 high-frequency herbs are found in HIT 2.0, encompassing 911 compounds and 2030 target proteins.

Disease-associated genes

We adopted a disease–gene association dataset from prior research [32], which was aggregated from the Online Mendelian Inheritance in Man (OMIM) [33] and Genome-Wide Association Studies (GWAS) [34]. This dataset includes a total of 299 diseases (from MeSH, each with ≥ 20 associated genes) associated with 3173 unique genes. Among these diseases, 79 diseases overlapping with our 479 diseases (i.e., diseases with associated symptoms) were selected for downstream gene- and target-related analyses.

Human protein interactomes

We employed the human PPI network from a previous study [35] to assess the concordance between TCM-ES spatial relationships and established molecular relationships. The PPI network comprises experimentally validated interactions, including direct binary interactions, complex formations identified via affinity purification coupled with mass spectrometry, kinase-substrate relationships, pivotal interactions within signalling cascades, and essential regulatory mechanisms. The PPI network consists of 18,505 proteins interconnected through 327,924 interactions.

Refining disease-associated symptoms using Mayo Clinic data

In our main analysis, disease–symptom associations were derived from literature-based co-occurrences of disease and symptom terms. However, literature-derived disease-symptom associations carry inherent limitations. Clinically well-established symptoms with strong disease specificity tend to be underrepresented in research publications, due to publication bias favouring novel discoveries over documentation of well-established symptoms.

To address this gap and refine our disease associated symptom data, we referred to the Mayo Clinic [36], a trusted clinical resource that provides curated symptom bulletins for various diseases. We systematically searched the Mayo Clinic website using each disease term from our dataset and extracted the diseases for which symptom bulletins were available. Each bulletin (presented in natural language) was summarized into concise and standardized symptom phrases using GPT-4o with a specifically designed prompt, followed by the filtering steps described below.

The disease associated symptom phrases collected from Mayo Clinic were integrated with the literature-based disease associated symptoms. To ensure semantic consistency and eliminate redundancy, we employed the following two steps to filter the Mayo Clinic symptom phrases:

Identifying complementary symptoms. The symptom phrases obtained from Mayo Clinic were transformed into semantic vectors using the GUSE. For each disease, we computed cosine similarity between the newly collected symptom (from Mayo Clinic) semantic vectors and the pre-existing disease associated symptom (literature-derived) vectors. Only symptom phrases with a cosine similarity $\text{≤0.7}$ were retained, ensuring that the new additions complemented rather than duplicated existing associated symptoms.

Ensuring semantic coherence with TCM symptoms: To ensure that the new symptom phrases are applicable to our model trained on TCM symptoms, the symptom phrases were further filtered based on their similarity to our 1436 TCM symptoms. Specifically, symptom phrases with a cosine similarity ≥ 0.4 to at least one TCM symptom were retained.

### Drug–disease indication and FAERS-derived observational data

Primary drug–disease indication dataset

Drug–disease indication pairs and their associated FAERS-derived Relative Efficacy (RE) scores were obtained from a prior study [37]. The dataset included 402 drug–disease indication pairs with strong literature evidence, involving 238 drugs and 79 diseases. Among these pairs, 204 had available RE scores, each based on at least 10 adverse-event reports. The RE score was derived from spontaneous adverse-event reports in the FDA Adverse Event Reporting System (FAERS) using information on ineffectiveness reports and reporting volume [38]. Because FAERS is an observational spontaneous-reporting system, the RE score was treated in this study as an indirect observational measure and not as a validated quantitative measure of therapeutic efficacy. Detailed construction and analysis of the RE score are described in Mapping biomedical entities into the fixed TCM-ES.

For downstream analyses, we retained drug–disease indication pairs in which (1) the disease was represented among the 479 symptom-defined diseases and (2) the drug satisfied the predefined mapping criteria for the herbal-compound-trained encoder described in Mapping biomedical entities into the fixed TCM-ES. This filtering yielded 125 drug–disease indication pairs involving 94 drugs and 27 diseases, of which 77 pairs had available RE scores.

A summary of the biomedical and drug-related datasets used in this study is provided in Table S5.

Additional drug and reporting datasets for sensitivity analyses

Additional datasets were used to construct and examine the robustness of the exploratory drug–disease analyses. Approved drugs were obtained from DrugBank [39], established drug–disease indication pairs were obtained from MEDI-2 [40], and adverse-event reports were obtained from FAERS through the openFDA API. The openFDA records used in the analysis spanned 2004 through the second quarter of 2024. Drug structural information was retrieved from PubChem [41], and estimated U.S. prescription volumes for selected drugs were obtained from the ClinCalc DrugStats database [42]. Detailed procedures for terminology alignment, construction of the FAERS-derived RE score, and assessment of its underlying reporting-volume assumption are described in Mapping biomedical entities into the fixed TCM-ES. Sensitivity analyses using alternative drug-mapping criteria, refined disease-associated symptoms, and updated openFDA data are reported in Note S9 and Figure S35.

### Mapping biomedical entities into the fixed TCM-ES

After construction of the core TCM-ES, biomedical entities were mapped into the fixed embedding space through their established relationships with TCM entities (Figure S3, Step 3). Biomedical information was not used to train or modify the core TCM-ES.

Mapping human diseases into the TCM-ES

For each of the symptom-defined diseases described in Modern biomedical data, the associated standardized symptom terms were encoded using the GUSE representation described in Note S2. The resulting symptom-pattern embedding was then passed through the fixed Encoder 1 to generate the disease embedding in TCM-ES (Figure S3, Step 3).

Mapping herbal compounds and target proteins into the TCM-ES

Herbal compounds: Herbal compounds were mapped into the fixed TCM-ES using their known source-herb relationships. The mapping objective encouraged compounds to be embedded close to their associated source herbs while remaining separated from randomly selected non-associated herbs. We collected 911 herbal compounds associated with 417 of the 780 retained herbs from the HIT 2.0 database [31]. A pretrained Mol2Vec model [43] was used to encode each compound into a 300-dimensional structural vector from its SMILES representation.

To map these compound Mol2Vec representations into the fixed TCM-ES, we trained a supplementary autoencoder (Autoencoder 3; Figure S3, Step 3) under supervision from the corresponding herb embeddings. This fully connected network comprised a 300-dimensional input layer, three hidden layers of 512, 256, and 512 dimensions, and a 300-dimensional output layer, with the 256-dimensional bottleneck representation serving as the compound's embedding within the TCM-ES. Autoencoder 3 simultaneously optimized: (ⅰ) input reconstruction fidelity and (ⅱ) compound–herb proximity within the TCM-ES. The loss was defined as follows:

$$\text{Loss}_{\text{A3}}\text{=}\text{Los}\text{s}_{\text{MS}\text{E}_{\text{A3}}}\text{+}\text{β}\text{×}\text{Losss}_{\text{contrastiv}\text{e}_{\text{A3}}}\text{,}$$

where $\text{Los}\text{s}_{\text{MS}\text{E}_{\text{A3}}}$ was the mean squared error (MSE) between the input and reconstructed SMILES vectors. The contrastive term was defined analogously to that used for the core TCM-ES model (Note S2), with associated compound–herb pairs treated as positive pairs and randomly sampled compound–herb pairs as negative pairs. A 10-fold cross-validation procedure was used during Autoencoder 3 training.

After training, herbal compounds were mapped into the fixed TCM-ES from their Mol2Vec structural representations using Encoder 3. Autoencoder 3 was trained exclusively on herbal compounds and therefore was not assumed to provide reliable representations for modern drugs outside the chemical domain supported by the herbal-compound training data. The additional criteria used to assess the mapping confidence of modern drugs is reported in Note S9 and Table S10. Training Autoencoder 3 did not update the parameters of the core TCM-ES; the pretrained TCM encoders and existing TCM entity embeddings remained fixed.

Target proteins: Target proteins were mapped into the fixed TCM-ES using their known herb–target relationships. A supplementary autoencoder (Figure S3, Step 3, Encoder and Decoder 4, collectively referred to as Autoencoder 4) was trained to generate target protein embeddings, supervised by the embeddings of corresponding herbs. Unlike the SMILES vector representation of compounds, target proteins were represented as 2030-dimensional one-hot vectors corresponding to the 2030 distinct targets in our dataset. Similarly, Autoencoder 4 was a fully connected network comprising 2030-dimensional input and output layers and three hidden layers (512, 256, and 512 dimensions). A SoftMax layer was appended before the final output to generate the probabilities for target reconstructions. The 256-dimensional bottleneck representation served as the embedding for the input target protein. The training process employed a loss function with two components:

$$\text{Loss}_{\text{A4}}\text{=}\text{Los}\text{s}_{\text{C}\text{E}_{\text{A4}}}\text{+}\text{β}\text{×}\text{Los}\text{s}_{\text{contrastiv}\text{e}_{\text{A4}}}$$

where $\text{Los}\text{s}_{\text{C}\text{E}_{\text{A4}}}$ was the cross-entropy loss between the original one-hot vector and the reconstruction at the output layer. The contrastive term treated known herb–target associations as positive pairs and randomly sampled herb–target combinations as negative pairs, encouraging associated targets and herbs to occupy closer positions in the embedding space. Training Autoencoder 4 did not modify the pretrained core TCM-ES.

Mapping modern drugs and exploratory drug–disease analyses

Modern-drug mapping and mapping-confidence assessment. Modern drugs were mapped into the fixed TCM-ES using the herbal-compound encoder (Figure S3, Step 3; Encoder 3). For each drug, its SMILES representation was converted into a 300-dimensional structural vector using the pretrained Mol2Vec model and then passed through Encoder 3 to generate a 256-dimensional drug embedding. The corresponding Decoder 3 output was used to reconstruct the input structural vector. Because Autoencoder 3 was trained exclusively on herbal compounds, its applicability to modern drugs with chemical structures outside the herbal-compound training domain could not be assumed. We therefore evaluated each drug using both structural reconstruction fidelity and the geometric location of its embedding before including it in downstream analyses.

For the primary drug–disease analysis, two criteria were applied.

(1) The reconstructed Mol2Vec vector generated by Decoder 3 was compared with the drug’s original input vector and the Mol2Vec vectors of the herbal compounds used to train Autoencoder 3 using cosine similarity. The drug satisfied the reconstruction criterion only when its reconstructed vector was most similar to its own original input vector.

(2) The geometric centroid of the core TCM-ES was calculated from the symptom-pattern and formula embeddings of the historical formula records. The distribution of Euclidean distances from these embeddings to the centroid was then calculated, and a drug satisfied the geometric-domain criterion when its embedding lay within twice the 95th-percentile distance from the centroid.

Together, these criteria provided an operational screen for restricting the analysis to modern drugs for which the herbal-compound-trained mapping was considered sufficiently supported. The broader mapping-confidence assessment of approved drugs is described separately in Note S9 and Table S10.

Drug–disease proximity. Diseases were mapped into the fixed TCM-ES as described above, and drugs passing the above screening criteria were mapped using Encoder 3. Euclidean distance between the corresponding embeddings was used to quantify drug–disease proximity. Because different drugs and diseases could have different background distance distributions within the integrated TCM-ES, we additionally calculated a bidirectional *z*-score (BDZ) for each drug *d* and disease *s*. The drug-to-disease *z*-score was defined as

$$\text{z}_{\text{d}\text{→}\text{s}}\text{=}\frac{\text{x}_{\text{ds}}\text{-}\text{μ}_{\text{d}}}{\text{σ}_{\text{d}}}\text{,}$$

where *x_ds_* is the embedding distance between drug *d* and disease *s*, and *μ_d_* and *σ_d_* are the mean and standard deviation of the distances from drug *d* to all evaluated diseases. Conversely, the disease-to-drug *z*-score was defined as

$$\text{z}_{\text{s}\text{→}\text{d}}\text{=}\frac{\text{x}_{\text{ds}}\text{-}\text{μ}_{\text{s}}}{\text{σ}_{\text{s}}}\text{,}$$

where *μ_d_* and *σ_d_* are the mean and standard deviation of the distances from disease *s* to all evaluated drugs. BDZ was then calculated as

$$\text{BDZ}_{\text{ds}}\text{=}\frac{\text{z}_{\text{d}\text{→}\text{s}}\text{+}\text{z}_{\text{s}\text{→}\text{d}}}{\text{2}}\text{.}$$

Lower BDZ values therefore indicate closer mutual drug–disease proximity relative to the respective background distance distributions of both entities. BDZ was used as an embedding-based matching metric and was not interpreted as a direct measure of therapeutic efficacy.

FAERS-derived observational measure. For exploratory comparison with an external observational measure, we used the Relative Efficacy (RE) score [37] available for a subset of the drug–disease indication pairs. The score was calculated from reports in the FDA Adverse Event Reporting System (FAERS) as

$$\text{RE=1-}\frac{\text{n}_{\text{ineffective}}}{\text{n}_{\text{top}}}\text{,}$$

where *n*_ineffective_ denotes the number of reports containing an ineffectiveness-related event for the corresponding drug–disease pair, and *n*_top_ denotes the number of reports for the most frequently reported adverse event, used as an approximate reporting-volume denominator. Larger RE values therefore indicate fewer reported ineffectiveness events relative to this reporting-volume proxy. Because FAERS is a spontaneous adverse-event reporting system rather than a clinical efficacy database, the RE score was treated only as an indirect observational measure and not as a validated quantitative measure of therapeutic efficacy. Its association with drug–disease proximity was therefore examined only as an exploratory, hypothesis-generating analysis.

All modern-drug analyses were performed after construction of the core TCM-ES and did not modify its learned parameters. The broader assessment of modern-drug mapping confidence, the diagnostic assessment of assumptions underlying the FAERS-derived score, and sensitivity analyses using alternative drug-screening criteria, refined disease-associated symptoms, and updated FAERS/openFDA data are reported separately in Note S9.

## Note S7 Biological interpretation and robustness of the integrated TCM-ES

### Related biological functions of the TCM-ES PCs

After PCA was fitted to the record-level embeddings, other entities represented in the same 256-dimensional TCM-ES were projected onto the fixed PCA axes without refitting PCA. These included individual symptoms and herbs, as well as biomedical entities mapped post hoc into the fixed TCM-ES as described in Note S6. For each entity, its coordinate along a principal component was defined by its projection onto the corresponding PCA axis; larger coordinates therefore indicate positions further along the positive direction of that PC. Biomedical entities were not used to fit the PCA axes and were projected only after construction of the core TCM-ES and its principal-component coordinate system.

### GO-based analysis of TCM-ES principal directions

TCM-ES and its principal-component axes were established without using herb–target associations, protein annotations, or Gene Ontology (GO) [44] information. After the embedding space and PCA directions had been fixed, representative herbs with high projections on PC1-PC3 were examined for convergent pharmacological themes reported in previous studies. These observations motivated three data-informed, literature-supported candidate domains: (1) thermogenic and mitochondrial oxidative metabolism for PC1; (2) airway innate-inflammatory response for PC2; and (3) fatty-acid oxidation and cholesterol handling for PC3. For example, experimental studies have linked representative PC1-associated herbs or constituents to thermogenic and energy-metabolic processes [45]; representative PC2-associated herbs to inflammatory, TLR4/NF-κB, and respiratory-inflammatory processes [46,47]; and representative PC3-associated herbs to fatty-acid oxidation and cholesterol metabolism [48,49]. Thus, the candidate modules were data-informed and literature-guided. Their definitions were subsequently fixed before the quantitative repeat-model, permutation, and sensitivity analyses.

Each candidate domain was represented by a fixed set of GO Biological Process anchors and their descendant terms connected through “is_a” or “part_of” relationships (Table S11). The thermogenic and mitochondrial oxidative metabolism module included heat generation (GO:0031649), response to cold (GO:0009409), and aerobic respiration (GO:0009060). The airway innate-inflammatory response module included toll-like receptor 4 signalling pathway (GO:0034142), regulation of type I interferon production (GO:0032479), response to type I interferon (GO:0034340), acute inflammatory response (GO:0002526), regulation of acute inflammatory response (GO:0002673), and regulation of leukocyte degranulation (GO:0043300). The fatty-acid oxidation and cholesterol handling module included fatty acid beta-oxidation (GO:0006635), cholesterol biosynthetic process (GO:0006695), cholesterol transport (GO:0030301), and acylglycerol catabolic process (GO:0046464). Human proteins annotated to at least one anchor or descendant term and represented in the herb–target dataset were assigned to the corresponding module. Proteins associated with identical sets of herbs were collapsed into a single herb-association profile so that duplicated target annotations did not receive disproportionate weight.

Individual-herb embeddings were projected onto the PCA axes of the corresponding model. To establish correspondence between independently fitted PCA axes in each repeat model, Pearson correlations were calculated between the PC-score vectors of the main model and each repeat model across the same formula records. Repeat-model PCs were matched one-to-one to the main-model PCs using the Hungarian algorithm to maximize the summed absolute correlations. Matched PCs with negative correlations were sign-reversed before downstream analyses.

For model *m*, aligned PC *p*, and top-*K* threshold, $\text{T}_{\text{mp}}^{\left( \text{K} \right)}$ denotes the *K* herbs with the highest projections on that axis. For a unique target-protein profile *g*, with associated herb set *H_g_*, PC exposure was defined as

$$\text{E}_{\text{gmp}}^{\left( \text{K} \right)}\text{=}\frac{\text{∣}\text{H}_{\text{g}}\text{∩}\text{T}_{\text{mp}}^{\left( \text{K} \right)}\text{∣}}{\text{∣}\text{H}_{\text{g}}\text{∣}}\text{.}$$

For module *q*, raw exposure was the unweighted mean of $\text{E}_{\text{gmp}}^{\left( \text{K} \right)}$ across its unique herb-association profiles. To compare the three modules within each PC, these raw exposures were normalized to sum to one:

$$\text{C}_{\text{qmp}}^{\left( \text{K} \right)}\text{=}\frac{\text{R}_{\text{qmp}}^{\left( \text{K} \right)}}{\sum_{\text{q}^{\text{'}}\text{=1}}^{\text{3}} \text{R}_{\text{q}^{\text{'}}\text{mp}}^{\left( \text{K} \right)}}\text{.}$$

Thus, a normalized proportion of one third represents equal relative exposure of the three modules. Each unique herb-association profile contributed equally to the module score.

The primary analysis used the top 200 herbs on each aligned PC. Module proportions were summarized across 10 independently trained and PC-aligned models as mean ± standard deviation. The literature-guided correspondence was fixed as thermogenic and mitochondrial oxidative metabolism for PC1, airway innate-inflammatory response for PC2, and fatty-acid oxidation and cholesterol handling for PC3. Within each PC, the corresponding module was compared with each of the other two modules using two-sided paired *t*-tests across repeat models, with Holm correction across the six fixed comparisons.

As a null control, herb identities were randomly reassigned to PC-score positions while retaining the herb–target network, GO-module definitions, aligned PC structure, and complete scoring procedure. The same herb-label permutation was applied across aligned models within each iteration, and normalized module proportions were recalculated for 1000 permutations. Sensitivity to the number of selected herbs was evaluated using *K* = 100, 150, 200, 250, and 300, without redefining the GO modules or refitting TCM-ES. The analysis therefore tested whether the literature-guided functional modules showed reproducible and non-random preferential distributions along the learned principal directions; it was not designed to establish a molecular mechanism for an individual herb, protein, or PC.

### PPI-network concordance

First, we evaluated herb–herb, disease–disease, disease–herb, and protein–protein relationships using the human PPI network described in Note S6. Each herb was represented by its mapped target-protein module and each disease by its associated-gene module. Herb–herb and disease–disease molecular relationships were quantified using PPI module separation [32], which compares between-module shortest-path distances with the corresponding within-module distances. Disease–herb separation was calculated analogously between disease-gene and herb-target modules. For two protein modules *A* and *B*, module separation was defined as

$$\text{s}_{\text{AB}}\text{=}\text{d}_{\text{AB}}\text{-}\frac{\text{d}_{\text{AA}}\text{+}\text{d}_{\text{BB}}}{\text{2}}\text{,}$$

where *d_AB_* is the mean shortest-path distance between proteins in modules *A* and *B*, and *d_AA_* and *d_BB_* are the corresponding mean within-module shortest-path distances. Lower separation values therefore indicate greater PPI-network proximity between the two modules. Module-separation values were standardized within each focal entity, with lower values indicating closer-than-average PPI relationships. For protein–protein analyses, molecular proximity was quantified directly by shortest-path length between the corresponding proteins in the PPI network.

Global embedding–PPI concordance: We then evaluated global relationships across ten independently trained TCM-ES repeat models. Within each model, the embedding distance of every herb–herb, disease–disease, disease–herb, or protein–protein pair was converted to a percentile rank, so that distances could be compared across models with different numerical scales. For each identical pair, we then took the median distance percentile across the ten models to construct a training-seed median-rank consensus. Embedding vectors themselves were not averaged across models. For the herb–herb, disease–disease, and disease–herb analyses, standardized PPI module separation was summarized across equal-frequency bins of the consensus embedding-distance percentile. Within each bin, the mean module separation and its standard error were calculated, and the distance-dependent relationship was summarized using a linear fit with a 95% confidence interval. Pearson correlation coefficients were calculated across the displayed bin means rather than across all raw entity pairs. For the protein–protein analysis, consensus embedding-distance percentiles were compared across groups defined by PPI shortest-path length.

Local-neighbour concordance. We next examined whether embedding–PPI concordance was also present locally among the nearest TCM-ES neighbours. For each focal herb or disease, the remaining candidate entities were ranked using embedding distance alone. At each prespecified neighbourhood size *k*, the PPI relationships of the top-*k* nearest embedding neighbours were compared with those of the remaining candidate entities. For herb–herb, disease–disease, and disease–herb analyses, the local effect was calculated as the difference between the distance-weighted mean standardized PPI separation among the top-$\text{k}$ neighbours and the mean separation among the remaining entities; negative values therefore indicate greater PPI-network proximity among local TCM-ES neighbours. Bootstrap resampling with 1000 resamples was used to obtain 95% confidence intervals for these local effects.

Training-seed stability. To assess whether the global embedding–PPI concordance depended disproportionately on any individual training run, we performed leave-one-seed-out analyses. Each of the ten independently trained models was excluded in turn, and the median embedding-distance percentile for every identical entity pair was recalculated across the remaining nine models. The same global analysis described above was then repeated for each of the ten leave-one-seed-out consensuses. For herb–herb, disease–disease, and disease–herb relationships, standardized PPI module separation was summarized across embedding-distance-percentile bins; for protein–protein relationships, embedding-distance percentiles were summarized across PPI shortest-path groups.

Entity-level stability. To assess sensitivity to shared-entity dependence, each herb, disease, or protein was sequentially removed together with all associated pairs, and the corresponding global concordance statistic was recalculated.

Permutation controls. We performed 1000 permutation analyses combining entity-identity shuffling with degree-preserving PPI permutation. In each iteration, herb, disease, and protein identities were independently shuffled among their corresponding embedding positions. PPI node labels were permuted only among nodes with identical degree. The PPI network itself, including its edges and shortest-path structure, therefore remained unchanged, while the exact PPI degree of each mapped protein or gene was preserved. The herb–target and disease–gene annotation structures were also retained unchanged. For the herb–herb, disease–disease, and disease–herb analyses, PPI module separation was recalculated using the permuted PPI mapping and compared with the identity-shuffled embedding relationships. For the protein–protein analysis, identity-shuffled embedding distances were compared with shortest-path groups obtained under the same degree-preserving PPI permutation. The global and local analyses were then repeated using the same procedures as for the observed data. For the global analyses, two-sided empirical permutation *p* values were calculated using the binned Pearson correlation for herb–herb, disease–disease, and disease–herb relationships and the pair-level Spearman correlation between ordinal PPI shortest-path group and embedding-distance percentile for protein–protein relationships. For the local-neighbour analyses, one-sided empirical permutation *p* values were used to test the prespecified direction of lower PPI separation among nearest embedding neighbours. These controls tested whether the observed embedding–PPI concordance required the correct correspondence among TCM-ES positions, biomedical entity identities, and PPI-network positions after preserving the underlying annotation structures, PPI topology, and node degree.

### Diagnostics and robustness of biomedical mapping

Semantic coverage of symptom terms used for downstream mapping

Human diseases and clinical symptom patterns that contained terms not present as exact standardized TCM symptom identities were represented through the same pretrained GUSE semantic encoding used for symptom inputs to the TCM-ES. We therefore examined the semantic coverage of the symptom terms used for downstream mapping relative to the 1436 standardized TCM symptoms used to construct the core TCM-ES. GUSE vectors were generated for the standardized TCM symptoms, the modern-medicine disease-associated symptom terms used to construct disease symptom patterns, and the symptom terms used in the COVID-19 clinical dataset. The resulting semantic vectors were jointly visualized using UMAP (Figure S23).

The three groups occupied broadly overlapping regions of the semantic space, with most disease-associated and COVID-19 symptom terms falling within the broader semantic domain represented by the standardized TCM symptom vocabulary (Figure S23). This overlap supports the use of the shared GUSE-based symptom encoding for downstream symptom-pattern mapping, while the subsequent disease and clinical embeddings were generated only after passing these semantic representations through the fixed symptom-pattern encoder.

Sensitivity of PC-associated GO-module relationships

The primary GO-based analysis evaluated the relative exposure of three literature-supported Biological Process modules among the top 200 herbs projected along each aligned principal component, as described in GO-based analysis of TCM-ES principal directions and Table S11. To examine whether the observed PC-associated module patterns depended on this herb-selection threshold, the analysis was repeated using the top 100, 150, 200, 250, and 300 herbs along each PC without redefining the GO modules, refitting TCM-ES, or changing the PC-alignment procedure (Figure S28).

Across the evaluated thresholds, the same relative module pattern was retained. The thermogenic and mitochondrial oxidative-metabolism module remained most prominent along PC1, the airway innate-inflammatory-response module remained most prominent along PC2, and the fatty-acid-oxidation and cholesterol-handling module remained most prominent along PC3 (Figure S28A-C). Although the relative module proportions varied with the number of selected herbs, the correspondence between each leading PC and its literature-supported biological-process module was therefore not restricted to the primary top-200-herb threshold.

PPI-module connectivity

The embedding–PPI analyses represented each disease by its associated-gene set and each herb by its target-protein set and quantified relationships between these sets using PPI module separation. We first examined whether the corresponding gene and target sets exhibited network connectivity beyond that expected from set size alone. For each disease-associated gene set, the size of its largest connected component in the human PPI network was compared with the corresponding distribution obtained from 1000 random gene sets containing the same number of genes. The same procedure was applied to herb-target sets using size-matched random protein sets. Giant-component size was summarized as a *z*-score relative to the corresponding random distribution (Figure S29).

The observed *z*-score distributions were shifted toward positive values for both disease-associated gene sets and herb-target sets (Figure S29A, B), indicating that these sets tended to form larger connected components in the PPI network than size-matched random sets. This network connectivity supports their treatment as gene or target modules in the subsequent module-separation analyses.

Local embedding–PPI concordance

The global analyses in the main text showed that increasing embedding distance was accompanied by increasing PPI separation for herb–herb, disease–disease, and disease–herb relationships, and that protein pairs connected by shorter PPI paths were also positioned closer in TCM-ES. We further examined whether this concordance was present locally among the nearest embedding neighbours rather than arising only from the global distance trend.

For each focal herb or disease, candidate entities were ranked by embedding distance alone, and the nearest *k* = 5, 10, 20, or 50 neighbours were compared with the remaining entities. For herb–herb, disease–disease, and disease–herb relationships, the local effect was calculated as the difference between the distance-weighted mean standardized PPI separation of the top-*k* embedding neighbours and the mean separation of the remaining candidates. Negative values therefore indicate that the nearest neighbours in TCM-ES also have more proximal target or gene modules in the PPI network (Figure S30).

Across all three relationship types, the local effects were consistently negative across the evaluated neighbourhood sizes, indicating greater PPI-network proximity among nearby TCM-ES neighbours than among the remaining entities (Figure S30A-C). In the corresponding permutation controls, herb or disease identities were shuffled together with degree-preserving PPI permutation. The local effects were instead centred near zero (Figure S30A-C, grey lines). Thus, the local embedding–PPI concordance depended on the correspondence between entity identity, embedding position, and the PPI positions of the associated genes or targets.

Robustness of global embedding–PPI concordance

We examined whether the global embedding–PPI relationships were disproportionately influenced by any individual TCM-ES training run. Starting from the ten independently trained models, each model was omitted in turn, and the median embedding-distance percentile for each identical entity pair was recalculated across the remaining nine models. The global herb–herb, disease–disease, disease–herb, and protein–protein analyses were then repeated for each of the ten leave-one-seed-out consensuses (Figure S31).

The herb–herb, disease–disease, and disease–herb analyses consistently retained increasing PPI separation with increasing embedding-distance percentile across the leave-one-seed-out consensuses. Similarly, protein pairs connected by progressively longer PPI shortest paths retained progressively larger embedding-distance percentiles (Figure S31). The global embedding–PPI concordance was therefore not driven by any single independently trained model. Node-level leave-one-out analyses likewise retained the observed association direction for every entity exclusion. The corresponding ranges were *r*_bin_ = 0.862-0.922 (herb–herb), 0.837-0.901 (disease–disease), 0.809-0.854 for disease leave-one-out and 0.713-0.873 for herb leave-one-out in the disease–herb analysis, and *ρ* = 0.0666-0.0702 (protein–protein).

## Note S8 Disease organization, knowledge graph, rheumatoid arthritis case study, and interactive exploration

### Disease organization and knowledge graph

Disease-neighbourhood network: To examine the organization of symptom-defined diseases in the integrated TCM-ES, we constructed a *k*-nearest-neighbour (KNN) network based solely on pairwise Euclidean distances between disease embeddings. The analysis included the 79 diseases for which both symptom-derived embeddings and associated-gene annotations were available (see Note S6). For each disease, the *k* nearest diseases were identified, and an undirected edge was retained only when the two diseases ranked within each other’s top-*k* neighbours. Edge width was determined by embedding proximity. For visualization, disease nodes were annotated using their top-level MeSH Disease categories, and edges were additionally labelled according to whether the connected disease pair shared annotated genes and/or associated symptoms. These annotations were not used to construct the KNN network. To compare the embedding-based disease organization with conventional biomedical classification, each disease was represented by its set of top-level MeSH Disease [C] categories. For two diseases *i* and *j*, MeSH-category similarity was quantified using the Jaccard index

$$\text{J}\left( \text{i} , \text{j} \right)\text{=}\frac{\text{∣}\text{C}_{\text{i}}\text{∩}\text{C}_{\text{j}}\text{∣}}{\text{∣}\text{C}_{\text{i}}\text{∪}\text{C}_{\text{j}}\text{∣}}\text{,}$$

where *C_i_* and *C_j_* denote their respective sets of MeSH categories. Disease pairs with *J* > 0.6 were classified as having similar MeSH classifications, whereas pairs with no overlapping MeSH categories (*J* = 0) were classified as having distinct classifications. Embedding distances between these groups were compared to assess concordance between TCM-ES disease proximity and MeSH classification (Figure S32). Robustness of the disease-network topology to the choice of *k* was examined by reconstructing the network using alternative neighbourhood sizes while retaining the same disease embeddings and mutual-neighbour criterion (Figure S33).

Knowledge graph construction: Human diseases and the molecular and therapeutic entity types described above (target proteins, herbal compounds, herbs, formulas, and supported modern drugs) were represented in the fixed integrated TCM-ES using the procedures described in Note S2 and Note S6. The Euclidean distances from each disease to targets, compounds, drugs and herbal therapies were calculated. Distances were calculated separately between each disease and each candidate entity type. Because the distance distributions differed among entity types, distances were standardized within each entity type before cross-type visualization and ranking. For each disease, candidate entities within each data type were ranked according to their embedding proximity, and the top-ranked neighbours were connected to the disease to construct an exploratory TCM-informed knowledge graph. These edges represent proximity-based associations in the integrated TCM-ES and were used for hypothesis-generating prioritization. They were not interpreted as evidence of causal relationships, therapeutic efficacy, or validated drug–target–disease mechanisms.

For herbal-therapy candidates, the analysis was restricted to plant-derived herbs, excluding herbs of animal or mineral origin, and to 253 classical formulas included in the authoritative textbook Prescription of Chinese Medicine [50]. When an excluded herb occurred among the top-ranked neighbours, it was skipped and the next eligible plant-derived herb was retained.

Modern-drug neighbours were restricted to the supported mappings defined in Note S6. Detailed analyses of modern drug–disease relationships are reported in Note S9.

### Disease network robustness

Concordance between disease proximity and MeSH classification

The disease-neighbourhood network in the integrated TCM-ES was constructed from the symptom-derived embeddings of 79 diseases with available disease-associated gene annotations, as described in Disease organization and knowledge graph. Although MeSH annotations were not used to construct the disease embeddings or define network edges, the network showed visible grouping of diseases with related MeSH classifications in the main analysis. We therefore quantitatively examined the relationship between disease proximity in TCM-ES and conventional MeSH disease classification (Figure S32).

For each disease, the set of its top-level MeSH Disease categories was used as an external classification annotation. For a pair of diseases *i* and *j*, similarity of their MeSH classifications was quantified using the Jaccard index between their respective category sets, as defined in Disease organization and knowledge graph. Disease pairs with MeSH-category Jaccard similarity greater than 0.6 were classified as having similar MeSH classifications, whereas pairs with no overlapping MeSH categories were classified as having distinct classifications. The Euclidean distances between the corresponding disease embeddings in TCM-ES were then compared between these two groups. Disease pairs with similar MeSH classifications showed shorter embedding distances than pairs with distinct MeSH classifications (Figure S32). Thus, disease proximity derived from symptom-pattern embeddings showed concordance with an independently defined biomedical disease-classification system.

Robustness of the disease-neighbourhood network

The primary disease-neighbourhood network was constructed by connecting mutually nearest diseases according to their embedding distances in TCM-ES. For each disease, its seven nearest neighbours were identified, and an undirected edge was retained only when both diseases ranked within each other's seven nearest neighbours. Network construction used only disease embedding proximity. The primary *k* = 7 setting was chosen to balance network connectivity and edge density. With 79 diseases in the network, *k* = 7 corresponds to fewer than 10% of the possible neighbours for each disease before applying the mutual-neighbour criterion, thereby retaining a relatively local network structure. MeSH categories and shared gene or symptom annotations were introduced subsequently for visualization and were not used to determine the network edges.

To examine sensitivity to the neighbourhood-size parameter, the same disease embeddings and mutual-neighbour criterion were retained while the network was reconstructed using *k* = 6 and *k* = 8 (Figure S33A, B). The alternative networks showed similar overall clustering organization to that obtained with the primary *k* = 7 setting, supporting stability around the primary *k* = 7.

### TCM-informed characterization of rheumatoid arthritis

RA-centred molecular and therapeutic candidate prioritization

Rheumatoid arthritis (RA) was used as an illustrative example to examine the molecular and therapeutic entities prioritized around a symptom-defined disease in the integrated TCM-ES. Using the general RA embedding derived from its literature-associated symptom pattern, target proteins, herbal compounds, supported modern drugs, herbs, and classical formulas were ranked separately according to their embedding proximity to RA, following the knowledge-graph procedure described in Disease organization and knowledge graph. Because the background distance distributions differed among entity types, rankings were performed within each data type rather than by directly comparing absolute distances across heterogeneous entity classes. The resulting RA-centred neighbourhood is illustrated in Figure 2G, and the proximity-ranked candidates together with literature evidence for previously reported RA-related relationships are summarized in Table S12.

Several of the highly ranked molecular and therapeutic entities had previously reported relationships with RA (Table S12). Among the target-protein candidates, BTRC and MC1R were positioned among the nearest RA-associated targets. BTRC has been implicated in inflammatory regulation in RA fibroblast-like synoviocytes through the miR-10a–BTRC–NF-κB regulatory axis [51], whereas MC1R has been reported in the context of inflammatory and fibrotic disorders including RA [52]. Other proximity-ranked targets with literature-supported RA-related associations are also listed in Table S12.

Among the compound and supported modern-drug candidates, Selenomethionine was one of the closest entities to RA in both corresponding candidate sets, and previous experimental work has reported effects of selenium supplementation on arthritis development and severity [53]. Arbutin was also ranked among the nearby supported modern drugs and has previously been examined in an experimental arthritis model [54]. These literature associations provide examples of previously reported RA-related relationships among the proximity-ranked candidates; they do not constitute prospective validation of the ranking procedure.

The RA-centred ranking also identified conventional herbal therapies with previously reported relationships to arthritis. Du-Zhong (Eucommiae Cortex) was the nearest eligible plant-derived herb in the RA ranking (Table S12) and has been examined in previous studies of arthritis-related pharmacological activity [55]. Among the classical formulas, Du-Huo-Ji-Sheng-Tang was among the nearest proximity-ranked formulas, with previous experimental evidence available for RA- or arthritis-related effects (Table S12) [56]. These examples illustrate how the integrated embedding can place molecular entities and traditional therapeutic entities within the same disease-centred exploratory framework. Additional supporting literature for other proximity-ranked candidates is provided in Table S12 [57–77].

Characterization of Cold and Heat-pattern RA groups using detailed symptom profiles

The general RA embedding used for the disease-level knowledge graph represents RA through a single literature-derived symptom pattern and therefore does not explicitly encode clinical heterogeneity within the disease. We consequently examined how the reported Cold- and Heat-pattern group symptom profiles were represented in TCM-ES (Figure S36).

The group information was obtained from a previous study of 21 female patients with RA who had been classified as having Cold or Heat patterns according to their TCM symptom profiles. That study additionally measured gene-expression profiles in CD4+ T cells and identified genes associated with the two subtype classifications. We used the reported subtype-specific symptom profiles to construct separate Cold- and Heat-type RA symptom-pattern embeddings and used the reported subtype-associated genes as external annotations for post hoc comparison.

Cold-type RA was represented by 10 reported symptoms and Heat-type RA by 13 symptoms, with three symptoms shared between the two subtype profiles. Each complete subtype-specific symptom set was passed through the fixed symptom-pattern encoder to generate a subtype embedding, while the individual constituent symptoms were mapped separately for visualization. The resulting embeddings were projected onto the PC1-PC2 plane together with their associated symptoms and molecular entities (Figure S36). The symptom distributions for the two subtypes occupied partially distinct regions, whereas the three shared symptoms were positioned between or within the overlapping portions of the two distributions.

Among the subtype-associated genes reported in the source study, six could be represented in the integrated TCM-ES: MSH6 for Cold-type RA and CTBP2, GLUL, PIGK, DIABLO, and GABBR1 for Heat-type RA. MSH6 was positioned closer to the Cold-type RA embedding, whereas the five Heat-associated genes were collectively positioned toward the Heat-type region (Figure S36). These relationships provide a molecular annotation of the subtype-specific embedding organization but were not used to construct the subtype embeddings.

The Cold-type RA embedding was positioned more toward the PC1-associated region, whereas the Heat-type embedding was positioned more toward the PC2-associated region (Figure S36). Given that the leading PCs show broader post hoc associations with different biological-process modules (see Note S7), this separation provides an additional descriptive view of subtype heterogeneity rather than a direct mechanistic assignment of Cold and Heat patterns to individual biological pathways.

RA-indicated drugs were included in the same projection for exploratory comparison. Their positions relative to the general, Cold-type, and Heat-type RA embeddings differed across drugs (Figure S36), illustrating that more detailed symptom definitions can alter the relative neighbourhood of a disease representation.

### Web-based toolbox

To facilitate the practical use of the TCM-ES, we developed a web-based toolbox, TCM Embedding Space, for interactive exploration, visualization, and quantitative analysis of TCM-related and biomedical entities. The toolbox is publicly available at https://www.tcmes.net/. Built upon the trained Transformer-based framework described in the main text, the toolbox allows users to browse pre-computed embeddings, generate embeddings for customized inputs, retrieve spatially related entities, inspect model attention, and conduct exploratory knowledge-graph-based candidate prioritization. By integrating symptoms, herbs, formulas, herbal compounds, drugs, targets, and diseases within a unified embedding space, the toolbox provides an accessible online resource for quantitative TCM research and integrative biomedicine.

The toolbox consists of the following major functional modules.

(1) Data Browser. The Data Browser module enables interactive visualization of pre-computed entity embeddings in the TCM-ES. Users can browse seven types of entities: symptoms, formulas, herbs, compounds, drugs, targets, and diseases. The interface provides a three-dimensional PCA visualization of the embedding space, supporting rotation, zooming, panning, and point selection. By selecting a data point, users can view the corresponding entity information and inspect its spatial location within the TCM-ES. The module also supports fuzzy search, allowing users to retrieve entities using partial keywords. Multiple entities can be selected across sequential searches for comparative inspection. For downstream analysis, users can download the coordinates of selected entities in two formats: the original 256-dimensional embedding vectors and the six-dimensional PCA-reduced coordinates, of which the first three dimensions are used for visualization.

(2) TCM Representation. The TCM Representation module converts user-defined symptom patterns or herb combinations (e.g., a formula) into quantitative coordinates within the TCM-ES. For symptom inputs, the module uses semantic representations generated by the Google Universal Sentence Encoder (GUSE), allowing user-provided symptom phrases to be processed in the same input format used by the trained symptom encoder. For herb-combination inputs, the module generates formula-level representations based on the trained herb encoder. The resulting embeddings can be downloaded as 256-dimensional original vectors or six-dimensional PCA coordinates. These outputs can also be directly transferred to the Relation Exploration module, allowing users to identify nearby formulas, herbs, compounds, drugs, targets, or diseases in the TCM-ES. Batch processing through file upload is supported for high-throughput analyses.

(3) Generation. The Generation module performs bidirectional inference between symptom patterns and herb combinations based on the learned symptom-pattern–formula mapping. In the symptom-to-herb mode, users can input a symptom pattern and obtain model-generated candidate herb combinations. In the herb-to-symptom mode, users can input an herb combination and obtain model-generated associated symptom patterns. This module provides an interactive implementation of the model’s encoding–decoding capacity, allowing users to explore learned symptom-pattern–formula correspondences.

(4) Relation Exploration. The Relation Exploration module identifies entities that are spatially proximal to a query point in the TCM-ES. Users can either select a query entity from the built-in database or upload customized embedding coordinates generated from the TCM Representation module. The module retrieves nearest neighbours from six entity types: formulas, herbs, compounds, drugs, targets, and diseases. This function supports exploratory downstream analyses, including retrieving formulas near a symptom pattern, examining neighbouring herbs or herbal compounds, exploring supported modern-drug and target-protein neighbours of diseases, and examining disease–disease relationships in the embedding space. These proximity-based results are intended for exploration and hypothesis generation and do not constitute validated therapeutic, drug-repurposing, or target predictions.

(5) Analysis Workbench. The Analysis Workbench provides a set of analytical tools corresponding to major analyses described in the main text. TCM Formula Analysis. This tool computes attention matrices for input symptom pattern and herb combinations, allowing users to inspect how the model assigns attention during symptom-pattern–formula inference. Attention values reflect context-dependent model weights and should not be interpreted as direct measures of therapeutic importance. Both raw and normalized attention matrices can be downloaded for further analysis.

3D PCA Visualization. This tool allows users to visualize customized coordinates in the PCA-reduced TCM-ES. Users can upload coordinates from different entity types, customize labels and display settings, and calculate pairwise Euclidean distances among selected entities.

(6) Data Export and Workflow Integration. All modules support data export for downstream analysis. Embedding vectors, PCA coordinates, prediction results, distance matrices, attention matrices, and network files can be downloaded in standard formats such as CSV or TXT. The toolbox is designed to support workflow integration across modules. Together, this web-based toolbox provides an interactive implementation of the TCM-ES framework for data exploration and downstream analysis. It provides an accessible resource for exploring quantitative TCM embeddings, analysing symptom–herb relationships, and performing exploratory, TCM-informed candidate prioritization.

## Note S9 Modern-drug mapping diagnostics and exploratory drug–disease relationships

### Extension and harmonization of additional drug–disease and reporting data

The primary modern-drug analysis used the literature-supported drug–disease indication dataset, together with the modern-drug mapping criteria defined in Note S6. Additional drug, indication, structural, and adverse-event data were subsequently assembled to examine the applicability of the modern-drug mapping procedure, the assumptions underlying the FAERS-derived observational measure, and the robustness of the observed drug–disease relationships.

To extend the drug–disease analysis beyond the primary literature-supported dataset [37] and to obtain a more comprehensive and recent source of adverse-event information, we assembled additional modern-drug and drug–disease data following the general framework used in the previous study. Approved drugs were obtained from DrugBank [39], established drug–disease indication relationships were obtained from MEDI-2 [40], and adverse-event reports were retrieved from the FDA Adverse Event Reporting System (FAERS) through the openFDA API [38]. MEDI-2 contains more than 30,000 established drug–disease indication relationships curated from clinical and regulatory information. The openFDA records used in this analysis spanned 2004 through the second quarter of 2024 and comprised more than 70 million report entries. Drug structural information was subsequently obtained from PubChem, and estimated U.S. prescription volumes used in the reporting-volume diagnostic described below were obtained from the ClinCalc DrugStats database (Note S6).

For each openFDA report, drug names, corresponding therapeutic indications, and reported adverse-event reactions were extracted. A single report could contain multiple drugs and their corresponding indications. Because openFDA contains heterogeneous terminology, including brand and generic drug names, non-standard disease descriptions, colloquial expressions, and typographical variants, drug and disease terms were standardized before constructing the additional drug–disease reporting dataset. To reduce noise from infrequently occurring terms, only drug and disease terms appearing at least 200 times in the retrieved openFDA reports were considered for subsequent terminology alignment.

Disease terms from MEDI-2 and openFDA were aligned to the standardized MeSH disease terminology used for the biomedical analyses. GUSE vectors were first generated for disease terms from MeSH, MEDI-2, and openFDA. Candidate mappings were identified when the cosine similarity between a MEDI-2 or openFDA disease term and a MeSH disease term exceeded 0.75. These preliminary semantic mappings were subsequently examined using GLM-4-Flash to remove inappropriate matches and generate standardized mappings of MEDI-2 and openFDA indication terms to MeSH diseases.

Drug terminology was aligned directly between MEDI-2 and openFDA. Brand and generic drug terms from the two resources were compared using structured language-model-assisted matching with GLM-4-Flash. To reduce dependence on a single language model, both the disease-term and drug-term alignment procedures were independently repeated using Qwen2.5-7B-Instruct, and only mappings retained by both procedures were used in the subsequent analyses. SMILES representations for the resulting matched drugs were retrieved from PubChem for structural encoding and modern-drug mapping.

### Mapping-confidence assessment of modern-drug embeddings

Modern drugs were mapped into the fixed TCM-ES using Encoder 3, which was trained on Mol2Vec structural representations of herbal compounds and their known associations with source herbs (see Note S6). Because Autoencoder 3 was trained exclusively within the chemical domain represented by herbal compounds, reliable representation of structurally distinct modern pharmaceuticals could not be assumed. We therefore assessed the applicability of the mapping procedure across the broader approved-drug collection before interpreting downstream drug–disease relationships.

For each modern drug, its 300-dimensional Mol2Vec structural vector was passed through Encoder 3 to obtain a 256-dimensional embedding, and Decoder 3 reconstructed the corresponding structural vector. Mapping reliability was assessed using two complementary criteria described in Note S6. First, structural reconstruction was evaluated by comparing the reconstructed Mol2Vec vector with the original structural vector of the drug and candidate reference structural vectors using cosine similarity. Second, the geometric position of the drug embedding was evaluated relative to the domain occupied by the core TCM-ES. The centroid of the core space was calculated from the symptom-pattern and formula embeddings of the historical formula records, and a drug was considered within the supported geometric domain when its embedding lay within twice the 95th-percentile distance of these core embeddings from the centroid.

Across 2610 approved-drug entries, 718 (27.5%) satisfied the high-confidence criterion, defined as top-1 structural reconstruction within the supported geometric domain. A further 460 entries (17.6%) were classified as intermediate confidence, with reconstruction ranks from second to fifth while remaining within the supported domain. The remaining 1432 entries (54.9%) were classified as low confidence because they lay outside the supported domain and/or their reconstructed structural vectors were not ranked among the top five candidates (Table S10A). Thus, 1178 of the 2610 approved-drug entries (45.1%) were supported under the relaxed top-5 reconstruction criterion.

Mapping confidence differed among representative chemical categories. Low-confidence mappings were observed for 30 of 33 peptide or peptide-like drugs (90.9%), 108 of 115 structurally complex antibiotics (93.9%), and 35 of 46 contrast or radiopharmaceutical agents (76.1%). In comparison, 14 of 54 vitamins, amino acids, or endogenous metabolites (25.9%) were classified as low confidence (Table S10B). These class-level patterns indicate that the herbal-compound-trained encoder has a restricted chemical applicability domain, particularly for structural classes poorly represented by the herbal-compound training data.

Within the original literature-supported drug–disease indication dataset, 94 of 238 drugs (39.5%) satisfied the primary structural-reconstruction and geometric-domain criteria and were retained for downstream analysis. Together with the requirement for an available symptom-derived disease embedding, this screening yielded the primary set of 125 drug–disease indication pairs involving 94 drugs and 27 diseases described in Note S6.

### Diagnostic assessment of the FAERS-derived observational measure

The primary drug–disease dataset included FAERS-derived Relative Efficacy (RE) scores reported in the previous study for a subset of the indication pairs [37]. Because FAERS is a spontaneous adverse-event reporting system rather than a clinical efficacy database, we treated RE as an indirect observational measure. Before examining its relationship with drug–disease proximity in TCM-ES, we evaluated the assumptions underlying its construction using the additional reporting data described above.

For motivation, if the total number of patients with a disease who received a given drug were $\text{N}_{\text{total}}$, and the number for whom treatment was ineffective were *N*_ineffective_, a treatment-success fraction could be expressed as

$$\text{E}\text{=1-}\frac{\text{N}_{\text{ineffective}}}{\text{N}_{\text{total}}}\text{.}$$

Neither quantity can be directly obtained from FAERS. The reporting-based approximation therefore assumes that the number of ineffective-treatment cases is proportional to the corresponding number of ineffectiveness reports in FAERS:

$$\text{N}_{\text{ineffective}}\text{=}\text{a}\text{ }\text{n}_{\text{ineffective}}\text{,}$$

where *n*_ineffective_ denotes the number of reports containing an ineffectiveness-related event for the corresponding drug–disease pair and *a* is an unknown proportionality constant assumed to be common across the comparisons.

A second assumption is required to approximate treatment or reporting volume. The number of reports corresponding to the most frequently reported adverse event for the drug, denoted *n*_top_, was used as a proxy for overall reporting volume:

$$\text{N}_{\text{total}}\text{=}\text{b}\text{ }\text{n}_{\text{top}}\text{,}$$

where *b* is another proportionality constant. Under these assumptions,

$$\text{E}\text{=1-}\frac{\text{a}\text{ }\text{n}_{\text{ineffective}}}{\text{b}\text{ }\text{n}_{\text{top}}}\text{.}$$

Because *a*/*b* is assumed to be constant across the evaluated comparisons, the ratio *n*_ineffective_ / *n*_top_ preserves the corresponding comparative ordering. The FAERS-derived observational score used in the analyses was therefore defined as

$$\text{RE=1-}\frac{\text{n}_{\text{ineffective}}}{\text{n}_{\text{top}}}\text{.}$$

Larger RE values consequently indicate fewer reported ineffectiveness events relative to the reporting-volume proxy.

For the independently assembled MEDI-2/openFDA data, adverse-event counts for each established drug–disease indication relationship were obtained by querying all retained combinations of the corresponding standardized drug and disease terms. The number of reports containing ineffectiveness-related events and the number associated with the most frequently reported adverse event were then used to reconstruct the reporting components of the RE score.

We specifically examined the assumption that $\text{n}_{\text{top}}\text{ }$provides a useful approximation of drug-reporting volume. Estimated U.S. prescription volumes for commonly used drugs in 2022 were obtained from ClinCalc. To reduce ambiguity arising from drugs used across many unrelated diseases, this analysis was restricted to drugs with relatively narrow indication profiles. For each drug, its established indications in MEDI-2 were assigned to MeSH disease categories. A drug was classified as having a narrow indication profile when at least 60% of its indicated diseases belonged to the same MeSH category. 96 drugs satisfying this criterion were also represented in the corresponding ClinCalc and openFDA data.

For each of these 96 drugs, adverse-event reports associated with its drug–disease usage records were collected, and the number of reports for each adverse-event type was counted. The number of reports corresponding to the most frequently reported adverse event was then compared with the estimated 2022 U.S. prescription volume of the same drug. A positive Pearson correlation was observed between the most-frequently-reported-event count and estimated prescription volume (Figure S34E). This relationship supports the use of *n*_top_ as an approximate reporting-volume proxy in constructing the FAERS-derived RE score. This diagnostic addresses the plausibility of this specific denominator assumption and does not independently establish RE as a validated quantitative measure of clinical therapeutic efficacy.

### Primary exploratory drug–disease relationships

We next examined the primary set of 125 literature-supported indication pairs involving 94 supported modern-drug mappings and 27 symptom-defined diseases, of which 77 pairs had available FAERS-derived RE scores. The mapped drugs and diseases occupied heterogeneous positions within the integrated TCM-ES, and established indication pairs spanned relatively close, intermediate, and distant embedding relationships (Figure S34A). Representative examples included Propranolol–Cardiac Arrhythmias among the relatively close indication pairs, Tolbutamide–Diabetes Mellitus at an intermediate distance, and Ibuprofen–Rheumatoid Arthritis among the relatively distant pairs.

The background distance distributions differed substantially among individual drugs and diseases. For example, some diseases were positioned relatively close to many of the mapped drugs, whereas others were more distant from the mapped-drug set overall. Consequently, absolute Euclidean distance alone could reflect both pair-specific proximity and the broader spatial position of the corresponding drug or disease. Drug–disease relationships were therefore additionally evaluated relative to their reference distance distributions.

Established indication pairs showed a predominantly negative distribution of embedding-distance *z*-scores (Figure S34B). Negative values indicate that the corresponding drug and disease were positioned closer than expected relative to the reference drug–disease relationships. Thus, known drug–disease indication pairs tended to occupy closer-than-reference positions in the integrated TCM-ES.

To account for the heterogeneous background distance distributions of both drugs and diseases, mutual drug–disease matching was further quantified using the bidirectional *z*-score (BDZ) defined in Note S6. For each drug–disease pair, BDZ combines the standardized position of the disease within the distance distribution from the corresponding drug with the standardized position of the drug within the distance distribution from the corresponding disease. Lower BDZ values therefore indicate closer mutual matching relative to both background distributions and were interpreted as an embedding-based relationship metric rather than as a measure of therapeutic efficacy.

Among the 77 primary indication pairs with available RE scores, lower BDZ values were associated with higher values of the FAERS-derived observational measure (Figure S34C). Thus, drug–disease pairs showing closer mutual matching in TCM-ES tended to have fewer reported ineffectiveness events relative to the reporting-volume proxy used in the RE score.

We further examined this relationship at the disease level for diseases represented by at least three indicated drugs with available RE scores. Both the BDZ distributions of the indicated drugs and their corresponding FAERS-derived RE distributions varied among diseases (Figure S34D). These disease-level patterns illustrate the heterogeneity of drug–disease relationships in the integrated embedding space and further motivate the use of relative, entity-specific matching measures rather than direct comparisons of absolute embedding distance across diseases. The association with RE remains an exploratory comparison with spontaneous-reporting data and does not establish differences in clinical efficacy among diseases or drugs.

### Sensitivity analyses across alternative datasets and analytical settings

We further examined whether the observed drug–disease proximity patterns and BDZ–RE association depended on the specific modern-drug screening criterion, disease-symptom representation, or adverse-event dataset used in the primary analysis. The analyses were repeated under four alternative settings (Figure S35).

First, the primary geometric-domain criterion was retained while the structural-reconstruction criterion was relaxed. Rather than requiring the reconstructed Mol2Vec representation of a drug to rank first in similarity to its original representation, the drug was retained when its original representation ranked among the five most similar candidates. This relaxed criterion yielded 186 established drug–disease indication pairs involving 134 drugs and 29 diseases, of which 113 pairs had available RE scores (Figure S35A, E).

Second, an alternative stricter reconstruction assessment was applied while retaining the same geometric-domain criterion. For each evaluated drug, the reconstructed Mol2Vec vector was required to be more similar to its own original structural representation than to the original representations of any other drugs in the evaluated indication set. This setting yielded 135 indication pairs involving 102 drugs and 27 diseases, including 76 pairs with available RE scores (Figure S35B, F).

Third, we examined sensitivity to the representation of disease-associated symptoms. The primary modern-drug screening criteria were retained, but disease embeddings were recalculated using the refined symptom sets incorporating Mayo Clinic information described in Note S6. The refined symptoms supplemented the literature-derived disease-associated symptom information after semantic redundancy and compatibility filtering. This analysis retained 125 indication pairs involving 94 drugs and 27 diseases (Figure S35C, G).

Fourth, we examined sensitivity to the adverse-event reporting dataset. The primary supported drug–disease set and the corresponding embeddings were retained, whereas the FAERS-derived observational scores were recalculated using the independently processed openFDA records described in Note S6, covering reports from 2004 through the second quarter of 2024. The standardized MEDI-2/openFDA drug and disease mappings described above were used to retrieve the corresponding reporting records (Figure S35D, H).

For each alternative setting, the proximity of established indication pairs was first evaluated using the same embedding-distance *z*-score analysis as in the primary dataset. Across the four settings, the *z*-score distributions remained predominantly shifted toward negative values, indicating that established indication pairs generally retained closer-than-reference embedding relationships (Figure S35A-D). For pairs with available RE scores, the relationship between BDZ and the FAERS-derived observational measure was then re-evaluated. Lower BDZ values remained associated with higher RE values across the corresponding alternative analyses (Figure S35E-H).

Together, these sensitivity analyses indicate that the observed exploratory drug–disease relationships were retained under alternative modern-drug mapping criteria, a refined disease-symptom representation, and an independently processed, more recent FAERS/openFDA dataset. The RE-based analyses nevertheless remain associations with an indirect spontaneous-reporting measure and should not be interpreted as independent clinical validation of drug efficacy.

### Statistical analysis

Statistical tests, uncertainty estimates, and resampling procedures were specified according to the structure of each analysis and are described in the corresponding Methods, Supporting Results, and figure legends. Unless otherwise specified, inferential tests were two-sided. Statistical reporting emphasized the magnitude and uncertainty of observed effects, including effect sizes or model coefficients, 95% confidence intervals where applicable, analysed sample sizes, and *p* values, rather than relying on statistical significance alone.

For correlation and regression analyses, the corresponding Pearson or Spearman correlation coefficient or regression coefficient was reported together with its uncertainty and sample size where applicable. For the adjusted clinical analysis, regression coefficients were reported with 95% confidence intervals and *p* values, with patient-condition–formula distance standardized within prescribed-formula groups as described in Note S5.

For analyses involving multiple inferential comparisons, multiplicity correction was applied as defined for the corresponding analysis. In particular, the GO-based PC analysis used Holm correction across the fixed module comparisons (see Note S7), whereas the attention-guided perturbation analysis used Benjamini–Hochberg correction. Adjusted *p* values were reported where applicable.

Bootstrap and permutation procedures were used to quantify uncertainty or construct empirical null distributions in analyses where the observations were not adequately summarized by conventional parametric assumptions. The number of resamples, resampling unit, and construction of the corresponding null distribution are specified with each analysis. Trend analyses used linear fits with 95% confidence intervals rather than LOWESS smoothing where inferential trends were displayed.

For visualization of the Cold- and Heat-associated symptom distributions in Figure S36, covariance ellipses were drawn from the two-dimensional PC1-PC2 coordinates of the corresponding symptoms. These ellipses were used descriptively and were not interpreted as inferential confidence regions.

# References

1. Liu, Juan, Wuwen Feng, Cheng Peng. 2021. “A song of ice and fire: cold and hot properties of Traditional Chinese Medicines.” *Frontiers in Pharmacology* 11: 598744. <https://doi.org/10.3389/fphar.2020.598744>

2. Ru, Jinlong, Peng Li, Jinan Wang, Wei Zhou, Bohui Li, Chao Huang, Pidong Li, et al. 2014. “TCMSP: a database of systems pharmacology for drug discovery from herbal medicines.” *Journal of Cheminformatics* 6: 13. <https://doi.org/10.1186/1758-2946-6-13>

3. Xue, Ruichao, Zhao Fang, Meixia Zhang, Zhenghui Yi, Chengping Wen, Tieliu Shi. 2013. “TCMID: traditional Chinese medicine integrative database for herb molecular mechanism analysis.” *Nucleic Acids Research* 41: D1089–D1095. <https://doi.org/10.1093/nar/gks1100>

4. Institute of Information on Traditional Chinese Medicine, China Academy of Chinese Medical Sciences. (September 1, 2021, date last accessed). National Database for Traditional Chinese Medicine. <https://cintmed.cintcm.cn/cintmed/main.html>.

5. Wu, Min, Tieju Ma, Yan Zhu, Hongtao Ren, Lu Fu. 2024. “The evolution of Traditional Chinese Medicine as recombinant inventions.” *Proceedings of the National Academy of Sciences* 121: e2400812121. <https://doi.org/10.1073/pnas.2400812121>

6. Xia, Fei, Qingchao Tu, Yinan Liu, Junzhe Zhang, Mengyue Fan, Linying Zhong, Yuanfeng Fu, et al. 2026. “Multifunctional copper ion-mediated carrier-free scutellarin hydrogel for diabetic wound healing.” *Advanced Healthcare Materials* 15: e03562. <https://doi.org/10.1002/adhm.202503562>

7. Fu, Yuanfeng, Fei Xia, Lingling Sun, Minyi Guan, Qingchao Tu, Xiangjun Qi, Jigang Wang, Lizhu Lin, Chong Qiu. 2025. “Anti-lung cancer therapy using nano-assembly particles of traditional Chinese medicine formula.” *Materials Today Bio* 35: 102502. <https://doi.org/10.1016/j.mtbio.2025.102502>

8. Zhang, Junzhe, Huanhuan Pang, Huan Tang, Qingchao Tu, Fei Xia, Hao Zhang, Yuqing Meng, Guang Han, Jigang Wang, Chong Qiu. 2025. “The pharmacodynamic and pharmacological mechanisms underlying nanovesicles of natural products: developments and challenges.” *Pharmacology & Therapeutics* 265: 108754. <https://doi.org/10.1016/j.pharmthera.2024.108754>

9. State Administration for Market Regulation, Standardization Administration of China. 2023. *Clinic terminology of traditional Chinese medical diagnosis and treatment—Part 1: Diseases (GB/T 16751.1-2023)*. Beijing: Standards Press of China.

10. Li, Jingbo, Li Ma. 2015. *Terminology specification of common clinical symptoms in TCM (Revised)*. Beijing: China Medical Science Press.

11. World Health Organization. (June 10, 2022, date last accessed). International Classification of Diseases, 11th Revision (ICD-11). <https://icd.who.int/>.

12. State Administration for Market Regulation, Standardization Administration of China. 2021. *Clinic terminology of traditional Chinese medical diagnosis and treatment—Part 2: Syndromes/patterns (GB/T 16751.2-2021)*. Beijing: Standards Press of China.

13. Sun, Junyi. 2012. Jieba: Chinese text segmentation. <https://github.com/fxsjy/jieba>

14. Zhu, Wenzhang. 2008. *Zhensu Bianzheng Xue (Syndrome Element Differentiation)*. Beijing: People’s Medical Publishing House.

15. Vaswani, Ashish, Noam Shazeer, Niki Parmar, Jakob Uszkoreit, Llion Jones, Aidan N Gomez, Łukasz Kaiser, Illia Polosukhin. 2017. “Attention is all you need.” *Advances in Neural Information Processing Systems 30*, pp. 5998–6008.

16. Cer, Daniel, Yinfei Yang, Sheng-yi Kong, Nan Hua, Nicole Limtiaco, Rhomni St John, Noah Constant, et al. 2018. “Universal sentence encoder.” *arXiv preprint arXiv:1803.11175*.

17. Chen, Ting, Simon Kornblith, Mohammad Norouzi, Geoffrey Hinton. 2020. “A simple framework for contrastive learning of visual representations.” *Proceedings of the 37th International Conference on Machine Learning*, pp. 1597–1607.

18. Hadsell, Raia, Sumit Chopra, Yann Lecun. 2006. “Dimensionality reduction by learning an invariant mapping.” *2006 IEEE Computer Society Conference on Computer Vision and Pattern Recognition*, pp. 1735–1742. <https://doi.org/10.1109/CVPR.2006.100>

19. Yao, Liang, Yin Zhang, Baogang Wei, Wenjin Zhang, Zhe Jin. 2018. “A topic modeling approach for Traditional Chinese Medicine prescriptions.” *IEEE Transactions on Knowledge and Data Engineering* 30: 1007–1021. <https://doi.org/10.1109/TKDE.2017.2787158>

20. Dong, Xin, Chenxi Zhao, Xinpeng Song, Lei Zhang, Yu Liu, Jun Wu, Yiran Xu, et al. 2024. “PresRecST: a novel herbal prescription recommendation algorithm for real-world patients with integration of syndrome differentiation and treatment planning.” *Journal of the American Medical Informatics Association* 31: 1268–1279. <https://doi.org/10.1093/jamia/ocae066>

21. Erosheva, Elena, Stephen Fienberg, John Lafferty. 2004. “Mixed-membership models of scientific publications.” *Proceedings of the National Academy of Sciences* 101: 5220–5227. <https://doi.org/10.1073/pnas.0307760101>

22. Nallapati, Ramesh, William W Cohen. 2008. “Link-PLSA-LDA: a new unsupervised model for topics and influence of blogs.” *Proceedings of the International AAAI Conference on Web and Social Media* 2: 84–92. <https://doi.org/10.1609/icwsm.v2i1.18621>

23. Yang, Yun, Yulong Rao, Minghao Yu, Yan Kang. 2022. “Multi-layer information fusion based on graph convolutional network for knowledge-driven herb recommendation.” *Neural Networks* 146: 1–10. <https://doi.org/10.1016/j.neunet.2021.11.010>

24. Dong, Xin, Yi Zheng, Zixin Shu, Kai Chang, Jianan Xia, Qiang Zhu, Kunyu Zhong, Xinyan Wang, Kuo Yang, Xuezhong Zhou. 2022. “TCMPR: TCM prescription recommendation based on subnetwork term mapping and deep learning.” *BioMed Research International* 2022: 4845726. <https://doi.org/10.1155/2022/4845726>

25. Zhong, Linda Li-Dan, Yi-Ping Wong, Chor-Yin Leung, Bo Peng, Zhi-Xiu Lin, Vivian Chi-Woon Wong Taam, Yi Luo, et al. 2022. “Effects of Chinese medicine for COVID-19 rehabilitation: a multicenter observational study.” *Chinese Medicine* 17: 99. <https://doi.org/10.1186/s13020-022-00654-z>

26. Luo, Jingyuan, Jialing Zhang, Hiu To Tang, Hoi Ki Wong, Aiping Lyu, Chun Hoi Cheung, Zhaoxiang Bian. 2023. “Prevalence and risk factors of long COVID 6–12 months after infection with the Omicron variant among nonhospitalized patients in Hong Kong.” *Journal of Medical Virology* 95: e28862. <https://doi.org/10.1002/jmv.28862>

27. Zhou, Xuezhong, Jörg Menche, Albert László Barabási, Amitabh Sharma. 2014. “Human symptoms-disease network.” *Nature Communications* 5: 4212. <https://doi.org/10.1038/ncomms5212>

28. U.S. National Library of Medicine. (November 19, 2022, date last accessed). Medical Subject Headings (MeSH). <https://www.ncbi.nlm.nih.gov/mesh>.

29. Baeza-Yates, Ricardo, Berthier Ribeiro-Neto. 1999. *Modern information retrieval*. New York: ACM Press.

30. U.S. National Library of Medicine. (January 7, 2024, date last accessed). MeSH Tree Structures. <https://www.nlm.nih.gov/mesh/intro_trees.html>.

31. Yan, Deyu, Genhui Zheng, Caicui Wang, Zikun Chen, Tiantian Mao, Jian Gao, Yu Yan, et al. 2022. “HIT 2.0: an enhanced platform for Herbal Ingredients’ Targets.” *Nucleic Acids Research* 50: D1238–D1243. <https://doi.org/10.1093/nar/gkab1011>

32. Menche, Jörg, Amitabh Sharma, Maksim Kitsak, Susan Dina Ghiassian, Marc Vidal, Joseph Loscalzo, Albert-László Barabási. 2015. “Uncovering disease-disease relationships through the incomplete interactome.” *Science* 347: 1257601. <https://doi.org/10.1126/science.1257601>

33. Amberger, Joanna S, Carol A Bocchini, François Schiettecatte, Alan F Scott, Ada Hamosh. 2015. “OMIM.org: Online Mendelian Inheritance in Man (OMIM®), an online catalog of human genes and genetic disorders.” *Nucleic Acids Research* 43: D789–D798. <https://doi.org/10.1093/nar/gku1205>

34. Sollis, Elliot, Abayomi Mosaku, Ala Abid, Annalisa Buniello, Maria Cerezo, Laurent Gil, Tudor Groza, et al. 2023. “The NHGRI-EBI GWAS Catalog: knowledgebase and deposition resource.” *Nucleic Acids Research* 51: D977–D985. <https://doi.org/10.1093/nar/gkac1010>

35. Morselli Gysi, Deisy, Ítalo do Valle, Marinka Zitnik, Asher Ameli, Xiao Gan, Onur Varol, Susan Dina Ghiassian, et al. 2021. “Network medicine framework for identifying drug-repurposing opportunities for COVID-19.” *Proceedings of the National Academy of Sciences* 118: e2025581118. <https://doi.org/10.1073/pnas.2025581118>

36. Mayo Clinic. (October 14, 2024, date last accessed). Diseases and Conditions. <https://www.mayoclinic.org/diseases-conditions>.

37. Guney, Emre, Jörg Menche, Marc Vidal, Albert László Barábasi. 2016. “Network-based *in silico* drug efficacy screening.” *Nature Communications* 7: 10331. <https://doi.org/10.1038/ncomms10331>

38. U.S. Food and Drug Administration. (December 25, 2024, date last accessed). openFDA Application Programming Interface. <https://open.fda.gov/apis/>.

39. Wishart, David S, Yannick D Feunang, An C Guo, Elvis J Lo, Ana Marcu, Jason R Grant, Tanvir Sajed, et al. 2018. “DrugBank 5.0: a major update to the DrugBank database for 2018.” *Nucleic Acids Research* 46: D1074–D1082. <https://doi.org/10.1093/nar/gkx1037>

40. Wei, Wei-Qi, Robert M Cronin, Hua Xu, Thomas A Lasko, Lisa Bastarache, Joshua C Denny. 2013. “Development and evaluation of an ensemble resource linking medications to their indications.” *Journal of the American Medical Informatics Association* 20: 954–961. <https://doi.org/10.1136/amiajnl-2012-001431>

41. Kim, Sunghwan, Jie Chen, Tiejun Cheng, Asta Gindulyte, Jia He, Siqian He, Qingliang Li, et al. 2019. “PubChem 2019 update: improved access to chemical data.” *Nucleic Acids Research* 47: D1102–D1109. <https://doi.org/10.1093/nar/gky1033>

42. Kane, Sean. (December 20, 2024, date last accessed). ClinCalc DrugStats Database. <https://www.clincalc.com/DrugStats/>.

43. Jaeger, Sabrina, Simone Fulle, Samo Turk. 2018. “Mol2vec: unsupervised machine learning approach with chemical intuition.” *Journal of Chemical Information and Modeling* 58: 27–35. <https://doi.org/10.1021/acs.jcim.7b00616>

44. Ashburner, Michael, Catherine A Ball, Judith A Blake, David Botstein, Heather Butler, J Michael Cherry, Allan P Davis, et al. 2000. “Gene Ontology: tool for the unification of biology.” *Nature Genetics* 25: 25–29. <https://doi.org/10.1038/75556>

45. Deng, Xiaohong, Siwei Zhang, Junzhen Wu, Xianjun Sun, Ziyin Shen, Jingcheng Dong, Jianhua Huang. 2019. “Promotion of mitochondrial biogenesis via activation of AMPK-PGC1ɑ signaling pathway by ginger (*Zingiber officinale* Roscoe) extract, and its major active component 6-gingerol.” *Journal of Food Science* 84: 2101–2111. <https://doi.org/10.1111/1750-3841.14723>

46. Li, Li, Manjing Jiang, Hong Wei, Linpan Liang, Yunlong Song, Dongni Xia, Qiang Luo, et al. 2026. “Dual regulation of antiviral IFN response by *Scutellariae Radix*: therapeutic implications for influenza.” *Journal of Pharmaceutical Analysis* 16: 101399. <https://doi.org/10.1016/j.jpha.2025.101399>

47. Luo, Xiaoping, Zhilun Yu, Chao Deng, Jingjing Zhang, Gaiyan Ren, Aning Sun, Sridhar Mani, Zhengtao Wang, Wei Dou. 2017. “Baicalein ameliorates TNBS-induced colitis by suppressing TLR4/MyD88 signaling cascade and NLRP3 inflammasome activation in mice.” *Scientific Reports* 7: 16374. <https://doi.org/10.1038/s41598-017-12562-6>

48. Kobayashi, Yukihiro, Tetsuya Hiroi, Mayumi Araki, Takahiko Hirokawa, Maki Miyazawa, Nobuyoshi Aoki, Takashi Kojima, Toshiyuki Ohsawa. 2012. “Facilitative effects of *Eucommia ulmoides* on fatty acid oxidation in hypertriglyceridaemic rats.” *Journal of the Science of Food and Agriculture* 92: 358–365. <https://doi.org/10.1002/jsfa.4586>

49. Hao, Shun, Yuan Xiao, Yan Lin, Zhentao Mo, Yang Chen, Xiaofeng Peng, Canhui Xiang, Yiqi Li, Wenna Li. 2016. “Chlorogenic acid-enriched extract from *Eucommia ulmoides* leaves inhibits hepatic lipid accumulation through regulation of cholesterol metabolism in HepG2 cells.” *Pharmaceutical Biology* 54: 251–259. <https://doi.org/10.3109/13880209.2015.1029054>

50. Chen, Jianping. 2006. *Fang Ji Xue (Prescription of Chinese Medicine)*. International ed. Hong Kong: Commercial Press HK.

51. Mu, Nan, Jintao Gu, Tonglie Huang, Cun Zhang, Zhen Shu, Meng Li, Qiang Hao, et al. 2016. “A novel NF-κB/YY1/microRNA-10a regulatory circuit in fibroblast-like synoviocytes regulates inflammation in rheumatoid arthritis.” *Scientific Reports* 6: 20059. <https://doi.org/10.1038/srep20059>

52. Mun, Yoonwoo, Woohyun Kim, Dongyun Shin. 2023. “Melanocortin 1 receptor (MC1R): pharmacological and therapeutic aspects.” *International Journal of Molecular Sciences* 24: 12152. <https://doi.org/10.3390/ijms241512152>

53. Qin, Jiahuan, Xia Huang, Naiqi Wang, Pengcheng Zhou, Hao Zhang, Zhian Chen, Kaili Liang, et al. 2021. “Supranutritional selenium suppresses ROS-induced generation of RANKL-expressing osteoclastogenic CD4+ T cells and ameliorates rheumatoid arthritis.” *Clinical & Translational Immunology* 10: e1338. <https://doi.org/10.1002/cti2.1338>

54. Sial, Nabeela Tabassum, Abdul Malik, Urooj Iqbal, Muhammad Fayyaz ur Rehman. 2024. “Arbutin attenuates CFA-induced arthritis by modulating expression levels of 5-LOX, NF‑κB, IL-17, PGE-2 and TNF-α.” *Inflammopharmacology* 32: 2377–2394. <https://doi.org/10.1007/s10787-024-01480-5>

55. Yang, Ruo-yan, Kun Liu, Jian-ying Wang, Lei Zhang, Bao-jun Zhang, Ying Yuan. 2023. “Pharmacokinetics, anti-rheumatoid arthritis activity, and active ingredient contents of *Eucommia ulmoides* Oliv.” *Fitoterapia* 170: 105667. <https://doi.org/10.1016/j.fitote.2023.105667>

56. Chen, Wen-jin, Yin Zhuang, Wei Peng, Wei Cui, Shu-jun Zhang, Jian-wei Wang. 2023. “Du Huo Ji Sheng Tang inhibits Notch1 signaling and subsequent NLRP3 activation to alleviate cartilage degradation in KOA mice.” *Chinese Medicine* 18: 80. <https://doi.org/10.1186/s13020-023-00784-y>

57. Gu, Lili, Yuling Sun, Ting Wu, Ge Chen, Xiaojun Tang, Lianfeng Zhao, Lingfeng He, et al. 2022. “A novel mechanism for macrophage pyroptosis in rheumatoid arthritis induced by Pol β deficiency.” *Cell Death & Disease* 13: 583. <https://doi.org/10.1038/s41419-022-05047-6>

58. Petersson, Maria, Elisabet Bucht, Barbro Granberg, André Stark. 2006. “Effects of arginine-vasopressin and parathyroid hormone-related protein (1–34) on cell proliferation and production of YKL-40 in cultured chondrocytes from patients with rheumatoid arthritis and osteoarthritis.” *Osteoarthritis and Cartilage* 14: 652–659. <https://doi.org/10.1016/j.joca.2006.01.003>

59. Ait Eldjoudi, Djedjiga, Alfonso Cordero Barreal, María Gonzalez-Rodríguez, Clara Ruiz-Fernández, Yousof Farrag, Mariam Farrag, Francisca Lago, et al. 2022. “Leptin in osteoarthritis and rheumatoid arthritis: player or bystander?” *International Journal of Molecular Sciences* 23: 2859. <https://doi.org/10.3390/ijms23052859>

60. Murayama, Masanori A, Jun Shimizu, Chie Miyabe, Kazuo Yudo, Yoshishige Miyabe. 2023. “Chemokines and chemokine receptors as promising targets in rheumatoid arthritis.” *Frontiers in Immunology* 14: 1100869. <https://doi.org/10.3389/fimmu.2023.1100869>

61. Yang, Daishui, Tang Liu, Guangyao Jiang, Xuantao Hu, Tao Zheng, Tao Li, Zhi Gao, Zhengxiao Ouyang, Baoyu Zhu. 2020. “Senkyunolide H attenuates osteoclastogenesis and postmenopausal osteoporosis by regulating the NF-κB, JNK and ERK signaling pathways.” *Biochemical and Biophysical Research Communications* 533: 510–518. <https://doi.org/10.1016/j.bbrc.2020.09.054>

62. Feng, Yanbin, Yilin Teng, Yaomin Mo, Deshuang Xi, Junhong Zhou, Gaofeng Zeng, Shaohui Zong. 2024. “Unveiling the potential of butylphthalide: inhibiting osteoclastogenesis and preventing bone loss.” *Frontiers in Pharmacology* 15: 1347241. <https://doi.org/10.3389/fphar.2024.1347241>

63. Yu, Huan, Sai Yao, Chengchong Zhou, Fangda Fu, Huan Luo, Weibin Du, Hongting Jin, et al. 2021. “Morroniside attenuates apoptosis and pyroptosis of chondrocytes and ameliorates osteoarthritic development by inhibiting NF-κB signaling.” *Journal of Ethnopharmacology* 266: 113447. <https://doi.org/10.1016/j.jep.2020.113447>

64. Khansai, Manatsanan, Kanchanit Boonmaleerat, Peraphan Pothacharoen, Thanyaluck Phitak, Prachya Kongtawelert. 2016. “Ex vivo model exhibits protective effects of sesamin against destruction of cartilage induced with a combination of tumor necrosis factor-alpha and oncostatin M.” *BMC Complementary and Alternative Medicine* 16: 205. <https://doi.org/10.1186/s12906-016-1183-0>

65. Hu, Jiaming, Jinyi Zhou, Jinting Wu, Quan Chen, Weibin Du, Fangda Fu, Huan Yu, et al. 2020. “Loganin ameliorates cartilage degeneration and osteoarthritis development in an osteoarthritis mouse model through inhibition of NF-κB activity and pyroptosis in chondrocytes.” *Journal of Ethnopharmacology* 247: 112261. <https://doi.org/10.1016/j.jep.2019.112261>

66. Liu, Qian, Jinmin Zhao, Ren-Xiang Tan, Hua Zhou, Zhen Lin, Minghao Zheng, Evange Romas, Jiake Xu, Natalie A. Sims. 2015. “Parthenolide inhibits pro-inflammatory cytokine production and exhibits protective effects on progression of collagen-induced arthritis in a rat model.” *Scandinavian Journal of Rheumatology* 44: 182–191. <https://doi.org/10.3109/03009742.2014.938113>

67. Yuan, Jia-huan, Zhi-chen Cai, Cui-hua Chen, Nan Wu, Sheng-xin Yin, Wen-xin Wang, Hai-jie Chen, Yong-yi Zhou, Li Li, Xun-hong Liu. 2022. “A study for quality evaluation of *Taxilli Herba* from different hosts based on fingerprint-activity relationship modeling and multivariate statistical analysis.” *Arabian Journal of Chemistry* 15: 103933. <https://doi.org/10.1016/j.arabjc.2022.103933>

68. Javed, Komal, Allah Rakha, Masood Sadiq Butt, Muhammad Naeem Faisal, Urwa Tariq, Makkia Saleem. 2022. “Evaluating the anti-arthritic potential of walnut (*Juglans regia* L.) in FCA induced Sprague Dawley rats.” *Journal of Food Biochemistry* 46: e14327. <https://doi.org/10.1111/jfbc.14327>

69. Fu, Jun, Huan Wu, Hong Wu, Ran Deng, Minghui Sun. 2021. “Deciphering the metabolic profile and pharmacological mechanisms of *Achyranthes bidentata* Blume saponins using ultra-performance liquid chromatography quadrupole time-of-flight mass spectrometry coupled with network pharmacology-based investigation.” *Journal of Ethnopharmacology* 274: 114067. <https://doi.org/10.1016/j.jep.2021.114067>

70. Jiang, Si-Qi, Ting Pan, Jia-Lin Yu, Ying Zhang, Ting Wang, Ping Li, Fei Li. 2022. “Thermal and wine processing enhanced *Clematidis Radix et Rhizoma* ameliorate collagen Ⅱ induced rheumatoid arthritis in rats.” *Journal of Ethnopharmacology* 288: 114993. <https://doi.org/10.1016/j.jep.2022.114993>

71. Han, Sang‑Yong, Kyung‑Hee Lee, Yun‑Kyung Kim. 2018. “*Poligoni Multiflori Radix* enhances osteoblast formation and reduces osteoclast differentiation.” *International Journal of Molecular Medicine* 42: 331–345. <https://doi.org/10.3892/ijmm.2018.3603>

72. Biggs, Isaac, Joseph Sirdaarta, Alan White, Ian Edwin Cock. 2016. “GC-MS analysis of *Commiphora molmol* oleo-resin extracts which inhibit the growth of bacterial triggers of selected autoimmune diseases.” *Pharmacognosy Journal* 8: 191–202. <https://doi.org/10.5530/pj.2016.3.4>

73. Ni, Hui-Ying, Yao-Ping Zhang, Xiao-Feng Zhang. 2024. “Therapeutic effect of San Bi Tang combined with glucosamine sulfate capsules in cold-dampness-type knee osteoarthritis.” *World Journal of Clinical Cases* 12: 3854–3865. <https://doi.org/10.12998/wjcc.v12.i19.3854>

74. Qi, Xiao-Yu, Hao Liu, Dong-Dong Bi, Xiang-Tao Wang, Yi-Fei Guo, Ting Hao, Bao-Xin Zhang, Xing-Guo Wang, Mei-Hua Han. 2019. “Combined administration on You-Gui Yin and low-dose raloxifene partially attenuates the bone loss in ovariectomized mice through the proliferation and osteogenic differentiation of bone marrow stromal cells.” *Phytomedicine* 53: 286–293. <https://doi.org/10.1016/j.phymed.2018.09.014>

75. Wang, Tengteng, Qingyun Jia, Tao Chen, Hao Yin, Xiaoting Tian, Xi Lin, Yang Liu, et al. 2020. “Alleviation of synovial inflammation of Juanbi-Tang on collagen-induced arthritis and TNF-Tg mice model.” *Frontiers in Pharmacology* 11: 45. <https://doi.org/10.3389/fphar.2020.00045>

76. Zhang, Lei, Ping-er Wang, Jun Ying, Xing Jin, Cheng Luo, Taotao Xu, Shibing Xu, et al. 2017. “Yougui pills attenuate cartilage degeneration via activation of TGF-β/Smad signaling in chondrocyte of osteoarthritic mouse model.” *Frontiers in Pharmacology* 8: 611. <https://doi.org/10.3389/fphar.2017.00611>

77. Yang, Shuo, Bin Zhang, Yu-guo Wang, Zi-wei Liu, Bo Qiao, Juan Xu, Li-sheng Zhao. 2023. “Zuo Gui Wan promotes osteogenesis via PI3K/AKT signaling pathway: network pharmacology analysis and experimental validation.” *Current Medical Science* 43: 1051–1060. <https://doi.org/10.1007/s11596-023-2782-x>

# SUPPORTING FIGURES

## Figure S1 Conceptual framework and empirical diagnostic–therapeutic relationships in historical TCM records.

(A) Schematic comparison of a reductionist biomedical framework (upper path) and the TCM holistic framework (lower path). The former expands from clinical phenotypes toward molecular-level descriptions, whereas the latter relates symptom patterns to herbal therapies through conventional TCM diagnostic and therapeutic principles.

(B) Visualization of symptom–syndrome–herb relationships recorded in ancient and classical TCM formula records. Symptoms, differentiated syndromes, and prescribed herbs are represented by squares, triangles, and circles, respectively. For visualization of pairwise relationships, edge weights were quantified using normalized pointwise mutual information (NPMI). Nodes for herbs and syndromes are colour-coded according to conventional TCM annotations, distinguishing herb natures and syndrome pathogenesis. An example formula record, Ren-Shen-Ban-Xia-Wan, is highlighted to illustrate the diagnostic–therapeutic relationships linking symptoms, syndromes, and prescribed herbs.

## Figure S2 Frequency distributions and corpus coverage of TCM herbs, symptoms and syndromes.

(A-C) Frequency distributions of standardized TCM symptoms (A), syndromes (B), and herbs (C), respectively. Frequencies are shown on a logarithmic scale.

(D) Effect of the minimum herb-frequency threshold on the number of retained herbs and the proportion of formula records that can be represented entirely using the retained vocabulary. A minimum frequency of 25 retained 780 herbs and allowed complete representation of 75,976 of 84,491 formula records (89.9%).

(E) Cumulative herb-occurrence coverage as a function of retained vocabulary size. Herbs were ranked by corpus frequency. The top 780 herbs, corresponding to a minimum frequency of 25, accounted for 98.9% of all herb occurrences.

## Figure S3 The model for the TCM-ES construction and biomedical entities integration.

Step 1: Core model development using 26,339 model-eligible TCM formula records. The model uses an autoencoder architecture with two encoders and two decoders, each consisting of four multi-head attention blocks with eight heads. For each record, the input symptom pattern is represented as a sequence of semantic vectors generated using the pretrained Google Universal Sentence Encoder (GUSE), and the input formula is represented as a sequence of one-hot vectors indicating its herbal composition. The symptom-pattern and formula inputs are processed through Encoder 1 and Encoder 2, respectively, and the resulting token embeddings are averaged to generate symptom-pattern and formula embeddings. Embeddings of matched symptom-pattern–formula pairs from the same record are aligned via contrastive loss. These embeddings are then passed through Decoder 1 and Decoder 2 to reconstruct the corresponding input sequences. The total training loss combines contrastive loss, which minimizes the distance between matched pairs, and reconstruction loss, which minimizes discrepancies between the inputs and their reconstructions.

Step 2: Mapping TCM entities into TCM-ES. After training, symptoms and symptom patterns are mapped into TCM-ES using the fixed Encoder 1, while herbs and formulas are mapped using the fixed Encoder 2.

Step 3: Mapping biomedical entities into the fixed TCM-ES. Human diseases are represented by their associated symptom patterns and mapped into TCM-ES using Encoder 1. Two additional autoencoders (Encoders/Decoders 3 and 4) were trained to map herbal compounds, represented by chemical-structure vectors, and target proteins, represented by one-hot vectors, into proximity with their associated herbs in TCM-ES. Similar to the main model, these two autoencoders were optimized using contrastive loss to align compounds or targets with their associated herbs and reconstruction loss to preserve their input representations. After training, modern drugs within the supported chemical domain were mapped using the trained Encoder 3 based on their chemical-structure representations. The core TCM-ES remained fixed throughout biomedical entity mapping.

## Figure S4 Loss curves during main model training.

Reconstruction loss (top), contrastive loss (middle), and total loss (bottom) are calculated for the training set (solid lines) and the validation set (dashed lines) at each training epoch. Because dropout and data augmentation are applied only to the training data, the training reconstruction loss remains slightly higher than the corresponding validation loss. Reconstruction loss decreases and then stabilizes, whereas contrastive loss decreases initially and subsequently increases, indicating the onset of over-fitting. The final model was selected before the total loss began to rise, corresponding to the 60th epoch.

## Figure S5 Design of the TCM-ES ablation models.

The full TCM-ES model contains modality-specific symptom and herb encoders, bidirectional cross-attention modules, reconstruction heads, and a contrastive objective aligning the pooled symptom and herb embeddings. Three controlled ablation variants were constructed while keeping the remaining architecture and training workflow unchanged. In the no-attention model, self-attention and cross-attention operations were removed, while the surrounding residual, normalization, feed-forward, pooling, and output-head structure was retained. In the no-contrastive model, the full architecture was preserved but the contrastive weight was set to zero, leaving only the reconstruction objective. In the shuffled-semantic model, the architecture and objective were unchanged, but the pretrained symptom semantic vectors were randomly reassigned among symptom identities. All models used the same training procedure and were trained on the same non-leakage split described in Note S3.

## Figure S6 Distinct contributions of attention, pretrained symptom semantics, and contrastive learning to TCM-ES representation structure.

The full TCM-ES model was compared with the no-attention, shuffled-semantic, and no-contrastive ablation models trained on the same non-leakage split.

(A-C) Validation total (A), reconstruction (B), and contrastive (C) losses at the checkpoint with the minimum validation total loss for each training seed. These panels compare only the full, no-attention, and shuffled-semantic models, which were trained with the same reconstruction-plus-contrastive objective; the no-contrastive model was excluded because its training objective differed. Points show individual training seeds.

(D-F) Syndrome-aligned neighbourhood consistency at *k* = 20 in the symptom-pattern embedding (D), formula embedding (E), and cross-modal (F) spaces. Neighbourhood consistency was defined as the proportion of nearest neighbours sharing at least one TCM syndrome label with the query record.

(G-I) Sensitivity of the same measures to neighbourhood size (*k* = 5, 10, 20, 50, and 100).

(J and K) Direct cross-modal retrieval. Held-out symptom-pattern embeddings were used to retrieve their matched formula embeddings (J), and formula embeddings were used to retrieve their matched symptom-pattern embeddings (K). For each query, the candidate pool contained the matched embedding and 49 randomly sampled unmatched candidates. Candidate sampling was repeated 100 times using the same candidate sets across models. Points show the mean Hit@1, Hit@5, and Hit@10 across independently trained seeds, with error bars indicating the standard deviation across seeds. Grey dashed lines indicate the corresponding random-ranking expectations for 50 candidates.

## Figure S7 Attention patterns for TCM formula records and disease-associated symptom patterns.

(A-D) Attention patterns for the classical TCM formula record Si-Jun-Zi-Tang, consisting of five symptoms and four herbs. Self-attention weights from Encoder 1 and Encoder 2 (A-B) summarize context-dependent weighting among symptom or herb tokens within each modality, whereas cross-attention weights from Decoder 1 and Decoder 2 (C-D) summarize the weighting of tokens from one modality during reconstruction of the other modality. Larger heatmap values indicate greater model-assigned attention to the corresponding token under the specific input context. The two cross-attention directions are conditioned on different input contexts and are therefore not expected to be symmetric. Attention weights were averaged across attention heads. In this example, Ren-Shen received relatively high attention weights in several symptom–herb and herb–herb relationships.

(E-H) Corresponding attention patterns for the TCM formula record Xuan-Fei-San. Although Ren-Shen was also present in this formula, its attention-weight pattern differed from that observed for Si-Jun-Zi-Tang, illustrating the context dependence of the learned attention weights.

(I-K) Attention patterns for the symptom representations of three example diseases located near one another in TCM-ES. The disease-associated symptom patterns showed different attention-weight distributions, illustrating that the model assigns context-dependent weights to constituent symptoms.

## Figure S8 Attention-guided perturbation of symptom-pattern–formula reconstruction in held-out formula records.

For each symptom-pattern–formula pair in the held out test set, symptoms and herbs were ranked using cross-attention scores from the final cross-attention layer. The attention score of each token was defined as its mean attention weight across heads and across tokens in the opposite modality. For example, a symptom received a higher score when formula tokens (i.e., herbs) assigned greater average attention to that symptom during formula reconstruction. One, two, or three top-attention, randomly selected, or low-attention tokens were then removed, and the frozen model was rerun.

(A) Increase in formula reconstruction negative log-likelihood (NLL) after symptom removal.

(B) Increase in symptom pattern reconstruction NLL after herb removal. Points show mean reconstruction degradation and error bars show 95% confidence intervals obtained from 1000 bootstrap resamples of eligible records. Random removal was repeated ten times and averaged within each record. High-attention removal was compared with random and low-attention removal using one-sided paired Wilcoxon signed-rank tests with Benjamini–Hochberg correction.

## Figure S9 Dimensions of the embeddings.

(A) Minimum validation loss values (including contrastive loss, reconstruction loss, and their average values) achieved by models with embedding dimensions (the number of neurons in the embedding layer) ranging from 8 to 1024. Error bars indicate the standard deviation across five repeated trials for each embedding dimension. The minimum-loss epoch was determined as the training epoch achieving the lowest total validation loss. Minimum losses were computed as the average loss over five consecutive epochs centred around the minimum-loss epoch. All models shared the same architecture and hyperparameters, differing only in the embedding dimensions. The minimum losses achieved by models with different embedding dimensions converge when the dimension exceeds approximately 250.

(B) Principal-component analysis (PCA) of record-level TCM-ES embeddings from the original training set. Bars show the variance explained by individual principal components and the line shows cumulative explained variance, illustrating that most variation was concentrated in a small number of leading components.

(C) PCA of the corresponding raw record representations, constructed by concatenating symptom-pattern and formula multi-hot vectors. Variance was distributed across substantially more principal components than in the TCM-ES embeddings.

## Figure S10 Normalization of the embedding distance.

(A) The histogram shows the L2-norm distribution of embedding vectors of all symptom patterns and formulas in the model training set. The red vertical line marks the 95th percentile threshold of L2-norms.

(B) Distribution of normalized distances among all symptom patterns and formulas. The normalized distances were calculated by scaling original Euclidean distances with twice the 95th percentile threshold. Distances are concentrated in the range $\text{[0, 1]}$, with values below 0.15 indicating proximity and values above 0.6 indicating large distance.

## Figure S11 Principal TCM-ES results are retained after retraining with the non-leakage split.

TCM-ES was retrained from random initialization using the conservative non-leakage split defined in Table S3.

(A) Principal-component spectrum obtained by fitting PCA to the averaged symptom-pattern and formula embeddings of the training records from the conservative split. The first six components captured more than 95% of the variance.

(B and C) Syndrome-associated organization of symptom-pattern (B) and formula (C) embeddings generated by the retrained model in the corresponding PCA space. Syndrome annotations were not used for model training and were used only for visualization.

(D) Held-out formula prediction on the test set from the conservative split, comparing MLP and linear ridge readouts from TCM-ES symptom-pattern embeddings with a ridge model using raw symptom multi-hot vectors and a ridge model using shuffled TCM-ES embeddings.

(E-G) External formula prediction on the TCM-PD benchmark using the frozen encoder retrained with the conservative non-leakage split, shown as Precision@*k* (E), Recall@*k* (F), and F1@*k* (G). The held-out and external formula prediction tasks otherwise followed the same procedures described in Note S4.

## Figure S12 Global and local stability of TCM-ES geometry across repeated training.

Ten TCM-ES models were independently trained using different data splits, and the validation-optimal checkpoint from each repeat was used to embed the same set of formula records.

(A) Global stability measured by the Spearman correlation between pairwise similarity values obtained from 200,000 common record pairs in each pair of repeat models.

(B) Local robustness measured for each record as the mean Jaccard overlap of its 200-nearest-neighbour sets across all 45 repeat-model pairs. Local-stability values are displayed on the PC1-PC3 coordinates of the original model for visualization only.

## Figure S13 Alignment of principal components across repeated TCM-ES models.

PCA was fitted independently to the main model and each of ten independently trained repeat models using the same aligned set of formula records. For each record, the record-level embedding was the average of its symptom-pattern and formula embeddings.

(A-J) Pearson correlations between the first six main-model PC-score vectors and the first six PC-score vectors from each repeat model. Because PCA axis order and sign are arbitrary, repeat-model PCs were matched one-to-one to the main-model PCs using the Hungarian algorithm to maximize the total absolute PC-score correlation. Matched repeat PCs with negative correlations were sign-reversed before downstream analyses. The leading PCs showed strong one-to-one correspondence across repeats.

## Figure S14 Quantitative characterization and robustness of syndrome-associated organization in TCM-ES.

(A) Point-biserial correlations were calculated between the activation of each of the 256 latent dimensions and the presence of the 40 most frequent TCM syndrome annotations across 7098 formula records. Syndrome annotations were excluded from model training and were introduced only after training for evaluation. Latent dimensions were ordered by hierarchical clustering of their syndrome-associated activation profiles, revealing a block-wise correspondence between groups of latent dimensions and syndrome annotations. To quantify this correspondence, syndrome–syndrome Euclidean distance matrices were constructed separately from the conventional symptom–herb NPMI profiles and the 256-dimensional latent association profiles, and their upper-triangular elements were compared using Spearman correlation. The correspondence was strong (*ρ* = 0.810) and exceeded that obtained after 10,000 syndrome-identity permutations (Holm-adjusted two-sided *p*_perm_ = 0.0002).

(B) The same syndrome–dimension relationships were recalculated using standardized mean activation scores instead of point-biserial correlations. For each latent dimension, the mean activation among records annotated with each syndrome was standardized relative to that dimension’s activation across all evaluated records. A similar block-wise pattern was retained. Using standardized mean activation instead of point-biserial correlation yielded a similarly strong correspondence (*ρ* = 0.769; Holm-adjusted two-sided *p*_perm_ = 0.0002).

(C) Hierarchical clustering was then applied independently to both the syndrome annotations and latent dimensions using the standardized activation matrix, again recovering a similar organization. Together, these analyses show that the syndrome-associated activation pattern is reproducible across alternative measures of latent activation and clustering procedures, supporting a distributed syndrome-associated organization in TCM-ES rather than a one-to-one correspondence between individual dimensions and specific syndromes. Because both axes were clustered from the same activation matrix, panel C served as a clustering-robustness analysis rather than an independent correspondence test.

(D) Held-out linear readout of syndrome annotations from frozen TCM-ES embeddings. One-vs-rest logistic-regression classifiers were fitted using the predefined training records and evaluated on the held-out test records. Mean ROC curves are shown across 66 evaluable syndrome labels for symptom-pattern and formula embeddings; shaded regions indicate pointwise 95% bootstrap intervals from 1000 resamples of the test records. Macro-AUROC was 0.844 (95% bootstrap CI, 0.818-0.863) for symptom-pattern embeddings and 0.851 (0.830-0.869) for formula embeddings. The dashed diagonal indicates random discrimination (AUROC = 0.5).

## Figure S15 Additional conventional TCM annotations are associated with the organization of TCM-ES.

(A) Formula embeddings were visualized according to their normalized cold–hot scores, calculated from the conventional TCM natures of their constituent herbs. A continuous cold–hot-associated gradient was observed across TCM-ES.

(B) Formula embeddings were coloured according to their dominant herb channel, defined as the most frequent conventional channel attribution among the herbs within each formula. Formulas with different dominant channels occupied partially differentiated but overlapping regions of TCM-ES. Herb-nature and herb-channel annotations were not used for model training and were introduced only after training for these analyses. These patterns provide additional evidence that the learned TCM-ES organization is associated with multiple conventional TCM annotations beyond syndrome labels.

## Figure S16 ****Hierarchical explanatory factors of embedding geometry.****

For each sampled pair of formula records *i* and *j*, each containing a symptom pattern and an herbal formula, embedding proximity was defined as the negative Euclidean distance between their symptom-pattern embeddings, or between their formula embeddings. Larger values therefore indicate greater embedding proximity. **Five nested factor sets that potentially explain variation in embedding proximity were evaluated. Conceptually, *M*1*-M*5 progress from simple within-modality similarity to increasingly higher-order cross-modal structure.**

***M*1** included literal set overlap and within-domain co-occurrence in the same modality as the embedding proximity being explained (**e.g., symptom overlap and symptom–symptom co-occurrence when explaining symptom-embedding proximity**). ***M*2** additionally included overlap and within-domain co-occurrence from the counterpart modality (e**.g., similarity between the corresponding herbal formulas when explaining symptom-embedding proximity**). ***M*3** added symptom–herb co-occurrence for both the observed symptom-pattern–formula pairings and the crossed pairings between the two records (**e.g., symptoms from record** $\text{i}$ **with herbs from either formula *i*** **or formula *j***). ***M*4** further added compatibility between high-confidence symptom-pair and herb-pair co-occurrence structures, asking whether a symptom-pair pattern and a herb-pair pattern tended to show similar neighbourhoods across the training records. ***M*5** extended this idea from selected item pairs to the complete symptom pattern and formula, asking whether a whole symptom pattern and a whole formula showed similar Jaccard-neighbourhood profiles over the same training records. The co-occurrence reference records comprised the training set used to train the main model and to estimate all co-occurrence factors.

**For each of the five nested factor sets, regression models were fitted to predict pairwise embedding proximity from the corresponding explanatory factors.**

**(A and B)** Standardized ordinary least-squares models were used to quantify the linear association between these factors and symptom-pattern embedding proximity (**A**) or formula embedding proximity (**B**). The panels show the corresponding in-sample *R*^2^.

**(C and D)** To assess whether the same factor sets could explain embedding proximity under a more flexible nonlinear model and out-of-sample evaluation, gradient-boosting regressors were fitted for symptom-pattern (**C**) and formula (**D**) embedding proximity. The panels show the corresponding out-of-sample *R*^2^ in held-out set.

In each panel the original TCM-ES is compared with the shuffled-pairing and independent-modality null models. Each analysis was repeated across 20 independently sampled sets of 200,000 unique test-record pairs. Points and error bars show the mean and standard deviation across the 20 pair-sampling repeats. **Across both the linear and nonlinear analyses, the clearest progressive increase in explanatory power from *M*1 to *M*5 was observed for the original TCM-ES.** The later increments were markedly weakened in the two null models, and this distinction was reproduced for both symptom-pattern and formula embeddings**.**

## Figure S17 Principal-component analysis of the original TCM-ES, null models, and co-occurrence-based baseline embeddings.

Principal component analysis (PCA) was fitted separately to the record-level embeddings shown in each panel.

**(A)** Original TCM-ES, using the averaged symptom-pattern and formula embeddings of each record.

**(B)** GUSE semantic baseline, using the symptom-pattern embedding obtained by averaging the pretrained GUSE semantic vectors of the symptoms in each record, without TCM-ES training.

**(C)** Independent-modality null model, using the record-formula embedding; the two modalities were not averaged because this null model did not constrain symptom-pattern and formula embeddings to occupy a shared aligned space.

**(D)** Shuffled-pairing null model, using the averaged symptom-pattern and formula embeddings.

**(E and F)** Co-occurrence SVD (**E**) and graph (**F**) baselines, using the averaged symptom-pattern and formula embeddings derived from their respective shared co-occurrence spaces. Bars show the proportion of variance explained by each principal component, and lines show cumulative explained variance. Horizontal dashed lines denote 95% cumulative variance, and vertical dotted lines indicate the corresponding number of principal components. The original TCM-ES showed a compact but multiaxial spectrum, with approximately 95% of the record-level variance captured by the first six principal components and distributed across several leading dimensions. In contrast, the GUSE semantic baseline and the two null models showed much more diffuse spectra. The co-occurrence SVD baseline was highly concentrated in PC1, whereas the graph baseline retained a broader spectrum and required substantially more components than TCM-ES to reach 95% cumulative variance.

## Figure S18 Syndrome-associated organization of formula record embeddings across semantic-only, null, and co-occurrence-based baseline embeddings.

Formula records are projected onto the first three principal components. Principal component analysis (PCA) was fitted independently for each model. Each point represents one record and is coloured according to its syndrome label.

**(A)** GUSE semantic-only baseline using symptom-pattern embeddings.

**(B and C),** Independent-modality null model using symptom-pattern (**B**) and formula (**C**) embeddings.

**(D and E)** Shuffled-pairing null model using symptom-pattern (**D**) and formula (**E**) embeddings.

**(F and G)** Co-occurrence SVD baseline using symptom-pattern (**F**) and formula (**G**) embeddings.

**(H and I)** Co-occurrence graph baseline using symptom-pattern (**H**) and formula (**I**) embeddings.

For the original TCM-ES, shuffled-pairing null model, and the two co-occurrence baselines, PCA was fitted to the average of symptom-pattern and formula embeddings, and the corresponding symptom and formula embeddings were projected into the same model-specific PCA space. For the independent-modality null model, the symptom-pattern and formula embeddings were not constrained to occupy a shared aligned space and were therefore analysed using separately fitted PCA spaces. For the GUSE semantic-only baseline, PCA was fitted to the symptom-pattern embeddings only. The original TCM-ES showed more broadly corresponding syndrome-associated organization in the symptom-pattern and formula embeddings.

## Figure S19 Examples of formula decoding based on symptom patterns using the trained model.

Three examples from the test set are presented to demonstrate the model’s ability to predict (decode) formulas. For each formula record, given the symptom pattern, the model decodes herbs sequentially using the trained herb decoder (Decoder 2), and the predicted herbs are compared to the original herbal compositions of the formula (ground truth), with overlapping herbs highlighted in green. (A) In this example, the model accurately predicts all herbs of the classical formula Si-Jun-Zi-Tang. (B) In this example, the model predicts four out of seven herbs in Qiang-Huo-Sheng-Shi-Tang. The four predicted herbs, especially Qiang-Huo (Notopterygii Rhizoma et Radix), play central roles in the formula. (C) In this example, the model predicts the core herb Shi-Gao (Gypsum Fibrosum), though the remaining predicted herbs differ significantly from the original formula. However, the diversity of formulas for the same symptom pattern makes it challenging to use the prescribed formula as the sole ground truth for model evaluation. These examples are provided as qualitative illustrations of the decoder output. Quantitative evaluation of formula prediction performance is presented separately in Figure S20.

## Figure S20 Held-out formula prediction from symptom patterns.

Formula prediction performance was evaluated on held-out test set of the TCM formula records using frozen TCM-ES symptom-pattern embeddings. Linear ridge and MLP readouts were compared with a ridge model using raw symptom multi-hot input and a ridge model using shuffled TCM-ES embeddings. Performance was evaluated using Precision@*k*, Recall@*k*, and F1@*k*. Both TCM-ES readouts consistently outperformed the raw-input and shuffled-embedding controls, while the MLP provided only modest improvement over the linear ridge readout.

## Figure S21 Patient characteristics and symptom distributions in the clinical datasets.

(A-F) For general TCM cases, distributions of age (A), sex (B), education level (C), occupation (D), ICD-10 disease classes (E), and the 20 most frequently recorded symptoms (F) are shown.

(G-I) For long-COVID cases, distributions of age (G), sex (H), and the 20 most frequently recorded symptoms (I) are shown.

(J-L) For COVID-19 cases, distributions of age (J), sex (K), and the 20 most frequently recorded symptoms (L) are shown. These clinical cases span a broad population with diverse characteristics and symptoms.

## Figure S22 Prescription frequencies of herbs and standard formulas in the clinical datasets.

(A) Frequencies of the 20 most commonly prescribed herbs in general TCM clinical cases.

(B) Frequencies of the 20 most commonly prescribed herbs in long COVID cases.

(C) Prescription frequency of the nine standard formulas in the COVID-19 cases.

## Figure S23 Semantic coverage of symptom terms used for biomedical and clinical mapping.

GUSE semantic embeddings of standardized TCM symptoms, modern-medicine disease-associated symptoms, and COVID-19 symptoms were visualized using UMAP. The broad overlap illustrates that symptom terms used for downstream biomedical and clinical mapping occupy a broadly shared semantic domain with the TCM symptom vocabulary used to construct TCM-ES.

## Figure S24 Consistency of TCM-ES geometry with observed clinical and TCM practice relationships.

(A) As a basic empirical consistency check, symptom–symptom embedding distances were compared with symptom co-occurrence patterns observed in general TCM clinical cases, long-COVID cases, and disease-associated symptom data. Pairwise co-occurrence was quantified using the Jaccard index.

(B) Herb–herb embedding distances were similarly compared with herb co-prescription patterns observed in general TCM and long-COVID clinical cases. In A and B, closer embeddings were associated with more frequently observed co-occurrence or co-prescription relationships.

(C) Herb–symptom embedding distances were compared between indication pairs documented in the Chinese Pharmacopoeia and non-indicated pairs; documented pairs showed shorter embedding distances.

(D) Exploratory retrospective analysis of herb-pair interaction patterns in general TCM clinical cases. For each eligible herb–herb–symptom triplet, the relative change in symptom proximity for the jointly represented herb pair versus the individual herbs was compared with the corresponding difference in observed symptom-recovery outcomes. Herb pairs showing relatively closer joint proximity together with more favourable paired outcomes were defined as showing a positive interaction pattern, whereas the opposite tendency was defined as a negative interaction pattern. A negative association was observed between the embedding-distance difference and the symptom-recovery difference, with herb pairs represented relatively closer to the symptom tending to show more favourable paired recovery outcomes. For panels A, B, and D, black curves show descriptive LOWESS trends (smoothing span = 1.0), with shaded regions indicating confidence intervals.

## Figure S25 Consistency of TCM-ES geometry with observed co-occurrence patterns using NPMI.

As a sensitivity analysis of the co-occurrence relationships shown in Figure S24A-B, normalized pointwise mutual information (NPMI) was used as an alternative measure of pairwise association.

(A) Symptom–symptom embedding distances were compared with NPMI-based symptom co-occurrence associations in general TCM clinical cases, long-COVID cases, and disease-associated symptom data.

(B) Herb–herb embedding distances were compared with NPMI-based herb co-prescription associations in general TCM and long-COVID clinical cases. In both analyses, closer embeddings remained associated with stronger observed co-occurrence or co-prescription relationships, consistent with the results obtained using the Jaccard index in Figure S24. Black curves and shaded regions show LOWESS trends (smoothing span = 1.0) and confidence intervals.

## Figure S26 Formula–symptom ranking in clinical cases and sensitivity to symptom frequency.

In the 2488 eligible general TCM clinical cases, symptoms present at the initial visit and subsequently documented as alleviated at follow-up were defined as positive symptoms, whereas the remaining initial-visit symptoms were defined as negative symptoms. Positive and negative symptoms were ranked according to their embedding proximity to the prescribed formula using TCM-ES or the corresponding co-occurrence-based embeddings.

(A) Overall within-case ranking performance, quantified by case-balanced pairwise AUC, representing the probability that an alleviated symptom was ranked above a non-alleviated symptom from the same case.

(B) Frequency-matched within-case ranking, in which each positive symptom was matched to the same-case negative symptom with the closest frequency in the TCM-ES training data and evaluated under increasingly strict log-frequency calipers.

(C) Difference in individual-symptom AUC between TCM-ES and the co-occurrence SVD embeddings across symptom-frequency octiles; positive values indicate higher AUC for TCM-ES. Error bars in panel A and B indicate 95% confidence intervals obtained from 1000 bootstrap resamples of the contributing clinical cases, and points in panel C represent individual symptoms.

## Figure S27 Sensitivity analyses of the adjusted patient-condition–formula association in COVID-19 cases.

The primary analysis examined whether patient-condition–formula proximity at the initial visit was associated with subsequent condition improvement in 2132 COVID-19 cases. Patient-condition–formula distance was defined as the Euclidean distance between the initial symptom-pattern embedding and the prescribed-formula embedding in TCM-ES and was standardized within each prescribed-formula group. Condition improvement was quantified as the reduction in total symptom score from the initial to follow-up visit. The association was estimated using multivariable regression accounting for initial total symptom score, initial symptom count, follow-up interval, age, sex, and prescribed-formula differences. The analyses shown here examined the sensitivity of this association to follow-up interval, patient characteristics, and prescribed formula.

(A) Rolling time-window analyses across overlapping initial-to-follow-up intervals; the estimate based on all 2132 observed cases is shown for reference.

(B) Patient-subgroup analyses stratified by sex, age, and initial symptom severity.

(C) Separate adjusted analyses within each of the four principal prescribed-formula groups. Squares indicate adjusted regression coefficients per one-standard-deviation increase in within-formula patient-condition–formula distance, and horizontal lines indicate HC3 heteroskedasticity-robust 95% confidence intervals. Negative coefficients indicate that greater patient-condition–formula distance was associated with less subsequent condition improvement.

## Figure S28 Sensitivity of PC-associated GO-module composition to the number of selected herbs.

The normalized within-PC proportions of three literature-supported GO Biological Process modules were recalculated using the top 100, 150, 200, 250, and 300 herbs ranked along (A) PC1, (B) PC2, and (C) PC3. Points and error bars represent the mean and standard deviation across 10 independently trained and PC-aligned models, respectively. The dashed horizontal line indicates equal representation of the three modules (33.3%). Across the evaluated top-*k* thresholds, the thermogenic and mitochondrial oxidative metabolism module remained most prominent on PC1, the airway innate-inflammatory response module on PC2, and the fatty-acid oxidation and cholesterol handling module on PC3.

## Figure S29 Connectivity of disease-associated gene and herb-target modules in the human PPI network.

(A) Distribution of giant-component (GC) size *z*-scores for disease-associated gene sets relative to 1000 random gene sets containing the same number of genes.

(B) Distribution of GC size *z*-scores for herb-target sets relative to 1000 random protein sets of the corresponding sizes. The observed distributions were shifted toward positive *z*-scores, indicating that disease-associated genes and herb targets tended to form larger connected components in the human protein–protein interaction (PPI) network than size-matched random sets. These results support the treatment of the corresponding gene and target sets as network modules in the subsequent PPI-proximity analyses.

## Figure S30 Local concordance between TCM-ES neighbourhoods and PPI-network proximity.

(A-C) Local relationships were examined for herb–herb (A), disease–disease (B), and disease–herb (C) pairs, respectively. For each herb or disease, other entities were ranked by their distance in TCM-ES, and the nearest *k* = 5, 10, 20, or 50 entities were compared with all remaining entities. PPI-network relationships were quantified using standardized module separation between the corresponding herb-target or disease-associated gene sets, with lower values indicating greater molecular-network proximity. For example, in the herb–herb analysis, we asked whether the targets of the nearest herbs in TCM-ES were also closer in the PPI network than the targets of the remaining herbs. The plotted value is the difference between the distance-weighted mean PPI separation of the top-$\text{k}$ TCM-ES neighbours and the mean separation of the remaining entities. Negative values therefore indicate that entities located closer in TCM-ES are also closer in the PPI network. Coloured circles and error bars show the observed mean differences and 95% confidence intervals obtained from 1000 bootstrap resamples. Grey squares and intervals show results from 1000 permutations with identity shuffling and degree-preserving PPI permutation. The blue dashed line indicates zero difference. *p* values are from one-sided empirical permutation tests. Across all three relationship types, local TCM-ES neighbours showed greater PPI-network proximity than the remaining entities across multiple neighbourhood sizes, whereas the corresponding permutation effects were centred near zero.

## Figure S31 Training-seed stability of embedding–PPI concordance.

Ten leave-one-seed-out median-rank consensuses were constructed by excluding one repeat model at a time and taking the median embedding-distance percentile for each identical entity pair across the remaining nine models. Rows correspond to the omitted training seed, and columns show herb–herb, disease–disease, disease–herb, and protein–protein relationships. For the first three relationships, points and error bars show binned mean standardized PPI separation and its standard error, and solid lines show the corresponding leave-one-seed-out trends. For protein–protein relationships, bars show mean embedding-distance percentiles across PPI shortest-path groups. Blue dashed lines show the main-model relationships as visual references only.

## Figure S32 Diseases with similar MeSH classifications are positioned closer in TCM-ES.

Embedding distances were compared between disease pairs with similar and distinct MeSH classifications as defined in Note S8. Disease pairs with similar MeSH classifications showed shorter embedding distances. Violin plots show the distributions with embedded boxplots. *p* value was obtained using a two-sided paired *t*-test. **** *p* < 0.0001.

## Figure S33 Robustness of the TCM-ES disease network to the number of nearest neighbours.

*k*-nearest-neighbour disease networks were reconstructed using (A) *k* = 6 and (B) *k* = 8 nearest neighbours based on disease embedding proximity in TCM-ES. The overall clustering structure was similar across the two settings. Nodes represent diseases; node shapes indicate MeSH categories, and node colours reflect their positions along the leading TCM-ES principal components. Edge width increases with embedding proximity. Grey edges connect disease pairs sharing annotated genes or symptoms, whereas red edges indicate neighbouring disease pairs sharing neither.

## Figure S34 Exploratory drug–disease relationships in TCM-ES and their association with a measure derived from the FDA Adverse Event Reporting System (FAERS).

(A) Distribution of supported modern-drug mappings and their indicated diseases in TCM-ES. Representative indication pairs with relatively close, intermediate, and distant embedding relationships are highlighted.

(B) Distribution of embedding-distance *z*-scores for known drug–disease indication pairs. Negative values indicate that indicated drugs are positioned closer to their corresponding diseases than expected from the reference drug–disease relationships.

(C) Association between drug–disease bidirectional *z*-score (BDZ) and the FAERS-derived RE score. Lower BDZ values indicate closer mutual matching between a drug and disease in TCM-ES. Black curves and shaded regions show LOWESS trends (smoothing span = 1.0) and confidence intervals. A negative association was observed between BDZ and the FAERS-derived observational score.

(D) Disease-level comparison of BDZ values for indicated drugs (top) and their corresponding FAERS-derived RE scores (bottom). Only diseases with at least three drugs with available RE scores are shown.

(E) Relationship between the number of the most frequently reported adverse events for each drug in FAERS and its estimated prescription volume. These analyses are exploratory and describe drug–disease matching in TCM-ES and its association with a FAERS-derived observational measure.

## Figure S35 Sensitivity analyses of exploratory drug–disease relationships in TCM-ES.

The analyses of drug–disease indication pairs in Figure S34 were repeated under four alternative settings.

(A-D) Distributions of embedding-distance *z*-scores for indicated drug–disease pairs.

(A) A relaxed drug-screening criterion retained 186 indicated drug–disease pairs involving 134 drugs and 29 diseases.

(B) A stricter SMILES-reconstruction criterion retained 135 pairs involving 102 drugs and 27 diseases.

(C) Disease embeddings were recalculated using refined disease-associated symptoms from Mayo Clinic, yielding 125 pairs involving 94 drugs and 27 diseases.

(D) The primary drug–disease set was reanalysed using updated adverse-event reports from the FDA Adverse Event Reporting System (FAERS), accessed through openFDA. Negative *z*-scores indicate that indicated pairs are positioned closer in TCM-ES than expected from the reference drug–disease relationships. Dashed lines indicate zero. *p* values were obtained from paired two-sided *t*-tests, as shown in each panel.

(E-H) Associations between drug–disease bidirectional *z*-score (BDZ) and the FAERS-derived RE score under the four corresponding settings shown in A-D, respectively. Lower BDZ values indicate closer mutual matching between a drug and disease in TCM-ES. Points and error bars summarize the observed data across BDZ bins, while black curves and shaded regions show LOWESS trends (smoothing span = 1.0) and confidence intervals. Across the four alternative settings, indicated drug–disease pairs remained predominantly closer than expected, and lower BDZ values remained associated with higher FAERS-derived RE scores. These analyses are exploratory, and the RE score is treated as an indirect observational measure rather than a validated quantitative measure of therapeutic efficacy.

## Figure S36 TCM-informed characterization of Cold- and Heat-pattern RA groups reported in a previous study of 21 female patients.

Cold- and Heat-type rheumatoid arthritis (RA) were embedded in TCM-ES based on their reported TCM symptom profiles and visualized along PC1 and PC2. Squares show symptoms associated with the two subtypes, with colours indicating Heat-related, Cold-related, or shared symptoms; dashed ellipses summarize the corresponding symptom distributions. Six genes previously reported to be associated with the RA subtypes were mapped into TCM-ES, including MSH6 for Cold-type RA and CTBP2, GLUL, PIGK, DIABLO, and GABBR1 for Heat-type RA. RA-indicated drugs are shown for exploratory comparison. The two subtype embeddings occupy different regions of TCM-ES and are associated with different neighbouring molecular and therapeutic entities, illustrating how more detailed TCM symptom characterization can provide a complementary view of disease heterogeneity.
